# The relationship between lifestyle factors and outcome of treatment with TNFα inhibitors in axial spondyloarthritis – results from 14 European countries

**DOI:** 10.1186/s41927-025-00529-4

**Published:** 2025-07-11

**Authors:** Gareth T. Jones, Ovidiu Rotariu, Ross MacDonald, Brigitte Michelsen, Bente Glintborg, Irene van der Horst-Bruinsma, Bjorn Gudbjornsson, Arni Jon Geirsson, Heikki Relas, Pia Isomäki, Jakub Závada, Karel Pavelka, Ziga Rotar, Matija Tomšič, Michael J. Nissen, Adrian Ciurea, Catalin Codreanu, Johan K. Wallman, Eirik Klami Kristianslund, Simon Horskjaer Rasmussen, Lykke Midtbøll Ørnbjerg, Maria José Santos, Mikkel Østergaard, Merete Lund Hetland, Gary J. Macfarlane

**Affiliations:** 1 Aberdeen Centre for Arthritis and Musculoskeletal Health (Epidemiology Group), University of Aberdeen, Aberdeen, UK; 2 Center for Treatment of Rheumatic and Musculoskeletal Diseases (REMEDY), Diakonhjemmet Hospital, Oslo, Norway; 3 Research Unit, Sørlandet Hospital, Kristiansand, Norway; 4 Copenhagen Center for Arthritis Research (COPECARE), Center for Rheumatology and Spine Diseases, Centre of Head and Orthopedics, Rigshospitalet, Glostrup, Denmark; 5 Department of Clinical Medicine, Faculty of Health and Medical Sciences, University of Copenhagen, Copenhagen, Denmark; 6 Department of Rheumatology, Radboud University Medical Centre, Nijmegen, The Netherlands; 7 Faculty of Medicine, University of Iceland, Reykjavik, Iceland; 8 Centre for Rheumatology Research (ICEBIO), Landspitali University Hospital, Reykjavik, Iceland; 9 Department of Rheumatology, Landspitali University Hospital, Reykjavik, Iceland; 10 Rheumatology, Inflammation Center, Helsinki University Hospital and University of Helsinki, Helsinki, Finland; 11 Centre for Rheumatology, Tampere University Hospital, Tampere, Finland; 12 Faculty of Medicine and Health Technology, Tampere University, Tampere, Finland; 13 Institute of Rheumatology, Prague, Czech Republic; 14 Department of Rheumatology, First Faculty of Medicine, Charles University, Prague, Czech Republic; 15 Department of Rheumatology, University Medical Centre Ljubljana, Ljubljana, Slovenia; 16 Faculty of Medicine, University of Ljubljana, Ljubljana, Slovenia; 17 Department of Rheumatology, Geneva University Hospital, Geneva, Switzerland; 18 Department of Rheumatology, Zurich University Hospital, University of Zurich, Zurich, Switzerland; 19 Center for Rheumatic Diseases, University of Medicine and Pharmacy, Romanian Registry of Rheumatic Diseases, Bucharest, Romania; 20 Lund University, Lund, Sweden; 21 Skåne University Hospital, Lund, Sweden; 22 Rheumatology Research Unit, Instituto de Medicina Molecular, Faculdade de Medicina de Lisboa, Lisbon, Portugal; 23 Serviço de Reumatologia, Hospital Garcia de Orta, Reuma. pt, Almada, Portugal

**Keywords:** Axial spondylarthritis, Lifestyle, Smoking, Alcohol, Body mass index

## Abstract

**Objectives:**

To quantify the influence of lifestyle factors on tumour necrosis factor inhibitor (TNFi) treatment response, in axial spondyloarthritis (axSpA).

**Methods:**

Data on biologics-naïve adults with axSpA were captured from European rheumatology registries. Information on lifestyle factors (smoking, overweight/obesity, and/or alcohol consumption) were identified ± 30 days of commencing their first TNFi. Treatment response (BASDAI-50, ASDAS or ASAS response criteria) was determined at 3 and 12 months. In separate models, the relationship between treatment response and baseline smoking, BMI and alcohol was assessed using logistic regression, adjusted for age, sex, country, calendar year of treatment initiation, disease duration and baseline disease activity.

**Results:**

From 14 registries, 14,885 patients were included. Of those with available data, 29% were current smokers, 49% current drinkers, 37% were overweight and 21% were obese. At 12 months, smokers were less likely to achieve BASDAI-50 treatment response compared to non-smokers (adjusted odds ratio: 0.77; 95%CI: 0.68–0.86). A similar effect was observed among overweight (0.76; 0.66–0.87) or obese patients (0.53; 0.45–0.63). In contrast, alcohol drinkers experienced a seemingly beneficial effect (1.47; 1.16–1.87). These associations were also observed with other measures of treatment response and were robust to further adjustment for clinical characteristics.

**Conclusion:**

Smoking and high BMI decrease the odds of bDMARD treatment success in axSpA. Rheumatologists should consider referral to smoking cessation and/or weight management interventions at the time of commencing therapy, to enhance treatment response. The relationship between alcohol and treatment response is unlikely to be causal and warrants further investigation.

**Supplementary Information:**

The online version contains supplementary material available at 10.1186/s41927-025-00529-4.

## Key messages

**What is already known about this subject?**

- It has been shown that adverse lifestyle factors (smoking, high body mass index, alcohol consumption) are associated with poorer outcomes in axial spondyloarthritis, but there are no robust estimates that quantify the impact on anti-TNF treatment response.

**What does this study add?**

- Our findings, pooling data from 14 national registries, demonstrate that patients who smoke experience a 20–25% decrease in the likelihood of meeting treatment response criteria, 12 months after commencing their first TNF inhibitor, an effect that was consistent across multiple outcome measures.
- Overweight/obese patients experience a 50% decrease in the odds of meeting such treatment criteria.
- For alcohol consumption, results were reversed, with current alcohol-users more likely to experience positive outcomes. This result was not supported by sensitivity analysis imputing missing values, and thus warrants further investigation.

**How might this impact on clinical practice or future developments?**

- This paper quantifies the deleterious effect of smoking and obesity on the likelihood of treatment response in axial spondyloarthritis. As an adjunct to pharmacological management, lifestyle interventions might be considered as a way of optimising patient outcomes.

## Introduction

Axial spondyloarthritis (axSpA) is an inflammatory arthritis affecting the spine and sacroiliac joints principally, although peripheral arthritis and extra musculoskeletal manifestations (uveitis, psoriasis and inflammatory bowel disease) are not uncommon. Over the last two decades, much research has focused on pharmacological management, in particular, the use of biologic disease-modifying anti-rheumatic drugs (bDMARDs) although recent management guidelines recommend that optimal management requires ‘a combination of non-pharmacological and pharmacological treatment modalities’ [1]. Lifestyle changes– for example, smoking cessation, losing weight, and/or reducing alcohol consumption– also offer the potential to enhance outcomes further. However, there is little evidence quantifying their potential impact on treatment outcomes. Being able to quantify the benefits to patients may help facilitate lifestyle changes.

In axSpA, smoking has been shown to be associated with higher disease activity and poorer function [2], and shorter treatment adherence [3], but the effect on treatment outcomes is less clear. Fewer data are available with respect to alcohol consumption and body mass index (BMI). A recent cross-sectional study found that patients with moderate alcohol consumption were more likely to have favourable disease status, although from this it is not possible to disentangle whether alcohol consumption is causally related to disease status or whether persons with such disease status are more likely to undertake their usual activities [4]. We are not aware of any studies specifically examining the effect of alcohol consumption on bDMARD treatment response in axSpA. For BMI, there have only been a few studies, mainly cross-sectional or retrospective, and each with a small number of patients which have described ‘poor clinical outcome’ associated with high BMI [5–8], and some have shown that overweight / obese patients experience an increase in the odds of radiographic progression [9]. A recent meta-analysis suggested that obesity is a predictor of response to TNFi therapy across a range of immune-mediated inflammatory diseases [10], and a sub-analysis of six studies in spondyloarthritis (966 patients) found that obesity was associated with a three-fold increase in the odds of non-response to therapy, but with considerable uncertainty (odds ratio: 3.4; 95%CI 1.3–8.5), and it’s unclear to what extent potential confounding factors were controlled for.

Many studies (e.g. based on claims databases) have little or no data on lifestyle factors. Quantifying the effect of lifestyle on treatment outcome is important to give some information on the potential benefits that might be achieved through lifestyle changes and to gain better insight into the complexity of the still sparsely understood factors that drive treatment effectiveness. The EuroSpA Research Collaboration Network is a large ongoing collaboration of researchers from across Europe, pooling data from country-specific SpA registries (https://eurospa.eu). Using data from EuroSpA, the aim of the current study was to quantify the effects of smoking, BMI and alcohol consumption on the TNFi treatment response, amongst biologics naïve patients with axSpA.

## Patients and methods

Fourteen of the sixteen registries in the EuroSpA collaboration had data on smoking, BMI and/or alcohol consumption among patients with axSpA and agreed to participate in the current analysis: AmSpA (Netherlands); ATTRA (Czech Republic); BIOBADASER (Spain); BIORX.SI (Slovenia); BSRBR-AS (United Kingdom); DANBIO (Denmark); GISEA (Italy); ICEBIO (Iceland); NOR-DMARD (Norway); REUMA.PT (Portugal); ROB-FIN (Finland); SCQM (Switzerland); TURKBIO (Turkey) and the Romanian registry of Rheumatic Diseases. Data collection procedures varied between countries, but all registries collected data prospectively from medical records, as part of routine care, and/or patient questionnaires. Anonymised data from each registry was uploaded to servers at the EuroSpA Coordinating Centre in Copenhagen, on which all analyses took place. The study was approved by the respective national Data Protection Agencies and Research Ethical Committees according to legal regulatory requirements in the participating countries and was performed in accordance with the Declaration of Helsinki.

### Inclusion criteria

Patients, recruited to the individual registries from 2000 to 2020, were required to have a clinical diagnosis of ankylosing spondylitis or axSpA, or to meet modified New York criteria for ankylosing spondylitis [11] and/or ASAS criteria for axSpA [12]. Patients were required to be aged ≥ 18yrs; to be commencing their first TNFi therapy; to have information regarding at least one of the lifestyle factors of interest (smoking, BMI, alcohol consumption) recorded within a window of ± 30 days of the start of TNFi therapy; and to have sufficient data to determine at least one of the outcomes of interest at 3 and/or 12 months after commencing therapy.

### Baseline data collection

The baseline lifestyle factors included within the current study were smoking status, BMI and alcohol consumption. Data on the quantity of smoking and alcohol use was unavailable for most registries and therefore these variables were examined as self-reported current versus non-current use. BMI was categorised as underweight (< 18 kg/m^2^), normal weight (18–25 kg/m^2^), overweight (25–30 kg/m^2^) and obese (> 30 kg/m^2^).

Additional information included demographic characteristics (age and sex), clinical characteristics (symptom/disease duration, age at symptom start/diagnosis, HLA-B27 status, acute phase reactants (ESR/CRP), the history of extra-musculoskeletal manifestations (inflammatory bowel disease, uveitis, and psoriasis) and whether there was any evidence of various comorbidities (cardiovascular disease, kidney disease, and diabetes). Disease activity measures included the Bath Ankylosing Spondylitis indices for disease activity (BASDAI [13]) and function (BASFI [14]) each scored from 0 (best) to 100 (worst) and the Bath Ankylosing Spondylitis Metrology Index (BASMI [15]) scored from 0 (best) to 10 (worst). Fatigue and pain were measured via visual analogue scales (0 (best) to 100 (worst)) and physical function by the Health Assessment Questionnaire (HAQ; 0 (best) to 3 (worst)) [16]. All patients were commencing TNFi therapy; concomitant therapy with conventional synthetic DMARDs (csDMARDs) and/or non-steroidal anti-rheumatic drugs (NSAIDs) was also recorded. Precisely what information was available varied between registries.

### Treatment response

Treatment response was assessed using different response criteria: (1) 50% improvement in BASDAI score (BASDAI-50); (2) Ankylosing Spondylitis Disease Activity Score (ASDAS) inactive disease (< 1.3; ASDAS-ID), low disease activity (< 2.1; ASDAS-LDA), clinically important improvement (reduction of ≥ 1.1 units, ASDAS-CII) and major improvement (reduction of ≥ 2.0 units, ASDAS-MI) [17–19]; and (3) ASAS-20, ASAS-40 and ASAS-5/6 improvement criteria [20, 21]. Each outcome was assessed 3 and 12 months after commencing TNFi^1^.

### Statistical analysis

Baseline characteristics were described using simple descriptive statistics appropriate to data type (median (inter-quartile range (IQR)) for continuous/count data and proportion for categorical variables). Thereafter, the relationship between lifestyle factors and treatment response was determined using logistic regression models. Thus, results are expressed as odds ratios (OR) with 95% confidence intervals (95%CI).

In the first instance, a univariable model (Model 1) was constructed to determine the association between smoking at baseline and the odds of satisfying the BASDAI-50 response criteria at 3 months. To control for potential confounding, this was adjusted for age, sex, country, calendar year, disease duration and baseline disease activity (BASDAI) (Model 2). This model was then further adjusted for alcohol consumption (Model 3a) and BMI (Model 3b). Full multivariable models containing these plus all other potential confounding variables were not computed for several reasons. Firstly, several variables had high amounts of missing data– including some data missing by design, where certain variables were not available in certain registries. And secondly, we considered it methodologically inappropriate to adjust for all available variables simultaneously because there was evidence of collinearity between some of the variables which might lead to statistical instability in the models. Therefore, instead, a sensitivity analysis was conducted, Model 2 was adjusted for each additional baseline variable one-by-one. This allowed the examination of any additional confounding effect of each variable in turn, while also permitting each model to have the maximum number of participants. The following variables were considered: fulfilment of classification criteria (modified New York criteria, ASAS criteria); years since symptom onset, clinical measures (BASFI and BASMI), patient and physician global scores, pain, fatigue, disability (HAQ) and HLA-B27 status); acute phase reactants (CRP, ESR); extra-musculoskeletal manifestations (inflammatory bowel disease, uveitis and psoriasis); comorbidities (cardiovascular disease, kidney disease and diabetes); and concomitant medications (csDMARDs, NSAIDs).

The above process was then repeated firstly for smoking with respect to 12-month outcome, and then for each other measure of response criteria. Finally, the whole process was repeated for BMI and alcohol consumption at each time point and with each outcome.

All analyses were conducted using R v3.4.3 software [22]. Data analysis was conducted at the University of Aberdeen, UK, on data stored on the EuroSpA analysis server provided by ZiteLab ApS with server-hosting in Copenhagen, Denmark.

## Results

From 14 countries, the dataset comprised 16,874 patients of whom 14,885 had data on at least one lifestyle factor. There was a large variation in the number of patients included from each registry (range: Iceland *N* = 197; Denmark *N* = 4,390). Information on baseline smoking status was available for 80% of patients (*N* = 13,567; 13 registries) and BMI for 58% (*N* = 9,864; 13 registries). Only 22% of patients (*N* = 3,665; 7 registries) had information on alcohol consumption. Table 1 shows the number of patients who contributed lifestyle data per country.

**Table 1:** Availability of lifestyle data

Registry	Total *N*^1^	Lifestyle data available on…				
		Smoking	BMI	Alcohol	Any^2^	All^3^
**ATTRA** (Czech Republic)	2422	1,335	1,415	–	1,446	–
**DANBIO** (Denmark)	4390	4,281	2,275	1,917	4,309	1801
**BIOBADASER** (Spain)	364	343	280	–	352	–
**ICEBIO** (Iceland)	197	121	60	50	124	49
**GISEA** (Italy)	1336	641	1,088	102	1,189	75
**AmSpA** (Netherlands)	264	–	256	–	256	–
**NOR-DMARD** (Norway)	1717	1,474	–	–	1,474	–
**REUMA.PT** (Portugal)	1234	995	314	940	1,026	274
**Romanian Registry of Rheumatic Diseases** (Romania)	676	676	675	–	676	–
SCQM **(Switzerland**)	1033	1,011	981	21	1,029	20
**ROB-FIN** (Finland)	716	244	560	–	560	–
**BIORX.SI** (Slovenia)	550	549	540	–	550	–
**TURKBIO** (Turkey)	1347	1,265	890	13	1,267	13
**BSRBR-AS** (Great Britain)	628	622	530	622	627	524
**All registries**	16,874	13,557	9,864	3,665	14,885	2,756

^1^ Patients with data for at least one outcome variable

^2^ Any of the three lifestyle variables

^3^ All of the three lifestyle variables

Baseline patient characteristics are shown in Table 2. Of those with available data, 29% were current smokers, whilst 49% were current alcohol drinkers. Fewer than half of patients (40.9%) had a normal BMI, 1.4% were underweight (BMI < 18 kg/m^2^) and were combined with normal weight for the analysis, 36.8% of patients were overweight and 20.9% were obese. Approximately 60% were male; median age at the time of commencing TNFi was 42yrs (IQR: 34-52yrs) and median disease duration was 4yrs (IQR: 1-10yrs). Of those with available data, 75% of patients were HLA-B27 positive, 96% satisfied the ASAS classification criteria for axSpA and 72% met the modified New York criteria for ankylosing spondylitis. Median disease activity (BASDAI) score was 57.5 (IQR: 40.6–71.4).

**Table 2:** Participants’ baseline characteristics

Baseline characteristics		*N*	(%)	*N*	*N*
		or Median (IQR)		Participants	Registries
***Lifestyle characteristics***					
Current smoker	Yes	3,937	(29.0%)	13,557	13
Current drinker	Yes	1,789	(48.8%)	3,665	7
Body mass index^1^	Overweight	3,639	(36.9%)	9,864	13
	Obese	2,065	(20.9%)		
***Demographic characteristics***					
Age at diagnosis	Years	35	(27–44)	14,074	14
Age at start of first TNFi	Years	42	(34–52)	14,870	14
Sex	Male	8,960	(60.2%)	14,874	14
***Clinical characteristics***					
Symptom duration	Years	10	(4–18)	9,855	10
Time since diagnosis^2^	Years	3	(1–9)	14,084	14
HLA-B27	Positive	6,827	(75.3%)	9,068	14
ESR (ln)	mm/hour	3.00	(2.20–3.56)	6,862	11
CRP (ln)	mg/litre	2.16	(1.10–3.05)	10,354	12
Modified New York criteria	Positive	3,937	(71.5%)	5,505	10
ASAS axSpA criteria	Positive	7,071	(95.7%)	7,385	10
BASDAI	0-100	57.9	(41.1–71.5)	10,896	14
BASFI	0-100	47.1	(26.9–67.0)	8,901	11
BASMI	0–10	2.0	(1.0–4.0)	2,017	4
Patient global score	0-100	68.0	(49.0–80.0)	11,089	14
Physician global score	0-100	40.0	(20–60)	8,294	10
Pain	0-100	65.0	(44.0–80.0)	10,700	14
Fatigue	0-100	70.0	(49.0–80.0)	10,062	12
HAQ	0–3	0.75	(0.50–1.25)	7,286	9
***Extra-axial manifestations and comorbidities***					
Inflammatory bowel disease	Yes	787	(9.4%)	8,401	13
Uveitis	Yes	1,644	(19.2%)	8,575	12
Psoriasis	Yes	689	(8.4%)	8,197	12
Cardiovascular disease	Yes	1,202	(17.4%)	6,904	13
Kidney disease	Yes	167	(2.5%)	6,602	11
Diabetes	Yes	444	(6.4%)	6,938	13
***Concomitant medications***					
csDMARDs	Yes	4,519	(60.8%)	7,434	14
NSAIDs	Yes	2,940	(68.7%)	4,282	9

Abbreviations: TNFi: Tumour Necrosis Factor α inhibitor. HLA-B27: Human Leucocyte Antigen B27. ESR: Erythrocyte Sedimentation Rate. ln: Natural Logarithm. CRP: C-reactive Protein. BASDAI: Bath Ankylosing Disease Activity Index. BASFI: Bath Ankylosing Functional Index. BASMI: Bath Ankylosing Metrology Index. HAQ: Health Assessment Questionnaire. csDMARDs: Conventional synthetic Disease Modifying Anti-Rheumatic Drugs. NSAIDs: Non-steroidal anti-inflammatory drugs

^1^ Overweight (25–30 kg/m^2^), obese (> 30 kg/m^2^)

^2^ Data for UK (BSRBR-AS) used ‘date patient first seen by a rheumatologist’ as a proxy for date of diagnosis

The number of patients with outcome data varied from 4,506 (30%) with ASAS-5/6 data at 12 months, to 7,465 (50%) with information to derive BASDAI-50 at 12 months, see Table 3. At 3 months, the proportion of patients with a positive treatment response ranged from 20.1% (ASAS-40) to 50.5% (ASDAS-LDA). Across all outcomes, the proportion meeting treatment response criteria was higher at 12 months: 30.5% and 60.9% met the ASAS-40 and ASDAS-LDA criteria, respectively.

**Table 3:** Outcomes at 3 and 12 months among 14,885 participants with data on ≥ 1 lifestyle variable

Response criteria		3 months		12 months	
		*N*	(%)^1^	*N*	(%)^1^
BASDAI-50	No	3,953	(58.8%)	3,552	(47.6%)
	Yes	2,768	(41.2%)	3,913	(52.4%)
	Missing	8,164		7,420	
ASDAS-ID	No	5,156	(77.2%)	5,180	(70.7%)
	Yes	1,524	(22.8%)	2,147	(29.3%)
	Missing	8,205		7,558	
ASDAS-LDA	No	3,309	(49.5%)	2,862	(39.1%)
	Yes	3,371	(50.5%)	4,465	(60.9%)
	Missing	8,205		7,558	
ASDAS-CII	No	2,496	(50.8%)	1,976	(39.5%)
	Yes	2,416	(49.2%)	3,031	(60.5%)
	Missing	9,973		9,878	
ASDAS-MI	No	3,662	(74.6%)	3,239	(64.7%)
	Yes	1,250	(25.4%)	1,768	(35.3%)
	Missing	9,973		9,878	
ASAS-20	No	3,477	(64.5%)	2,826	(52.4%)
	Yes	1,916	(35.5%)	2,567	(47.6%)
	Missing	9,492		9,492	
ASAS-40	No	4,971	(79.9%)	4,284	(69.5%)
	Yes	1,251	(20.1%)	1,882	(30.5%)
	Missing	8,663		8,719	
ASAS-5/6	No	3,504	(77.0%)	2,878	(63.9%)
	Yes	1,048	(23.0%)	1,628	(36.1%)
	Missing	10,333		10,379	

^1^ Percentages of non-missing values

### Smoking

Current smoking was associated with a decrease in the odds of achieving BASDAI-50 response criteria at 3 months, an association that was robust to statistical adjustment for age, sex, country, calendar year, disease duration and baseline disease activity (OR: 0.83; 0.73–0.93) (*N* = 6284). This association persisted until 12 months (0.77; 0.68–0.86) (*N* = 6886) and was strengthened by further adjustment for alcohol consumption and BMI, see Table 4. The effect was less strong, and not statistically significant, with ASDAS-CII and ASDAS-MI although, generally, the same relationship was observed across all outcome measures: smoking was associated with a modest reduction in the odds of achieving treatment response criteria.

**Table 4:** The effect of smoking on the odds of meeting treatment response criteria (current smokers versus ex- and never-smokers combined)

	Odds ratio; 95% confidence interval			
	Model 1	Model 2	Model 3a / 3b	Sensitivity analysis
**3-month outcome**				
N ^1^	4183 to 6293	3759 to 6284	1234 to 3263	1024 to 6035
BASDAI-50	0.93; 0.83–1.04	0.83; 0.73–0.93	^3a^ 0.76; 0.61–0.95 ^3b^ 0.71; 0.60–0.84	^Lo^ 0.64; 0.51–0.78 ^Hi^ 0.85; 0.73–0.99
ASDAS-ID	0.81; 0.71–0.92	0.72; 0.62–0.84	^3a^ 0.59; 0.44–0.80 ^3b^ 0.60; 0.47–0.76	^Lo^ 0.55; 0.41–0.73 ^Hi^ 0.80; 0.66–0.97
ASDAS-LDA	0.79; 0.71–0.88	0.74; 0.65–0.85	^3a^ 0.67; 0.53–0.85 ^3b^ 0.60; 0.49–0.74	^Lo^ 0.56; 0.44–0.71 ^Hi^ 0.81; 0.63–1.04
ASDAS-CII	1.11; 0.98–1.26	0.96; 0.84–1.10	^3a^ 0.91; 0.72–1.15 ^3b^ 0.81; 0.66–0.99	^Lo^ 0.80; 0.63–1.01 ^Hi^ 1.05; 0.81–1.36
ASDAS-MI	1.07; 0.93–1.24	0.92; 0.79–1.07	^3a^ 1.01; 0.77–1.32 ^3b^ 0.84; 0.67–1.06	^Lo^ 0.60; 0.46–0.80 ^Hi^ 0.96; 0.72–1.29
ASAS-20	1.02; 0.90–1.15	0.78; 0.68–0.90	^3a^ 0.75; 0.59–0.96 ^3b^ 0.70; 0.58–0.84	^Lo^ 0.47; 0.30–0.72 ^Hi^ 0.80; 0.69–0.92
ASAS-40	1.05; 0.92–1.20	0.79; 0.68–0.91	^3a^ 0.67; 0.51–0.88 ^3b^ 0.68; 0.55–0.83	^Lo^ 0.41; 0.25–0.66 ^Hi^ 0.85; 0.66–1.10
ASAS-5/6	1.07; 0.92–1.24	0.78; 0.66–0.92	^3a^ 0.61; 0.44–0.83 ^3b^ 0.66; 0.52–0.83	^Lo^ 0.44; 0.26–0.73 ^Hi^ 0.79; 0.65–0.96
**12-month outcome**				
N ^1^	4184 to 7126	3834 to 6886	932 to 4409	912 to 6234
BASDAI-50	0.87; 0.79–0.96	0.77; 0.68–0.86	^3a^ 0.69; 0.55–0.87 ^3b^ 0.66; 0.57–0.77	^Lo^ 0.67; 0.55–0.81 ^Hi^ 0.82; 0.67–0.995
ASDAS-ID	0.76; 0.68–0.86	0.68; 0.59–0.79	^3a^ 0.65; 0.48–0.88 ^3b^ 0.58; 0.48–0.70	^Lo^ 0.56; 0.45–0.70 ^Hi^ 0.79; 0.58–1.08
ASDAS-LDA	0.82; 0.74–0.91	0.81; 0.71–0.93	^3a^ 0.73; 0.56–0.94 ^3b^ 0.72; 0.60–0.86	^Lo^ 0.73; 0.59–0.89 ^Hi^ 0.86; 0.74–0.998
ASDAS-CII	1.10; 0.97–1.25	0.95; 0.82–1.09	^3a^ 0.83; 0.64–1.08 ^3b^ 0.82; 0.67–0.99	^Lo^ 0.83; 0.71–0.97 ^Hi^ 1.06; 0.82–1.36
ASDAS-MI	1.08; 0.95 1.22	0.95; 0.82–1.10	^3a^ 1.02; 0.76–1.36 ^3b^ 0.89; 0.73–1.08	^Lo^ 0.82; 0.70–0.97 ^Hi^ 1.06; 0.87–1.29
ASAS-20	1.12; 0.99–1.26	0.87; 0.75–1.005	^3a^ 0.82; 0.62–1.08 ^3b^ 0.80; 0.67–0.96	^Lo^ 0.80; 0.69–0.94 ^Hi^ 0.94; 0.73–1.21
ASAS-40	1.10; 0.98–1.24	0.83; 0.72–0.95	^3a^ 0.72; 0.54–0.96 ^3b^ 0.75; 0.63–0.89	^Lo^ 0.65; 0.49–0.86 ^Hi^ 0.85; 0.72–0.99
ASAS-5/6	1.08; 0.94–1.23	0.82; 0.69–0.96	^3a^ 0.75; 0.54–1.04 ^3b^ 0.72; 0.59–0.88	^Lo^ 0.72; 0.60–0.86 ^Hi^ 0.86; 0.70–1.06

^1^ N is the minimum and maximum number of participants in the subsequent models

Model 1 – Crude association, unadjusted. All models present the odds ratio associated with being a current smoker, versus ex- and never-smokers combined

Model 2 – As per Model 1 but adjusted for age, sex, country, calendar year, quintiles of disease duration and baseline disease activity

Model 3a/3b – As per Model 2 but also adjusted for other lifestyle factors, denoted superscript 3a (Model 3a, adjusted for alcohol consumption) and 3b (Model 3b, adjusted for BMI categories)

Additional information, including the number of participants in each model, and a full description of Model 2, is shown in the supplementary data

Sensitivity analysis – As per Model 2 but additionally adjusted, in turn, for all other variables shown in Table 2. Results from individual models are shown in supplementary data. Here, the lowest and highest odds ratios are presented, denoted superscript Lo and Hi, respectively

### Body mass index

Compared to those of normal weight, patients who were overweight or obese experienced a significant decrease in the odds of achieving BASDAI-50 response at 3 months (0.75; 0.64–0.88 and 0.55; 0.46–0.68, respectively (*N* = 3727), after adjusting for age, sex, country, calendar year, disease duration and baseline disease activity; Table 5). This relationship remained at 12 months (0.76; 0.66–0.87 and 0.53; 0.45–0.63, respectively (*N* = 5010)). The same association was observed across all outcomes and was robust to further adjustment for other lifestyle factors, smoking and alcohol consumption.

**Table 5:** The effect of BMI on the odds of meeting treatment response criteria (overweight and obese, versus normal weight)

		Odds ratio; 95% confidence interval			
		Model 1	Model 2	Model 3a / 3b	Sensitivity analysis
**3-month outcome**					
N ^1^		2561 to 3768	2325 to 3727	1033 to 3263	571 to 3527
BASDAI-50	Overweight	0.73; 0.63–0.85	0.75; 0.64–0.88	^3a^ 0.73; 0.61–0.86 ^3b^ 0.66; 0.52–0.85	^Lo^ 0.62; 0.46–0.84 ^Hi^ 0.89; 0.72–1.10
	Obese	0.51; 0.42–0.61	0.55; 0.46–0.68	^3a^ 0.54; 0.43–0.66 ^3b^ 0.49; 0.35–0.67	^Lo^ 0.30; 0.19–0.48 ^Hi^ 0.70; 0.54–0.90
ASDAS-ID	Overweight	0.66; 0.55–0.78	0.66; 0.53–0.82	^3a^ 0.63; 0.50–0.79 ^3b^ 0.59; 0.43–0.82	^Lo^ 0.60; 0.44–0.81 ^Hi^ 0.86; 0.62–1.19
	Obese	0.29; 0.22–0.37	0.38; 0.28–0.52	^3a^ 0.37; 0.26–0.52 ^3b^ 0.30; 0.17–0.49	^Lo^ 0.28; 0.14–0.54 ^Hi^ 0.44; 0.26–0.71
ASDAS-LDA	Overweight	0.62; 0.54–0.72	0.61; 0.50–0.73	^3a^ 0.60; 0.49–0.73 ^3b^ 0.62; 0.47–0.81	^Lo^ 0.55; 0.42–0.74 ^Hi^ 0.76; 0.58–0.998
	Obese	0.35; 0.29–0.42	0.42; 0.33–0.53	^3a^ 0.40; 0.31–0.51 ^3b^ 0.43; 0.31–0.61	^Lo^ 0.35; 0.21–0.56 ^Hi^ 0.57; 0.40–0.83
ASDAS-CII	Overweight	0.83; 0.69–0.99	0.78; 0.64–0.96	^3a^ 0.76; 0.62–0.93 ^3b^ 0.81; 0.62–1.06	^Lo^ 0.68; 0.51–0.91 ^Hi^ 0.91; 0.72–1.16
	Obese	0.54; 0.44–0.66	0.50; 0.39–0.63	^3a^ 0.47; 0.36–0.60 ^3b^ 0.52; 0.37–0.72	^Lo^ 0.27; 0.17–0.43 ^Hi^ 0.67; 0.44–1.01
ASDAS-MI	Overweight	0.72; 0.60–0.88	0.66; 0.53–0.83	^3a^ 0.68; 0.54–0.86 ^3b^ 0.61; 0.44–0.84	^Lo^ 0.61; 0.41–0.90 ^Hi^ 0.80; 0.54–1.20
	Obese	0.44; 0.34–0.57	0.40; 0.29–0.53	^3a^ 0.42; 0.31–0.57 ^3b^ 0.39; 0.25–0.60	^Lo^ 0.31; 0.16–0.55 ^Hi^ 0.55; 0.37–0.83
ASAS-20	Overweight	0.79; 0.67–0.93	0.77; 0.64–0.92	^3a^ 0.78; 0.64–0.95 ^3b^ 0.81; 0.62–1.05	^Lo^ 0.64; 0.44–0.93 ^Hi^ 0.93; 0.70–1.23
	Obese	0.62; 0.51–0.75	0.58; 0.46–0.73	^3a^ 0.55; 0.43–0.70 ^3b^ 0.60; 0.43–0.82	^Lo^ 0.44; 0.28–0.68 ^Hi^ 0.87; 0.60–1.26
ASAS-40	Overweight	0.70; 0.59–0.84	0.67; 0.55–0.82	^3a^ 0.68; 0.55–0.84 ^3b^ 0.66; 0.49–0.88	^Lo^ 0.51; 0.34–0.76 ^Hi^ 0.86; 0.63–1.17
	Obese	0.51; 0.41–0.64	0.47; 0.37–0.61	^3a^ 0.48; 0.37–0.62 ^3b^ 0.53; 0.36–0.75	^Lo^ 0.25; 0.14–0.43 ^Hi^ 0.70; 0.48–1.02
ASAS-5/6	Overweight	0.71; 0.58–0.86	0.70; 0.56–0.88	^3a^ 0.72; 0.56–0.91 ^3b^ 0.73; 0.52–1.02	^Lo^ 0.68; 0.52–0.88 ^Hi^ 0.98; 0.70–1.34
	Obese	0.45; 0.36–0.58	0.45; 0.35–0.59	^3a^ 0.44; 0.32–0.59 ^3b^ 0.47; 0.31–0.72	^Lo^ 0.29; 0.16–0.52 ^Hi^ 0.73; 0.44–1.18
**12-month outcome**					
N ^1^		3160 to 5017	2819 to 5010	795 to 4409	484 to 4512
BASDAI-50	Overweight	0.76; 0.67–0.86	0.76; 0.66–0.87	^3a^ 0.75; 0.65–0.87 ^3b^ 0.63; 0.48–0.81	^Lo^ 0.65; 0.46–0.92 ^Hi^ 0.89; 0.73–1.08
	Obese	0.53; 0.45–0.61	0.53; 0.45–0.63	^3a^ 0.51; 0.43–0.61 ^3b^ 0.42; 0.30–0.59	^Lo^ 0.33; 0.20–0.52 ^Hi^ 0.64; 0.50–0.80
ASDAS-ID	Overweight	0.66; 0.58–0.76	0.73; 0.61–0.87	^3a^ 0.71; 0.59–0.86 ^3b^ 0.73; 0.52–1.02	^Lo^ 0.63; 0.40–0.97 ^Hi^ 0.81; 0.64–1.02
	Obese	0.37; 0.31–0.44	0.43; 0.34–0.55	^3a^ 0.42; 0.33–0.54 ^3b^ 0.41; 0.24–0.69	^Lo^ 0.35; 0.22–0.54 ^Hi^ 0.55; 0.40–0.76
ASDAS-LDA	Overweight	0.74; 0.65–0.84	0.76; 0.64–0.91	^3a^ 0.76; 0.63–0.92 ^3b^ 0.67; 0.50–0.90	^Lo^ 0.75; 0.63–0.91 ^Hi^ 0.88; 0.68–1.13
	Obese	0.40; 0.34–0.46	0.40; 0.32–0.49	^3a^ 0.40; 0.32–0.50 ^3b^ 0.40; 0.27–0.59	^Lo^ 0.35; 0.21–0.58 ^Hi^ 0.48; 0.36–0.63
ASDAS-CII	Overweight	0.96; 0.81–1.13	0.92; 0.75–1.12	^3a^ 0.89; 0.72–1.09 ^3b^ 0.83; 0.61–1.12	^Lo^ 0.85; 0.68–1.05 ^Hi^ 1.27; 0.93–1.74
	Obese	0.67; 0.55–0.82	0.52; 0.41–0.66	^3a^ 0.53; 0.41–0.67 ^3b^ 0.49; 0.33–0.71	^Lo^ 0.39; 0.24–0.64 ^Hi^ 0.68; 0.48–0.98
ASDAS-MI	Overweight	0.86; 0.73–1.01	0.77; 0.63–0.94	^3a^ 0.76; 0.62–0.93 ^3b^ 0.65; 0.46–0.91	^Lo^ 0.66; 0.51–0.86 ^Hi^ 0.94; 0.61–1.44
	Obese	0.63; 0.52–0.77	0.48; 0.37–0.61	^3a^ 0.48; 0.37–0.61 ^3b^ 0.51; 0.32–0.79	^Lo^ 0.39; 0.21–0.70 ^Hi^ 0.57; 0.38–0.85
ASAS-20	Overweight	0.91; 0.79–1.05	0.86; 0.71–1.02	^3a^ 0.82; 0.68–0.99 ^3b^ 0.72; 0.54–0.97	^Lo^ 0.76; 0.57–1.02 ^Hi^ 1.12; 0.85–1.48
	Obese	0.74; 0.62–0.88	0.59; 0.48–0.74	^3a^ 0.57; 0.46–0.71 ^3b^ 0.44; 0.30–0.64	^Lo^ 0.28; 0.17–0.46 ^Hi^ 0.87; 0.64–1.20
ASAS-40	Overweight	0.93; 0.81–1.07	0.94; 0.79–1.12	^3a^ 0.95; 0.79–1.14 ^3b^ 0.85; 0.63–1.15	^Lo^ 0.83; 0.56–1.24 ^Hi^ 1.24; 0.96–1.59
	Obese	0.68; 0.57–0.81	0.61; 0.49–0.75	^3a^ 0.58; 0.46–0.73 ^3b^ 0.50; 0.33–0.74	^Lo^ 0.38; 0.22–0.65 ^Hi^ 0.84; 0.62–1.13
ASAS-5/6	Overweight	0.89; 0.76–1.05	0.88; 0.72–1.08	^3a^ 0.86; 0.69–1.06 ^3b^ 0.69; 0.48–0.98	^Lo^ 0.77; 0.49–1.21 ^Hi^ 1.24; 0.92–1.66
	Obese	0.67; 0.55–0.81	0.56; 0.44–0.72	^3a^ 0.55; 0.43–0.72 ^3b^ 0.47; 0.29–0.74	^Lo^ 0.37; 0.20–0.65 ^Hi^ 0.75; 0.53–1.05

^1^ N is the minimum and maximum number of participants in the subsequent models

Model 1– Crude association, unadjusted. All models present the odds ratio associated with being overweight (25–30 kg/m^2^) or obese (> 30 kg/m^2^), versus normal weight (< 25 kg/m^2^)

Model 2– As per Model 1 but adjusted for age, sex, country, calendar year, quintiles of disease duration and baseline disease activity

Model 3a/3b– As per Model 2 but also adjusted for other lifestyle factors, denoted superscript 3a (Model 3a, adjusted for smoking) and 3b (Model 3b, adjusted for alcohol consumption)

Additional information, including the number of participants in each model, and a full description of Model 2, is shown in the supplementary data

Sensitivity analysis– As per Model 2 but additionally adjusted, in turn, for all other variables shown in Table 2. Results from individual models are shown in supplementary data. Here, the lowest and highest odds ratios are presented, denoted superscript Lo and Hi, respectively

### Alcohol consumption

Across all outcome measures there was a consistent effect suggesting that current drinkers experienced an increase in the odds of achieving treatment response, see Table 6. For example, current drinkers experienced a 47% increase in the odds of meeting BASDAI-50 response criteria at 3 months (1.47; 1.18–1.85) (*N* = 1926), an effect that persisted until 12 months (1.47; 1.16–1.88) (*N* = 1614) and was robust to adjustment for smoking and BMI (1.46; 1.15–1.87 (*N* = 1610), and 1.50; 1.15–1.96 (*N* = 1267), respectively).

**Table 6:** The effect of alcohol consumption on the odds of meeting treatment response criteria (current drinkers versus ex- and never-drinkers combined)

	Odds ratio; 95% confidence interval			
	Model 1	Model 2	Model 3a / 3b	Sensitivity analysis
**3-month outcome**				
N ^1^	1415 to 2179	1237 to 1926	1033 to 1920	322 to 1896
BASDAI-50	1.17; 0.97–1.41	1.47; 1.18–1.85	^3a^ 1.47; 1.17–1.84 ^3b^ 1.56; 1.22-2.00	^Lo^ 0.96; 0.58–1.59 ^Hi^ 1.72; 1.11–2.67
ASDAS-ID	1.34; 1.08–1.66	1.46; 1.08–1.97	^3a^ 1.45; 1.07–1.96 ^3b^ 1.45; 1.04–2.01	^Lo^ 0.99; 0.51–1.88 ^Hi^ 2.45; 1.38–4.44
ASDAS-LDA	1.41; 1.19–1.68	1.56; 1.23-2.00	^3a^ 1.54; 1.21–1.97 ^3b^ 1.62; 1.24–2.13	^Lo^ 1.07; 0.70–1.63 ^Hi^ 2.04; 1.24–3.40
ASDAS-CII	1.01; 0.83–1.24	1.34; 1.05–1.71	^3a^ 1.33; 1.05–1.70 ^3b^ 1.30; 0.99–1.69	^Lo^ 0.95; 0.54–1.68 ^Hi^ 1.60; 1.23–2.10
ASDAS-MI	0.92; 0.72–1.16	1.38; 1.04–1.85	^3a^ 1.36; 1.02–1.82 ^3b^ 1.50; 1.08–2.08	^Lo^ 1.19; 0.62–2.28 ^Hi^ 1.66; 1.02–2.72
ASAS-20	2.17; 1.77–2.66	1.92; 1.52–2.44	^3a^ 1.90; 1.50–2.42 ^3b^ 1.96; 1.53–2.52	^Lo^ 1.12; 0.999–1.25 ^Hi^ 2.62; 1.002–6.88
ASAS-40	1.93; 1.54–2.45	1.68; 1.30–2.19	^3a^ 1.67; 1.28–2.17 ^3b^ 1.72; 1.30–2.28	^Lo^ 1.31; 0.49–3.70 ^Hi^ 2.65; 1.63–4.40
ASAS-5/6	1.78; 1.37–2.31	1.57; 1.17–2.12	^3a^ 1.54; 1.14–2.08 ^3b^ 1.65; 1.20–2.27	^Lo^ 1.31; 0.81–2.11 ^Hi^ 2.28; 1.06–5.07
**12-month outcome**				
N ^1^	1091 to 1996	934 to 1614	795 to 1610	214 to 1576
BASDAI-50	1.10; 0.90–1.34	1.47; 1.16–1.87	^3a^ 1.47; 1.15–1.87 ^3b^ 1.50; 1.15–1.96	^Lo^ 1.13; 0.67–1.90 ^Hi^ 1.73; 1.28–2.34
ASDAS-ID	1.42; 1.15–1.75	1.60; 1.17–2.18	^3a^ 1.59; 1.69–2.18 ^3b^ 1.76; 1.25–2.50	^Lo^ 0.99; 0.53–1.80 ^Hi^ 2.06; 1.44–2.97
ASDAS-LDA	1.53; 1.28–1.82	1.54; 1.18–2.02	^3a^ 1.53; 1.17–2.01 ^3b^ 1.62; 1.21–2.19	^Lo^ 1.11; 0.63–1.96 ^Hi^ 1.80; 1.11–2.94
ASDAS-CII	0.85; 0.68–1.07	1.24; 0.94–1.64	^3a^ 1.24; 0.94–1.64 ^3b^ 1.31; 0.97–1.77	^Lo^ 0.97; 0.59–1.60 ^Hi^ 1.45; 0.89–2.37
ASDAS-MI	0.86; 0.66–1.10	1.28; 0.94–1.75	^3a^ 1.28; 0.94–1.75 ^3b^ 1.34; 0.95–1.91	^Lo^ 0.85; 0.47–1.53 ^Hi^ 1.56; 1.10–2.22
ASAS-20	1.88; 1.51–2.34	1.72; 1.31–2.26	^3a^ 1.70; 1.30–2.23 ^3b^ 1.72; 1.30–2.30	^Lo^ 1.37; 0.88–2.14 ^Hi^ 4.27; 1.13–17.8
ASAS-40	1.96; 1.55–2.50	1.74; 1.31–2.31	^3a^ 1.71; 1.29–2.28 ^3b^ 1.66; 1.23–2.24	^Lo^ 1.21; 0.76–1.92 ^Hi^ 21.7; 3.51–439
ASAS-5/6	1.56; 1.18–2.05	1.40; 1.02–1.94	^3a^ 1.37; 0.99–1.90 ^3b^ 1.38; 0.98–1.95	^Lo^ 1.06; 0.62–1.83 ^Hi^ 2.52; 0.69–9.20

^1^ N is the minimum and maximum number of participants in the subsequent models

Model 1– Crude association, unadjusted. All models present the odds ratio associated with being a current drinker, versus ex- and never-drinkers combined

Model 2– As per Model 1 but adjusted for age, sex, country, calendar year, quintiles of disease duration and baseline disease activity

Model 3a/3b– As per Model 2 but also adjusted for other lifestyle factors, denoted superscript 3a (Model 3a, adjusted for smoking) and 3b (Model 3b, adjusted for BMI categories)

Additional information, including the number of participants in each model, and a full description of Model 2, is shown in the supplementary data

Sensitivity analysis– As per Model 2 but additionally adjusted, in turn, for all other variables shown in Table 2. Results from individual models are shown in supplementary data. Here, the lowest and highest odds ratios are presented, denoted superscript Lo and Hi, respectively

### Sensitivity analyses

When the models describing the relationship between lifestyle factors and treatment outcomes were additionally adjusted for all remaining clinical and patient-reported measures no important changes were observed for the main effects under examination (Tables 4, 5 and 6; sensitivity analyses).

## Discussion

For the first time, we have provided a robust quantification of the influence of lifestyle factors on TNFi response in axSpA. Using data from 14 countries across Europe, we have shown that those who smoke experience a 20–25% decrease in the odds of achieving treatment response during the first year of treatment, and high BMI is associated with up to a 50% decrease in the odds of satisfying treatment response criteria. In contrast, patients who reported being current alcohol drinkers are more likely to meet treatment response criteria, compared to those who do not consume alcohol.

The analysis of data from the EuroSpA collaboration represents the largest attempt to date to quantify the effect of lifestyle factors on response to TNFi amongst patients with axSpA. However, as with any observational study there are limitations. It is possible that selective attrition and an absence of follow-up data for some patients has introduced a selection bias. However, it is hard to predict the direction of this potential bias. To determine treatment response, patients are required to attend clinic, and it may be that those who attend rheumatology departments regularly are better managed, and experience better outcomes. Also, it may be that patients who fail to respond to therapy are more likely to return to clinic, in which case we will have underestimated the prevalence of a treatment response. However, to affect the current results, one would have to argue that the relationship between lifestyle factors and treatment outcome also varies between patients who do / do not attend clinic. We consider this to be unlikely.

We have statistically adjusted for important potential confounders of the relationship between lifestyle factors and treatment response: age, sex, country, calendar year, disease duration and baseline disease activity. However, we did not construct full multivariable models, adjusting for every clinical variable that was available into a single model. Instead, our main results are adjusted for key variables (age, gender, country, calendar year, disease duration and disease activity) and, crucially, also for each other lifestyle factor. Some clinical characteristics (e.g. cardiovascular disease) may be consequences of the lifestyle factors under investigation, and therefore part of the mechanism underpinning the lifestyle factor / treatment response relationship. Thus, it would be inappropriate to adjust for cardiovascular disease. Also, it may be that smoking and/or obesity contribute to an elevated CRP which, being a component of the ASDAS, influences the treatment response measures. Similarly, in this situation adjusting for CRP would mask the true influence of smoking on treatment outcome.

However, for completeness, we conducted a series of sensitivity analyses, adjusting for all other clinical variables in turn. One must consider the nature of any potential confounding relationships. For example, how might HLA-B27 positivity influence smoking? It is hard to conceive of a simple causal association. However, it is plausible that any clinical factor that influences disease characteristics (e.g., disease severity, peripheral vs. axial disease, or extra-musculoskeletal manifestations) might be likely, indirectly, to influence lifestyle behaviour, and if also associated with bDMARD outcome might confound the association between treatment and treatment response. Comparison between models in the sensitivity analysis is difficult: not only does the number of participants vary, but often also the number of registries. For each association (e.g. baseline smoking and BASDAI-50 response at 3 months) there are an additional 21 models (i.e. adjusted in turn for all other variables). Thus, we caution against singling out individual odds ratio estimates, and one must be wary of multiple testing. It would have been informative to control for the potential confounding effect of socioeconomic status, educational attainment or health literacy, although this data was not available. However, socioeconomic status is associated with a number of the covariates that were examined (comorbidities, fatigue, patient and physical global assessment) and, while one cannot rule out residual confounding, across all models there is a consistency supporting our main findings– i.e. a modest decrease in the odds of satisfying treatment response criteria associated with smoking. A similarly consistent effect was observed with overweight and obesity, and a beneficial effect of alcohol consumption.

Lifestyle factors were documented in the period +/-30 days of baseline. This is arguably quite a strict window and may have led to exclusion of some patients. It’s possible also that there is some misclassification, where recent status does not accurately capture long-term lifestyle exposure– e.g. individuals who have recently given up smoking after decades of exposure. However, in clinic, current status is the most relevant exposure, and any such misclassification is unlikely to bias the results unless it occurs systematically differently among patients who do / do not meet treatment response criteria several months later. Even if data is taken from medical notes, smoking and alcohol consumption are most commonly based on self-report and there is, therefore, an inherent concern about validity. Most registries only collected data on current status and therefore all forms of alcohol use and smoking were classified as current use. Thus, in the current data it is not possible to distinguish between the casual drinker who has 1–2 glasses of wine per week, and the person who routinely drinks to excess. Similarly, it would have been interesting to examine how many non-smokers were ex- versus never-smokers (and likewise with alcohol consumption) although this data was seldom available, and also to examine changes in lifestyle factors over 12-month follow-up, but this is beyond the scope of the current study. However, it is unlikely that there will have been major lifestyle changes over a one-year period– for example: data from the Centers for Disease Control and Prevention in the USA suggest that fewer than one in ten cigarette smokers succeed in giving up smoking per year [23].

Although there was some variation between different response criteria, we consistently found that smoking was associated with a decrease in the odds of treatment response. The fact that there is consistency is unsurprising, and to a large extent reflects the overlap between the different response criteria. In contrast to many of the outcome measures, which capture improvement, ASDAS-ID is a measure of disease remission status. We cannot determine whether this is remission still on or off therapy. With only 12 months of follow-up it is likely to be the former, although the data is not available in the current analysis.

A recent systematic review found that smoking increases structural progression in a dose-dependent manner [24]. With respect to other disease markers, such as disease activity, pain and quality of life, the data suggest that patients who smoke have worse outcomes than non-smokers, although the overall evidence is poor. Glintborg et al. 2016 demonstrated that current and previous smokers had poorer treatment adherence than never smokers and were also less likely to achieve a BASDAI-50 treatment response following TNFi initiation [3]. Others have presented some evidence in support of this, although the results were not statistically significant [25]. Although smokers / non-smokers will differ in baseline characteristics (smokers have higher disease activity, etc.) our findings were robust to adjustment for baseline disease activity and many demographic, clinical and patient-reported disease measures. The results with smoking were also robust to statistical adjustment for alcohol consumption and BMI. Across the various outcomes adjustment resulted in a small increase in effect, suggesting that these other lifestyle factors only exerted a small confounding effect. Unfortunately, data on the quantity and/or duration of smoking (information on pack-years, for example) was available from only three registries. It would have been interesting to examine whether a dose-response relationship exists between smoking exposure and impact on treatment response, but this would have led to large increases in missing data, and many registries being unable to contribute to the analysis.

In contrast, we can provide clear evidence of an inverse dose-risk relationship between BMI and decreasing odds of treatment response– the greater the BMI, the lower the odds of achieving a positive treatment outcome. The relationship between obesity and disease activity has been shown previously [26], and others have reported a dose-risk association between obesity and ASAS-40 response at 12 months [27]. We have now replicated this finding in a considerably larger dataset– up to a 9-fold increase in sample size, depending on response criteria.

Each model comprised a slightly different number of participants, depending on the extent of missing data. Information on the precise number in each model is available in the supplementary data. Where data was missing it may have been individual-missing (i.e., a patient had no value for a particular variable), or missing by design (where a certain variable was not collected in a particular registry). Regarding the latter, even if the prevalence of adverse lifestyle factors smoking were to vary between countries (and this is likely) it does not necessarily follow that the influence of these factors on treatment response would also vary. To examine the effect of missing data we conducted a post hoc sensitivity analysis using multiple imputation to predict BASDAI-50 response at 12 months. The variables selected for the imputation model included registry (country), age at baseline, gender, education, disease duration, baseline disease measures, HLA-B27 status, presence/absence of comorbidities (psoriasis, uveitis, cardiovascular, diabetes, kidney disease, inflammatory bowel disease), treatment (TNFi, conventional synthetic DMARDs, NSAIDs), inflammation (CRP, ESR), baseline disease measures (Bath indices), lifestyle factors, and BASDAI50 response at 3 and 12-month follow-up. When missing values were imputed, while there was a slight attenuation of effect, current smoking was still associated with decrease in the odds of treatment response (OR: 0.87; 0.77–0.99). Similarly, being overweight (0.89; 0.79–0.998) or obese (0.77; 0.67–0.99) were associated with decreases in the likelihood of achieve BASDAI-50 response. The association with currently alcohol consumption was attenuated, and all but disappeared (1.05; 0.91–1.21). These results suggest that, while we cannot rule out the fact that bias was introduced due to missing data, the effect– at least in the case of smoking and BMI– has been small, and does not change the study conclusions.

Out findings suggest that a ‘more healthy’ lifestyle (non-smoking, normal BMI, etc.) should be associated with a greater probability of treatment response. In terms of practical implication, were 100 smokers commencing TNFi able to give up smoking, an additional ten would achieve BASDAI-50 at 12 months. However, this must be interpreted with some caution, as the current study examined differences in treatment outcome between smoking and non-smoking status at baseline, rather than the effect of smoking cessation per se. We would argue that most major confounders have been accounted for, but it is possible that residual confounding exists. This might change the effect size further (up or down, depending on the relationship between the confounder and the lifestyle factor, outcome, and other confounders) but our results give a clear indication of the likely effect size which will be valuable to patients and clinicians.

The observed effect of alcohol is intriguing and needs to be interpreted with caution. Similar to smoking data, we have no information about the quantity of alcohol consumption, and therefore modest drinkers and heavy drinkers are considered as one group. We also did not have data on the type of alcohol consumed– beers, wines, spirits, etc. It is known that low to moderate wine consumption is fairly reliable proxy for higher socioeconomic status, which is a universal positive prognostic marker for many disorders (not confined to rheumatology). It is interesting that alcohol consumption had little effect on the effect of smoking and/or BMI. The exception was with smoking, and ASAS major improvement criteria, although we hesitate to draw any conclusions from one comparison among many. It is important to note that the reference group was not homogeneous and included never-drinkers and ex-drinkers combined. Finally, there was also a high proportion of missing data– data were available on fewer than 4000 participants, versus 14,000 with smoking data– and, as discussed above, the observed association with alcohol was not replicated in the sensitivity analysis where missing data was imputed.

In summary, we have shown among patients with axSpA commencing TNFi that current smoking and high BMI is associated with a 25–50% decrease in the odds of satisfying treatment response criteria at 12 months. These findings highlight the importance of capturing data on lifestyle factors as part of routine clinical care. Failure to do so means omits important background information in these patients. The current results are based on observational data, and without randomised trial evidence we cannot conclude that successful change in lifestyle will necessarily yield improvements in treatment response. However, such a trial is unlikely to be undertaken. It is known that lifestyle factors may differ between patients who are / are not being escalated to biologic therapy. The current findings show that even after adjusting for baseline disease activity and other potential confounders, such lifestyle factors are markers of treatment response. Although all participants in the current analysis were commencing TNFi, our results do not imply that the associations are unique to TNFi and we might expect similar associations among patients commencing other advanced therapies. It may be timely, at the point a patient is commencing any new pharmacological therapy, to think about potential lifestyle interventions. Therefore, physicians should consider referring relevant patients to specialised weight management and smoking cessation services. Indeed, such interventions may offer the potential to enhance treatment outcomes in axSpA. Furthermore, over and above the effect of the medication itself, even small marginal gains in disease activity, function, and quality of life may reduce the likelihood of treatment failure.

## Electronic supplementary material

Below is the link to the electronic supplementary material.

Supplementary Material 1

## Data Availability

Data is not available to external researchers would require individual permission from each contributing registry. Queries about accessing the data contained in this manuscript should be addressed to the corresponding author.

## Footnotes

1 3-month follow-up was defined as any visit between 45 and 135 days after commencing bDMARD therapy. 12-month follow-up was defined as any visit between 271 and 545 days after commencing bDMARD therapy. If there was more than one data collection point within either interval, priority was given to the data collected closest to 90 days and 365 days, respectively.

## Declarations

In Denmark (DANBIO) ethical approval not required for registry studies [komitelovens § 14, stk. 2, www.nvk.dk], and in Iceland (ICEBIO) it is possible to receive selected pre-defined data from registers for use in epidemiological studies with prior approval from the National Bioethics Committee and Data Protective Authorities without informed consent from the individual patient. In these cases, no personal identifiers are delivered to the researchers. For all other countries, informed consent to participate was obtained from all of the participants, prior to inclusion in the individual registries.

## Abbreviations

ASAS: Assessment of Spondyloarthritis International Society
ASAS-20: ASAS 20 response criteria
ASAS-40: ASAS 40 response criteria
ASAS-5/6: ASAS 5/6 response criteria
ASDAS: Axial Spondyloarthritis Disease Activity Score
ASDAS-CII: ASDAS Clinically Important Improvement
ASDAS-ID: ASDAS Inactive Disease
ASDAS-LDA: ASDAS Low Disease Activity
ASDAS-MI: ASDAS Major Improvement
axSpA: Axial Spondyloarthritis
BASDAI: Bath Ankylosing Disease Activity Index
BASDAI-50: BASDAI 50 response criteria
BASFI: Bath Ankylosing Functional Index
BASMI: Bath Ankylosing Metrology Index
BMI: Body mass index
CRP: C-Reactive Protein
DMARDs: Disease Modifying Anti-Rheumatic Drugs
bDMARDs: Biologic DMARDs
csDMARDs: Conventional synthetic DMARDs
ESR: Erythrocyte Sedimentation Rate
EuroSpA: EuroSpA Research Collaboration Network (RCN)
HAQ: Health Assessment Questionnaire
HLA-B27: Human Leucocyte Antigen B27
Ln: Natural Logarithm
NSAIDs: Non-steroidal anti-inflammatory drugs
OR: Odds ratio
TNFi: Tumour Necrosis Factor α inhibitor
95%CI: 95% Confidence interval
