# Supplementary material for: The relationship between lifestyle factors and outcome of treatment with TNFα inhibitors in axial spondyloarthritis – results from 14 European countries
Source: BMC Rheumatol. 2025 Jul 11;9:88. doi: 10.1186/s41927-025-00529-4 (PMC12247191; doi:10.1186/s41927-025-00529-4)
Supplement: Supplementary file 1 — Supplementary Material 1 [file 41927_2025_529_MOESM1_ESM.pdf]

Jones et al. The relationship between lifestyle factors and outcome of treatment with TNFα inhibitors in axial spondyloarthritis – results from 14 European countries

SUPPLEMENTARY DATA – INDEX

1. Association between baseline smoking and 3-month:
    a. BASDAI-50 3
    b. ASDAS-Inactive disease 4
    c. ASDAS-LDA (low disease activity) 5
    d. ASDAS-CII (clinically important improvement) 6
    e. ASDAS-MI (major improvement) 7
    f. ASAS-20 8
    g. ASAS-40 9
    h. ASAS-5/6 10

2. Association between baseline smoking and 12-month:
    a. BASDAI-50 11
    b. ASDAS-Inactive disease 12
    c. ASDAS-LDA (low disease activity) 13
    d. ASDAS-CII (clinically important improvement) 14
    e. ASDAS-MI (major improvement) 15
    f. ASAS-20 16
    g. ASAS-40 17
    h. ASAS-5/6 18

3. Association between baseline body mass index and 3-month:
    a. BASDAI-50 19
    b. ASDAS-Inactive disease 20
    c. ASDAS-LDA (low disease activity) 21
    d. ASDAS-CII (clinically important improvement) 22
    e. ASDAS-MI (major improvement) 23
    f. ASAS-20 24
    g. ASAS-40 25
    h. ASAS-5/6 26

4. Association between baseline body mass index and 12-month:
    a. BASDAI-50 27
    b. ASDAS-Inactive disease 28
    c. ASDAS-LDA (low disease activity) 29
    d. ASDAS-CII (clinically important improvement) 30
    e. ASDAS-MI (major improvement) 31
    f. ASAS-20 32
    g. ASAS-40 33
    h. ASAS-5/6 34

5. Association between baseline alcohol use and 3-month:
    a. BASDAI-50 35
    b. ASDAS-Inactive disease 36
    c. ASDAS-LDA (low disease activity) 37
    d. ASDAS-CII (clinically important improvement) 38
    e. ASDAS-MI (major improvement) 39
    f. ASAS-20 40
    g. ASAS-40 41
    h. ASAS-5/6 42

6. Association between baseline alcohol use and 12-month:
   a. BASDAI-50 43
   b. ASDAS-Inactive disease 44
   c. ASDAS-LDA (low disease activity) 45
   d. ASDAS-CII (clinically important improvement) 46
   e. ASDAS-MI (major improvement) 47
   f. ASAS-20 48
   g. ASAS-40 49
   h. ASAS-5/6 50

7. CRediT (Contributor Roles Taxonomy) author statement 51

Supplementary data: Association between SMOKING at baseline and BASDAI-50 at 3 months

Model 1 ---------------------------- Crude (unadjusted) association

Exposure	Smoking
Outcome	BASDAI-50
Timepoint	3 months
N (countries)	13
N (patients)	6293

		BASDAI-50	
		No	Yes
Smoking	No	2555	1883
	Yes	1101	754

OR	95%CI-Lo	95%CI-Hi
0.93	0.83	1.04

Model 2 ---------------------------- Model 1, adjusted for: age, gender, country, calendar year, disease duration and disease activity

N (countries)	13
N (patients)	6284

Reference country = Switzerland (CH)
Blank line = No data for this analysis

	OR	95%CI-Lo	95%CI-Hi
Smoking = Yes	0.83	0.73	0.93
Age	0.97	0.97	0.98
Male	1.67	1.50	1.86
BASDAI	1.00	1.00	1.00
DiseaseDuration_2	0.85	0.72	1.00
DiseaseDuration_3	1.07	0.93	1.24
DiseaseDuration_4	0.98	0.83	1.15
DiseaseDuration_5	1.11	0.89	1.39
CalendarYear	1.00	0.98	1.02
Czech Republic (CZ)	1.68	0.95	2.98
Denmark (DK)	0.69	0.52	0.93
Spain (ES)	0.41	0.11	1.23
Iceland (IS)	0.94	0.33	2.67
Italy (IT)	0.95	0.64	1.40
Netherlands (NL)			
Norway (NO)	1.18	0.87	1.60
Portugal (PT)	1.00	0.72	1.38
Romania (RO)	6.49	3.62	12.14
Finland (SF)	1.03	0.67	1.58
Slovenia (SI)	1.37	0.98	1.94
Turkey (TR)	2.52	1.82	3.52
United Kingdom (UK)	0.60	0.41	0.89

Model 3a/3b ---------------------- Model 2 additionally adjusted, in turn, for Alcohol and BMI

Individual models. Result = OR (Smoking) from each model

		OR (smoking)	95%CI-Lo	95%CI-Hi	OR↑↓	N	N↓
(3a)	Alcohol	0.76	0.61	0.95	0.062	1920	4364 (69%)
(3b)	BMI	0.71	0.60	0.84	0.118	3263	3021 (48%)

Sensitivity analysis -------------- Model 2 additionally adjusted, in turn, for other variables shown

Individual models. Result = OR (Smoking) from each model

	OR (smoking)	95%CI-Lo	95%CI-Hi	OR↑↓	N	N↓
Yrs since symptom onset	0.80	0.69	0.92	0.027	4097	2187 (35%)
HLA-B27 status	0.84	0.72	0.98	0.018	3671	2613 (42%)
ESR	0.71	0.59	0.87	0.111	2681	3603 (57%)
CRP	0.75	0.66	0.85	0.079	5482	802 (13%)
mNY criteria	0.73	0.60	0.89	0.093	2388	3896 (62%)
ASAS criteria	0.79	0.67	0.94	0.032	2898	3386 (54%)
IBD	0.77	0.65	0.91	0.055	3079	3205 (51%)
Uveitis	0.79	0.67	0.94	0.031	3043	3241 (52%)
Psoriasis	0.78	0.65	0.92	0.048	3067	3217 (51%)
Comorbidity cardiovascular	0.75	0.57	1.02	0.079	2338	3946 (63%)
Comorbidity kidney	0.77	0.62	0.95	0.054	2246	4038 (64%)
Comorbidity diabetes	0.75	0.61	0.92	0.073	2361	3923 (62%)
BASFI	0.85	0.74	0.97	0.024	4992	1292 (21%)
BASMI (3pt)	0.82	0.64	1.03	0.009	1556	4728 (75%)
Patient global	0.82	0.73	0.93	0.001	5799	485 (8%)
Physician global	0.85	0.74	0.96	0.021	4466	1818 (29%)
Pain (VAS)	0.82	0.73	0.93	0.002	5547	737 (12%)
Fatigue	0.72	0.62	0.85	0.102	6035	249 (4%)
HAQ	0.85	0.73	0.99	0.026	3879	2405 (38%)
csDMARD	0.64	0.52	0.78	0.189	2330	3954 (63%)
NSAID	0.73	0.58	0.91	0.097	1732	4552 (72%)

(red) = Highest OR value
(blue) = Lowest OR value

Supplementary data: Association between SMOKING at baseline and ASDAS-Inactive at 3 months

Model 1 ---------------------------- Crude (unadjusted) association

Exposure	Smoking
Outcome	ASDAS-Inactive
Timepoint	3 months
N (countries)	12
N (patients)	6511

		ASDAS-Inactive	
		No	Yes
Smoking	No	3461	1106
	Yes	1546	398

OR	95%CI-Lo	95%CI-Hi
0.81	0.71	0.92

Model 2 ---------------------------- Model 1, adjusted for: age, gender, country, calendar year, disease duration and disease activity

N (countries) 12
N (patients) 5201

Reference country = Switzerland (CH)
Blank line = No data for this analysis

	OR	95%CI-Lo	95%CI-Hi
Smoking = Yes	0.72	0.62	0.84
Age	0.96	0.96	0.97
Male	1.82	1.57	2.10
BASDAI	0.97	0.97	0.97
DiseaseDuration_2	0.92	0.74	1.14
DiseaseDuration_3	1.28	1.07	1.55
DiseaseDuration_4	0.93	0.74	1.16
DiseaseDuration_5	1.29	0.97	1.71
CalendarYear	1.03	1.00	1.06
Czech Republic (CZ)	2.94	1.52	5.67
Denmark (DK)	0.77	0.52	1.15
Spain (ES)			
Iceland (IS)	0.75	0.11	3.25
Italy (IT)	0.66	0.32	1.35
Netherlands (NL)			
Norway (NO)	1.29	0.86	1.94
Portugal (PT)	1.06	0.69	1.65
Romania (RO)	2.24	1.21	4.12
Finland (SF)	1.62	0.85	3.09
Slovenia (SI)	0.69	0.34	1.35
Turkey (TR)	0.69	0.45	1.06
United Kingdom (UK)	0.54	0.30	0.95

Model 3a/3b ---------------------- Model 2 additionally adjusted, in turn, for Alcohol and BMI

Individual models. Result = OR (Smoking) from each model

		OR (smoking)	95%CI-Lo	95%CI-Hi	OR↑↓	N	N↓
(3a)	Alcohol	0.59	0.44	0.80	0.129	1707	3494 (67%)
(3b)	BMI	0.59	0.46	0.74	0.136	2469	2732 (53%)

Sensitivity analysis -------------- Model 2 additionally adjusted, in turn, for other variables shown

Individual models. Result = OR (Smoking) from each model

	OR (smoking)	95%CI-Lo	95%CI-Hi	OR↑↓	N	N↓
Yrs since symptom onset	0.72	0.60	0.87	0.003	3753	1448 (28%)
HLA-B27 status	0.70	0.57	0.86	0.024	2988	2213 (43%)
ESR	0.70	0.54	0.91	0.020	1986	3215 (62%)
CRP	0.69	0.58	0.81	0.034	4728	473 (9%)
mNY criteria	0.75	0.56	0.98	0.024	1864	3337 (64%)
ASAS criteria	0.63	0.49	0.80	0.094	2350	2851 (55%)
IBD	0.57	0.45	0.73	0.149	2418	2783 (54%)
Uveitis	0.61	0.47	0.77	0.113	2385	2816 (54%)
Psoriasis	0.60	0.47	0.76	0.126	2417	2784 (54%)
Comorbidity cardiovascular	0.77	0.58	1.02	0.048	1769	3432 (66%)
Comorbidity kidney	0.79	0.59	1.05	0.069	1680	3521 (68%)
Comorbidity diabetes	0.79	0.60	1.05	0.072	1787	3414 (66%)
BASFI	0.70	0.58	0.84	0.021	4064	1137 (22%)
BASMI (3pt)	0.71	0.52	0.96	0.014	1424	3777 (73%)
Patient global	0.72	0.62	0.85	0.002	5125	76 (1%)
Physician global	0.72	0.61	0.85	0.001	4466	735 (14%)
Pain (VAS)	0.72	0.61	0.85	0.004	4617	584 (11%)
Fatigue	0.72	0.62	0.85	0.002	5131	70 (1%)
HAQ	0.80	0.66	0.97	0.080	3179	2022 (39%)
csDMARD	0.55	0.41	0.73	0.168	1859	3342 (64%)
NSAID	0.77	0.58	1.02	0.049	1556	3645 (70%)

= Highest OR value (red)
= Lowest OR value (blue)

Supplementary data: Association between SMOKING at baseline and ASDAS-LDA at 3 months

Model 1 ---------------------------- Crude (unadjusted) association

Exposure	Smoking
Outcome	ASDAS-LDA
Timepoint	3 months
N (countries)	12
N (patients)	6511

		ASDAS-LDA	
		No	Yes
Smoking	No	2172	2395
	Yes	1037	907

OR	95%CI-Lo	95%CI-Hi
0.79	0.71	0.88

Model 2 ---------------------------- Model 1, adjusted for: age, gender, country, calendar year, disease duration and disease activity

N (countries)	12
N (patients)	5201

Reference country = Switzerland (CH)
Blank line = No data for this analysis

	OR	95%CI-Lo	95%CI-Hi
Smoking = Yes	0.74	0.65	0.85
Age	0.97	0.97	0.98
Male	1.66	1.46	1.88
BASDAI	0.97	0.96	0.97
DiseaseDuration_2	1.00	0.83	1.19
DiseaseDuration_3	1.14	0.96	1.35
DiseaseDuration_4	0.96	0.80	1.16
DiseaseDuration_5	1.06	0.82	1.38
CalendarYear	1.01	0.99	1.04
Czech Republic (CZ)	1.24	0.66	2.32
Denmark (DK)	0.56	0.39	0.79
Spain (ES)			
Iceland (IS)	0.28	0.06	1.10
Italy (IT)	0.75	0.40	1.40
Netherlands (NL)			
Norway (NO)	1.08	0.75	1.55
Portugal (PT)	0.76	0.52	1.11
Romania (RO)	2.99	1.68	5.41
Finland (SF)	1.24	0.66	2.39
Slovenia (SI)	0.61	0.37	1.01
Turkey (TR)	0.70	0.48	1.03
United Kingdom (UK)	0.54	0.34	0.84

Model 3a/3b ----------------------- Model 2 additionally adjusted, in turn, for Alcohol and BMI

Individual models. Result = OR (Smoking) from each model

		OR (smoking)	95%CI-Lo	95%CI-Hi	OR↑↓	N	N↓
(3a)	Alcohol	0.67	0.53	0.85	0.070	1707	3494 (67%)
(3b)	BMI	0.60	0.49	0.73	0.148	2469	2732 (53%)

Sensitivity analysis --------------- Model 2 additionally adjusted, in turn, for other variables shown

Individual models. Result = OR (Smoking) from each model

	OR (smoking)	95%CI-Lo	95%CI-Hi	OR↑↓	N	N↓
Yrs since symptom onset	0.75	0.65	0.88	0.009	3753	1448 (28%)
HLA-B27 status	0.72	0.60	0.86	0.024	2988	2213 (43%)
ESR	0.74	0.59	0.94	0.001	1986	3215 (62%)
CRP	0.73	0.63	0.84	0.014	4728	473 (9%)
mNY criteria	0.70	0.56	0.87	0.044	1864	3337 (64%)
ASAS criteria	0.69	0.56	0.83	0.059	2350	2851 (55%)
IBD	0.67	0.55	0.81	0.079	2418	2783 (54%)
Uveitis	0.68	0.56	0.83	0.061	2385	2816 (54%)
Psoriasis	0.64	0.53	0.78	0.100	2417	2784 (54%)
Comorbidity cardiovascular	0.67	0.52	0.86	0.074	1769	3432 (66%)
Comorbidity kidney	0.66	0.51	0.86	0.081	1680	3521 (68%)
Comorbidity diabetes	0.67	0.52	0.86	0.078	1787	3414 (66%)
BASFI	0.80	0.69	0.93	0.054	4064	1137 (22%)
BASMI (3pt)	0.81	0.63	1.04	0.063	1424	3777 (73%)
Patient global	0.76	0.66	0.87	0.016	5125	76 (1%)
Physician global	0.78	0.68	0.90	0.038	4466	735 (14%)
Pain (VAS)	0.72	0.63	0.83	0.020	4617	584 (11%)
Fatigue	0.74	0.65	0.85	0.003	5131	70 (1%)
HAQ	0.75	0.63	0.89	0.004	3179	2022 (39%)
csDMARD	0.56	0.44	0.71	0.182	1859	3342 (64%)
NSAID	0.75	0.58	0.96	0.004	1556	3645 (70%)

= Highest OR value
= Lowest OR value

Supplementary data: Association between SMOKING at baseline and ASDAS-CII at 3 months

Model 1 ---------------------------- Crude (unadjusted) association

Exposure	Smoking
Outcome	ASDAS-CII
Timepoint	3 months
N (countries)	12
N (patients)	4755

		ASDAS-CII No	ASDAS-CII Yes
Smoking	No	1696	1601
	Yes	711	747

OR	95%CI-Lo	95%CI-Hi
1.11	0.98	1.26

Model 2 ---------------------------- Model 1, adjusted for: age, gender, country, calendar year, disease duration and disease activity

N (countries)	12
N (patients)	4739

Reference country = Switzerland (CH)
Blank line = No data for this analysis

	OR	95%CI-Lo	95%CI-Hi
Smoking = Yes	0.96	0.84	1.10
Age	0.98	0.98	0.99
Male	1.85	1.63	2.09
BASDAI	1.02	1.02	1.03
DiseaseDuration_2	0.77	0.64	0.92
DiseaseDuration_3	0.91	0.77	1.08
DiseaseDuration_4	0.96	0.80	1.16
DiseaseDuration_5	0.99	0.76	1.29
CalendarYear	0.99	0.97	1.01
Czech Republic (CZ)	3.46	1.67	7.77
Denmark (DK)	0.58	0.41	0.82
Spain (ES)			
Iceland (IS)	0.36	0.07	1.72
Italy (IT)	0.36	0.18	0.70
Netherlands (NL)			
Norway (NO)	0.95	0.67	1.35
Portugal (PT)	0.90	0.62	1.30
Romania (RO)	6.07	2.95	13.60
Finland (SF)	0.83	0.41	1.63
Slovenia (SI)	0.55	0.33	0.91
Turkey (TR)	2.10	1.45	3.06
United Kingdom (UK)	0.48	0.31	0.74

Model 3a/3b ---------------------- Model 2 additionally adjusted, in turn, for Alcohol and BMI

Individual models. Result = OR (Smoking) from each model

		OR (smoking)	95%CI-Lo	95%CI-Hi	OR↑↓	N	N↓
(3a)	Alcohol	0.91	0.72	1.15	0.047	1549	3190 (67%)
(3b)	BMI	0.82	0.67	0.99	0.144	2262	2477 (52%)

Sensitivity analysis --------------- Model 2 additionally adjusted, in turn, for other variables shown

Individual models. Result = OR (Smoking) from each model

	OR (smoking)	95%CI-Lo	95%CI-Hi	OR↑↓	N	N↓
Yrs since symptom onset	0.98	0.84	1.15	0.021	3490	1249 (26%)
HLA-B27 status	0.97	0.81	1.16	0.010	2723	2016 (43%)
ESR	0.92	0.72	1.18	0.041	1930	2809 (59%)
CRP	0.85	0.73	0.98	0.113	4654	85 (2%)
mNY criteria	0.90	0.72	1.13	0.059	1701	3038 (64%)
ASAS criteria	0.89	0.73	1.08	0.072	2149	2590 (55%)
IBD	0.89	0.73	1.08	0.073	2201	2538 (54%)
Uveitis	0.92	0.75	1.12	0.043	2177	2562 (54%)
Psoriasis	0.91	0.74	1.10	0.055	2202	2537 (54%)
Comorbidity cardiovascular	1.01	0.78	1.30	0.047	1615	3124 (66%)
Comorbidity kidney	1.05	0.81	1.36	0.086	1532	3207 (68%)
Comorbidity diabetes	1.05	0.81	1.35	0.085	1629	3110 (66%)
BASFI	0.95	0.82	1.11	0.008	3706	1033 (22%)
BASMI (3pt)	0.86	0.68	1.10	0.096	1353	3386 (71%)
Patient global	0.94	0.82	1.08	0.019	4728	11 (0%)
Physician global	0.93	0.80	1.07	0.035	4137	602 (13%)
Pain (VAS)	0.92	0.80	1.06	0.038	4207	532 (11%)
Fatigue	0.96	0.84	1.10	0.002	4679	60 (1%)
HAQ	0.97	0.82	1.14	0.005	2918	1821 (38%)
csDMARD	0.80	0.63	1.01	0.165	1706	3033 (64%)
NSAID	0.99	0.77	1.27	0.030	1452	3287 (69%)

(red) = Highest OR value
(blue) = Lowest OR value

Supplementary data: Association between SMOKING at baseline and ASDAS-MI at 3 months

Model 1 ---------------------------- Crude (unadjusted) association

Exposure	Smoking
Outcome	ASDAS-MI
Timepoint	3 months
N (countries)	12
N (patients)	4755

		ASDAS-MI No	ASDAS-MI Yes
Smoking	No	2474	823
	Yes	1074	384

OR	95%CI-Lo	95%CI-Hi
1.07	0.93	1.24

Model 2 ---------------------------- Model 1, adjusted for: age, gender, country, calendar year, disease duration and disease activity

N (countries)	12
N (patients)	4739

Reference country = Switzerland (CH)
Blank line = No data for this analysis

	OR	95%CI-Lo	95%CI-Hi
Smoking = Yes	0.92	0.79	1.07
Age	0.97	0.97	0.98
Male	2.11	1.82	2.45
BASDAI	1.03	1.03	1.03
DiseaseDuration_2	0.82	0.66	1.02
DiseaseDuration_3	1.02	0.84	1.23
DiseaseDuration_4	0.99	0.79	1.23
DiseaseDuration_5	0.90	0.64	1.24
CalendarYear	0.97	0.94	0.99
Czech Republic (CZ)	3.41	1.78	6.63
Denmark (DK)	0.71	0.48	1.06
Spain (ES)			
Iceland (IS)	1.09	0.20	5.38
Italy (IT)	0.89	0.38	1.96
Netherlands (NL)			
Norway (NO)	0.83	0.55	1.25
Portugal (PT)	1.38	0.91	2.13
Romania (RO)	7.98	4.22	15.52
Finland (SF)	0.81	0.32	1.87
Slovenia (SI)	0.72	0.40	1.29
Turkey (TR)	2.31	1.51	3.60
United Kingdom (UK)	0.56	0.33	0.95

Model 3a/3b ---------------------- Model 2 additionally adjusted, in turn, for Alcohol and BMI

Individual models. Result = OR (Smoking) from each model

		OR (smoking)	95%CI-Lo	95%CI-Hi	OR↑↓	N	N↓
(3a)	Alcohol	1.01	0.77	1.32	0.097	1549	3190 (67%)
(3b)	BMI	0.83	0.66	1.04	0.085	2262	2477 (52%)

Sensitivity analysis -------------- Model 2 additionally adjusted, in turn, for other variables shown

Individual models. Result = OR (Smoking) from each model

	OR (smoking)	95%CI-Lo	95%CI-Hi	OR↑↓	N	N↓
Yrs since symptom onset	0.91	0.77	1.09	0.002	3490	1249 (26%)
HLA-B27 status	0.93	0.75	1.14	0.014	2723	2016 (43%)
ESR	0.88	0.66	1.16	0.038	1930	2809 (59%)
CRP	0.79	0.66	0.94	0.128	4654	85 (2%)
mNY criteria	0.87	0.67	1.13	0.047	1701	3038 (64%)
ASAS criteria	0.87	0.69	1.10	0.042	2149	2590 (55%)
IBD	0.91	0.72	1.14	0.005	2201	2538 (54%)
Uveitis	0.90	0.72	1.13	0.014	2177	2562 (54%)
Psoriasis	0.87	0.69	1.10	0.044	2202	2537 (54%)
Comorbidity cardiovascular	0.94	0.70	1.26	0.024	1615	3124 (66%)
Comorbidity kidney	0.93	0.69	1.26	0.016	1532	3207 (68%)
Comorbidity diabetes	0.96	0.72	1.29	0.048	1629	3110 (66%)
BASFI	0.93	0.78	1.10	0.015	3706	1033 (22%)
BASMI (3pt)	0.90	0.68	1.19	0.016	1353	3386 (71%)
Patient global	0.89	0.76	1.04	0.023	4728	11 (0%)
Physician global	0.90	0.76	1.06	0.018	4137	602 (13%)
Pain (VAS)	0.86	0.73	1.02	0.054	4207	532 (11%)
Fatigue	0.92	0.78	1.07	0.001	4679	60 (1%)
HAQ	0.94	0.77	1.14	0.020	2918	1821 (38%)
csDMARD	0.60	0.46	0.80	0.312	1706	3033 (64%)
NSAID	0.94	0.71	1.23	0.020	1452	3287 (69%)

= Highest OR value
= Lowest OR value

Supplementary data: Association between SMOKING at baseline and ASAS-20 at 3 months

Model 1 ---------------------------- Crude (unadjusted) association

Exposure	Smoking
Outcome	ASAS-20
Timepoint	3 months
N (countries)	11
N (patients)	5056

		ASAS-20 No	ASAS-20 Yes
Smoking	No	2124	1324
	Yes	984	624

OR	95%CI-Lo	95%CI-Hi
1.02	0.90	1.15

Model 2 ---------------------------- Model 1, adjusted for: age, gender, country, calendar year, disease duration and disease activity

N (countries) 9
N (patients) 4618

Reference country = Switzerland (CH)
Blank line = No data for this analysis

Rounding to 2dp. Actual value = non-zero.

	OR	95%CI-Lo	95%CI-Hi
Smoking = Yes	0.78	0.68	0.90
Age	0.98	0.98	0.99
Male	1.40	1.22	1.60
BASDAI	1.01	1.01	1.02
DiseaseDuration_2	0.84	0.69	1.01
DiseaseDuration_3	0.94	0.79	1.12
DiseaseDuration_4	0.86	0.70	1.05
DiseaseDuration_5	0.76	0.53	1.09
CalendarYear	1.01	0.98	1.04
Czech Republic (CZ)	2.01	1.06	3.98
Denmark (DK)	0.63	0.47	0.85
Spain (ES)			
Iceland (IS)	0.65	0.22	1.97
Italy (IT)	0.00	0.00	37.09
Netherlands (NL)			
Norway (NO)			
Portugal (PT)			
Romania (RO)			
Finland (SF)	0.57	0.36	0.92
Slovenia (SI)	0.30	0.20	0.46
Turkey (TR)	1.86	1.33	2.62
United Kingdom (UK)	0.59	0.40	0.87

Model 3a/3b ---------------------- Model 2 additionally adjusted, in turn, for Alcohol and BMI

Individual models. Result = OR (Smoking) from each model

		OR (smoking)	95%CI-Lo	95%CI-Hi	OR↑↓	N	N↓
(3a)	Alcohol	0.75	0.59	0.96	0.032	1539	3079 (67%)
(3b)	BMI	0.70	0.58	0.84	0.087	2546	2072 (45%)

Sensitivity analysis -------------- Model 2 additionally adjusted, in turn, for other variables shown

Individual models. Result = OR (Smoking) from each model

	OR (smoking)	95%CI-Lo	95%CI-Hi	OR↑↓	N	N↓
Yrs since symptom onset	0.79	0.67	0.93	0.006	3334	1284 (28%)
HLA-B27 status	0.77	0.62	0.95	0.016	2347	2271 (49%)
ESR	0.47	0.30	0.72	0.316	1216	3402 (74%)
CRP	0.75	0.64	0.87	0.034	4068	550 (12%)
mNY criteria	0.70	0.55	0.88	0.087	1660	2958 (64%)
ASAS criteria	0.77	0.63	0.93	0.019	2193	2425 (53%)
IBD	0.76	0.62	0.92	0.026	2300	2318 (50%)
Uveitis	0.76	0.62	0.93	0.024	2262	2356 (51%)
Psoriasis	0.73	0.60	0.89	0.056	2305	2313 (50%)
Comorbidity cardiovascular	0.50	0.35	0.72	0.282	1202	3416 (74%)
Comorbidity kidney	0.56	0.38	0.83	0.223	1101	3517 (76%)
Comorbidity diabetes	0.57	0.40	0.81	0.213	1221	3397 (74%)
BASFI	0.80	0.69	0.92	0.016	4214	404 (9%)
BASMI (3pt)	0.80	0.63	1.02	0.015	1391	3227 (70%)
Patient global	0.77	0.67	0.89	0.011	4495	123 (3%)
Physician global	0.78	0.67	0.90	0.005	4070	548 (12%)
Pain (VAS)	0.76	0.66	0.88	0.023	4373	245 (5%)
Fatigue	0.79	0.68	0.91	0.002	4526	92 (2%)
HAQ	0.79	0.65	0.94	0.001	2805	1813 (39%)
csDMARD	0.58	0.45	0.76	0.202	1571	3047 (66%)
NSAID	0.69	0.52	0.91	0.100	1268	3350 (73%)

(red) = Highest OR value
(blue) = Lowest OR value

Supplementary data: Association between SMOKING at baseline and ASAS-40 at 3 months

Model 1 ---------------------------- Crude (unadjusted) association

Exposure	Smoking
Outcome	ASAS-40
Timepoint	3 months
N (countries)	11
N (patients)	5735

		ASAS-40 No	ASAS-40 Yes
Smoking	No	3087	860
	Yes	1383	405

OR	95%CI-Lo	95%CI-Hi
1.05	0.92	1.20

Model 2 ---------------------------- Model 1, adjusted for: age, gender, country, calendar year, disease duration and disease activity

N (countries)	8
N (patients)	5110

Reference country = Switzerland (CH)
Blank line = No data for this analysis

	OR	95%CI-Lo	95%CI-Hi
Smoking = Yes	0.79	0.68	0.91
Age	0.98	0.97	0.98
Male	1.49	1.29	1.72
BASDAI	1.02	1.01	1.02
DiseaseDuration_2	0.90	0.74	1.09
DiseaseDuration_3	0.96	0.80	1.16
DiseaseDuration_4	0.80	0.64	0.99
DiseaseDuration_5	0.71	0.47	1.05
CalendarYear	1.03	1.00	1.06
Czech Republic (CZ)	1.21	0.67	2.19
Denmark (DK)	0.54	0.40	0.74
Spain (ES)			
Iceland (IS)	0.84	0.27	2.55
Italy (IT)			
Netherlands (NL)			
Norway (NO)			
Portugal (PT)			
Romania (RO)			
Finland (SF)	0.38	0.22	0.64
Slovenia (SI)	0.20	0.12	0.31
Turkey (TR)	1.29	0.92	1.84
United Kingdom (UK)	0.55	0.36	0.82

Model 3a/3b ---------------------- Model 2 additionally adjusted, in turn, for Alcohol and BMI

Individual models. Result = OR (Smoking) from each model

		OR (smoking)	95%CI-Lo	95%CI-Hi	OR↑↓	N	N↓
(3a)	Alcohol	0.67	0.51	0.88	0.114	1666	3444 (67%)
(3b)	BMI	0.67	0.54	0.82	0.120	2758	2352 (46%)

Sensitivity analysis -------------- Model 2 additionally adjusted, in turn, for other variables shown

Individual models. Result = OR (Smoking) from each model

	OR (smoking)	95%CI-Lo	95%CI-Hi	OR↑↓	N	N↓
Yrs since symptom onset	0.81	0.69	0.96	0.025	3560	1550 (30%)
HLA-B27 status	0.73	0.58	0.91	0.055	2725	2385 (47%)
ESR	0.41	0.25	0.66	0.371	1630	3480 (68%)
CRP	0.75	0.64	0.88	0.035	4485	625 (12%)
mNY criteria	0.69	0.53	0.89	0.094	1871	3239 (63%)
ASAS criteria	0.73	0.59	0.90	0.056	2396	2714 (53%)
IBD	0.68	0.55	0.84	0.104	2531	2579 (50%)
Uveitis	0.72	0.58	0.89	0.068	2489	2621 (51%)
Psoriasis	0.69	0.56	0.86	0.093	2531	2579 (50%)
Comorbidity cardiovascular	0.53	0.35	0.77	0.260	1541	3569 (70%)
Comorbidity kidney	0.52	0.27	1.00	0.263	1443	3667 (72%)
Comorbidity diabetes	0.55	0.37	0.80	0.239	1563	3547 (69%)
BASFI	0.80	0.68	0.93	0.011	4474	636 (12%)
BASMI (3pt)	0.85	0.66	1.10	0.069	1453	3657 (72%)
Patient global	0.77	0.66	0.90	0.012	4895	215 (4%)
Physician global	0.79	0.67	0.92	0.002	4382	728 (14%)
Pain (VAS)	0.76	0.66	0.89	0.022	4725	385 (8%)
Fatigue	0.79	0.68	0.92	0.004	4976	134 (3%)
HAQ	0.80	0.66	0.96	0.011	3121	1989 (39%)
csDMARD	0.55	0.41	0.73	0.236	1796	3314 (65%)
NSAID	0.64	0.48	0.86	0.142	1413	3697 (72%)

= Highest OR value (red)
= Lowest OR value (blue)

Supplementary data: Association between SMOKING at baseline and ASAS-5of6 at 3 months

Model 1 ---------------------------- Crude (unadjusted) association

Exposure	Smoking
Outcome	ASAS-5of6
Timepoint	3 months
N (countries)	12
N (patients)	4183

		ASAS-5of6	
		No	Yes
Smoking	No	2154	707
	Yes	978	344

OR	95%CI-Lo	95%CI-Hi
1.07	0.92	1.24

Model 2 ---------------------------- Model 1, adjusted for: age, gender, country, calendar year, disease duration and disease activity

N (countries)	10
N (patients)	3759

Reference country = Switzerland (CH)
Blank line = No data for this analysis

	OR	95%CI-Lo	95%CI-Hi
Smoking = Yes	0.78	0.66	0.92
Age	0.98	0.97	0.98
Male	1.59	1.35	1.87
BASDAI	1.01	1.00	1.01
DiseaseDuration_2	0.74	0.59	0.93
DiseaseDuration_3	1.03	0.84	1.27
DiseaseDuration_4	0.83	0.65	1.06
DiseaseDuration_5	0.75	0.47	1.17
CalendarYear	1.02	0.99	1.06
Czech Republic (CZ)	4.11	2.08	8.43
Denmark (DK)	0.78	0.54	1.14
Spain (ES)			
Iceland (IS)	0.71	0.18	2.55
Italy (IT)			
Netherlands (NL)			
Norway (NO)			
Portugal (PT)	0.08	0.04	0.17
Romania (RO)	0.79	0.23	2.39
Finland (SF)	0.42	0.19	0.85
Slovenia (SI)	0.38	0.24	0.61
Turkey (TR)	3.09	2.04	4.71
United Kingdom (UK)	0.57	0.34	0.94

Model 3a/3b ---------------------- Model 2 additionally adjusted, in turn, for Alcohol and BMI

Individual models. Result = OR (Smoking) from each model

		OR (smoking)	95%CI-Lo	95%CI-Hi	OR↑↓	N	N↓
(3a)	Alcohol	0.61	0.44	0.83	0.169	1234	2525 (67%)
(3b)	BMI	0.65	0.52	0.82	0.128	2080	1679 (45%)

Sensitivity analysis -------------- Model 2 additionally adjusted, in turn, for other variables shown

Individual models. Result = OR (Smoking) from each model

	OR (smoking)	95%CI-Lo	95%CI-Hi	OR↑↓	N	N↓
Yrs since symptom onset	0.79	0.65	0.96	0.015	2605	1154 (31%)
HLA-B27 status	0.72	0.55	0.92	0.062	2039	1720 (46%)
ESR	0.44	0.26	0.73	0.334	1240	2519 (67%)
CRP	0.69	0.57	0.84	0.088	3442	317 (8%)
mNY criteria	0.68	0.51	0.90	0.100	1476	2283 (61%)
ASAS criteria	0.77	0.61	0.98	0.009	1880	1879 (50%)
IBD	0.73	0.57	0.93	0.049	1946	1813 (48%)
Uveitis	0.75	0.58	0.95	0.033	1919	1840 (49%)
Psoriasis	0.71	0.56	0.90	0.067	1956	1803 (48%)
Comorbidity cardiovascular	0.54	0.35	0.82	0.237	1153	2606 (69%)
Comorbidity kidney	0.60	0.38	0.94	0.179	1075	2684 (71%)
Comorbidity diabetes	0.59	0.39	0.89	0.188	1169	2590 (69%)
BASFI	0.78	0.66	0.93	0.004	3351	408 (11%)
BASMI (3pt)	0.78	0.58	1.04	0.001	1119	2640 (70%)
Patient global	0.78	0.65	0.93	0.000	3560	199 (5%)
Physician global	0.77	0.64	0.92	0.008	3218	541 (14%)
Pain (VAS)	0.77	0.64	0.91	0.014	3490	269 (7%)
Fatigue	0.78	0.66	0.93	0.005	3684	75 (2%)
HAQ	0.77	0.62	0.95	0.012	2312	1447 (38%)
csDMARD	0.53	0.38	0.73	0.249	1276	2483 (66%)
NSAID	0.59	0.42	0.83	0.189	1024	2735 (73%)

(red) = Highest OR value
(blue) = Lowest OR value

Supplementary data: Association between SMOKING at baseline and BASDAI-50 at 12 months

Model 1 ---------------------------- Crude (unadjusted) association

Exposure	Smoking
Outcome	BASDAI-50
Timepoint	12 months
N (countries)	13
N (patients)	6899

		BASDAI-50	
		No	Yes
Smoking	No	2282	2649
	Yes	978	990

OR	95%CI-Lo	95%CI-Hi
0.87	0.79	0.97

Model 2 ---------------------------- Model 1, adjusted for: age, gender, country, calendar year, disease duration and disease activity

N (countries)	13
N (patients)	6886

Reference country = Switzerland (CH)
Blank line = No data for this analysis

	OR	95%CI-Lo	95%CI-Hi
Smoking = Yes	0.77	0.68	0.86
Age	0.97	0.97	0.98
Male	1.68	1.51	1.88
BASDAI	1.01	1.01	1.01
DiseaseDuration_2	0.81	0.70	0.95
DiseaseDuration_3	0.89	0.77	1.02
DiseaseDuration_4	0.91	0.78	1.06
DiseaseDuration_5	1.02	0.79	1.30
CalendarYear	1.00	0.98	1.02
Czech Republic (CZ)	2.67	2.08	3.43
Denmark (DK)	0.68	0.54	0.86
Spain (ES)	0.73	0.53	1.01
Iceland (IS)	1.06	0.45	2.54
Italy (IT)	1.09	0.81	1.47
Netherlands (NL)			
Norway (NO)	1.12	0.88	1.44
Portugal (PT)	1.29	0.98	1.69
Romania (RO)	9.79	5.57	18.30
Finland (SF)	1.04	0.70	1.54
Slovenia (SI)	1.41	1.05	1.89
Turkey (TR)	4.60	3.42	6.22
United Kingdom (UK)	0.80	0.54	1.17

Model 3a/3b ---------------------- Model 2 additionally adjusted, in turn, for Alcohol and BMI

Individual models. Result = OR (Smoking) from each model

		OR (smoking)	95%CI-Lo	95%CI-Hi	OR↑↓	N	N↓
(3a)	Alcohol	0.69	0.55	0.87	0.078	1610	5276 (77%)
(3b)	BMI	0.66	0.57	0.77	0.102	4409	2477 (36%)

Sensitivity analysis -------------- Model 2 additionally adjusted, in turn, for other variables shown

Individual models. Result = OR (Smoking) from each model

	OR (smoking)	95%CI-Lo	95%CI-Hi	OR↑↓	N	N↓
Yrs since symptom onset	0.78	0.68	0.89	0.012	4657	2229 (32%)
HLA-B27 status	0.76	0.67	0.88	0.002	4641	2245 (33%)
ESR	0.72	0.61	0.85	0.044	3780	3106 (45%)
CRP	0.71	0.63	0.81	0.053	5781	1105 (16%)
mNY criteria	0.82	0.67	1.00	0.050	2259	4627 (67%)
ASAS criteria	0.80	0.68	0.94	0.032	3583	3303 (48%)
IBD	0.75	0.64	0.87	0.020	3878	3008 (44%)
Uveitis	0.75	0.65	0.87	0.014	4149	2737 (40%)
Psoriasis	0.75	0.64	0.87	0.021	3848	3038 (44%)
Comorbidity cardiovascular	0.72	0.61	0.86	0.043	3401	3485 (51%)
Comorbidity kidney	0.71	0.59	0.84	0.058	3326	3560 (52%)
Comorbidity diabetes	0.70	0.59	0.84	0.062	3414	3472 (50%)
BASFI	0.81	0.71	0.92	0.041	5507	1379 (20%)
BASMI (3pt)	0.77	0.60	0.98	0.004	1340	5546 (81%)
Patient global	0.77	0.68	0.87	0.001	5973	913 (13%)
Physician global	0.77	0.68	0.88	0.008	5333	1553 (23%)
Pain (VAS)	0.75	0.66	0.85	0.018	5689	1197 (17%)
Fatigue	0.76	0.67	0.85	0.009	6234	652 (9%)
HAQ	0.77	0.66	0.89	0.000	4100	2786 (40%)
csDMARD	0.67	0.55	0.81	0.099	2976	3910 (57%)
NSAID	0.79	0.66	0.95	0.026	2678	4208 (61%)

[red] = Highest OR value
[blue] = Lowest OR value

Supplementary data: Association between SMOKING at baseline and ASDAS-Inactive at 12 months

Model 1 ---------------------------- Crude (unadjusted) association

Exposure	Smoking
Outcome	ASDAS-Inactive
Timepoint	12 months
N (countries)	12
N (patients)	7126

		ASDAS-Inactive No	ASDAS-Inactive Yes
Smoking	No	3426	1557
	Yes	1591	552

OR	95%CI-Lo	95%CI-Hi
0.76	0.68	0.86

Model 2 ---------------------------- Model 1, adjusted for: age, gender, country, calendar year, disease duration and disease activity

N (countries)	12
N (patients)	5292

Reference country = Switzerland (CH)
Blank line = No data for this analysis

	OR	95%CI-Lo	95%CI-Hi
Smoking = Yes	0.68	0.59	0.79
Age	0.96	0.96	0.97
Male	1.76	1.53	2.02
BASDAI	0.98	0.97	0.98
DiseaseDuration_2	0.85	0.70	1.04
DiseaseDuration_3	1.07	0.90	1.27
DiseaseDuration_4	1.00	0.83	1.21
DiseaseDuration_5	0.78	0.57	1.06
CalendarYear	1.03	1.00	1.05
Czech Republic (CZ)	1.68	1.23	2.29
Denmark (DK)	0.91	0.68	1.23
Spain (ES)			
Iceland (IS)	0.58	0.13	1.88
Italy (IT)	1.80	1.04	3.07
Netherlands (NL)			
Norway (NO)	1.46	1.08	1.99
Portugal (PT)	1.15	0.82	1.63
Romania (RO)	2.58	1.62	4.11
Finland (SF)	1.32	0.73	2.33
Slovenia (SI)	0.97	0.51	1.73
Turkey (TR)	1.67	1.20	2.35
United Kingdom (UK)	1.08	0.66	1.77

Model 3a/3b ---------------------- Model 2 additionally adjusted, in turn, for Alcohol and BMI

Individual models. Result = OR (Smoking) from each model

		OR (smoking)	95%CI-Lo	95%CI-Hi	OR↑↓	N	N↓
(3a)	Alcohol	0.65	0.48	0.88	0.031	1406	3886 (73%)
(3b)	BMI	0.58	0.48	0.70	0.106	3149	2143 (40%)

Sensitivity analysis -------------- Model 2 additionally adjusted, in turn, for other variables shown

Individual models. Result = OR (Smoking) from each model

	OR (smoking)	95%CI-Lo	95%CI-Hi	OR↑↓	N	N↓
Yrs since symptom onset	0.73	0.62	0.85	0.044	4117	1175 (22%)
HLA-B27 status	0.65	0.54	0.77	0.035	3539	1753 (33%)
ESR	0.61	0.50	0.75	0.069	2698	2594 (49%)
CRP	0.66	0.57	0.77	0.022	4864	428 (8%)
mNY criteria	0.75	0.58	0.98	0.071	1760	3532 (67%)
ASAS criteria	0.62	0.51	0.76	0.061	2910	2382 (45%)
IBD	0.59	0.48	0.71	0.097	3064	2228 (42%)
Uveitis	0.62	0.51	0.76	0.058	3063	2229 (42%)
Psoriasis	0.61	0.50	0.74	0.070	3056	2236 (42%)
Comorbidity cardiovascular	0.58	0.46	0.71	0.108	2458	2834 (54%)
Comorbidity kidney	0.57	0.45	0.71	0.114	2338	2954 (56%)
Comorbidity diabetes	0.56	0.45	0.70	0.119	2466	2826 (53%)
BASFI	0.69	0.59	0.81	0.011	4342	950 (18%)
BASMI (3pt)	0.79	0.58	1.08	0.111	1217	4075 (77%)
Patient global	0.71	0.61	0.81	0.023	5147	145 (3%)
Physician global	0.70	0.60	0.81	0.013	4588	704 (13%)
Pain (VAS)	0.69	0.59	0.80	0.007	4720	572 (11%)
Fatigue	0.67	0.58	0.77	0.012	5206	86 (2%)
HAQ	0.69	0.58	0.82	0.010	3256	2036 (38%)
csDMARD	0.58	0.45	0.73	0.105	2115	3177 (60%)
NSAID	0.58	0.46	0.72	0.105	2236	3056 (58%)

(red) = Highest OR value
(blue) = Lowest OR value

Supplementary data: Association between SMOKING at baseline and ASDAS-LDA at 12 months

Model 1 ---------------------------- Crude (unadjusted) association

Exposure	Smoking
Outcome	ASDAS-LDA
Timepoint	12 months
N (countries)	12
N (patients)	7126

		ASDAS-LDA	
		No	Yes
Smoking	No	1862	3121
	Yes	900	1243

OR	95%CI-Lo	95%CI-Hi
0.82	0.74	0.91

Model 2 ---------------------------- Model 1, adjusted for: age, gender, country, calendar year, disease duration and disease activity

N (countries)	12
N (patients)	5292

Reference country = Switzerland (CH)
Blank line = No data for this analysis

	OR	95%CI-Lo	95%CI-Hi
Smoking = Yes	0.81	0.71	0.93
Age	0.97	0.97	0.98
Male	1.58	1.39	1.79
BASDAI	0.97	0.97	0.97
DiseaseDuration_2	0.81	0.67	0.98
DiseaseDuration_3	0.93	0.79	1.10
DiseaseDuration_4	0.91	0.76	1.09
DiseaseDuration_5	1.01	0.76	1.36
CalendarYear	1.00	0.98	1.03
Czech Republic (CZ)	2.33	1.74	3.12
Denmark (DK)	0.74	0.57	0.97
Spain (ES)			
Iceland (IS)	0.76	0.27	2.14
Italy (IT)	0.95	0.56	1.61
Netherlands (NL)			
Norway (NO)	1.26	0.95	1.68
Portugal (PT)	1.17	0.86	1.60
Romania (RO)	7.83	4.54	14.03
Finland (SF)	1.22	0.68	2.21
Slovenia (SI)	1.03	0.64	1.66
Turkey (TR)	1.53	1.10	2.13
United Kingdom (UK)	0.95	0.62	1.48

Model 3a/3b ---------------------- Model 2 additionally adjusted, in turn, for Alcohol and BMI

Individual models. Result = OR (Smoking) from each model

		OR (smoking)	95%CI-Lo	95%CI-Hi	OR↑↓	N	N↓
(3a)	Alcohol	0.73	0.56	0.94	0.080	1406	3886 (73%)
(3b)	BMI	0.73	0.61	0.87	0.079	3149	2143 (40%)

Sensitivity analysis -------------- Model 2 additionally adjusted, in turn, for other variables shown

Individual models. Result = OR (Smoking) from each model

	OR (smoking)	95%CI-Lo	95%CI-Hi	OR↑↓	N	N↓
Yrs since symptom onset	0.85	0.73	0.99	0.039	4117	1175 (22%)
HLA-B27 status	0.81	0.69	0.95	0.001	3539	1753 (33%)
ESR	0.78	0.64	0.95	0.033	2698	2594 (49%)
CRP	0.79	0.68	0.90	0.025	4864	428 (8%)
mNY criteria	0.80	0.64	1.01	0.011	1760	3532 (67%)
ASAS criteria	0.80	0.67	0.96	0.009	2910	2382 (45%)
IBD	0.81	0.68	0.97	0.000	3064	2228 (42%)
Uveitis	0.81	0.68	0.97	0.001	3063	2229 (42%)
Psoriasis	0.82	0.69	0.97	0.005	3056	2236 (42%)
Comorbidity cardiovascular	0.74	0.60	0.92	0.067	2458	2834 (54%)
Comorbidity kidney	0.74	0.60	0.92	0.072	2338	2954 (56%)
Comorbidity diabetes	0.73	0.59	0.89	0.084	2466	2826 (53%)
BASFI	0.86	0.74	1.00	0.051	4342	950 (18%)
BASMI (3pt)	0.84	0.64	1.10	0.031	1217	4075 (77%)
Patient global	0.84	0.73	0.96	0.029	5147	145 (3%)
Physician global	0.82	0.72	0.95	0.013	4588	704 (13%)
Pain (VAS)	0.82	0.72	0.95	0.012	4720	572 (11%)
Fatigue	0.81	0.71	0.93	0.002	5206	86 (2%)
HAQ	0.80	0.67	0.95	0.011	3256	2036 (38%)
csDMARD	0.82	0.65	1.02	0.006	2115	3177 (60%)
NSAID	0.77	0.62	0.94	0.045	2236	3056 (58%)

= Highest OR value (red)
= Lowest OR value (blue)

Supplementary data: Association between SMOKING at baseline and ASDAS-CII at 12 months

Model 1 ---------------------------- Crude (unadjusted) association

Exposure	Smoking
Outcome	ASDAS-CII
Timepoint	12 months
N (countries)	12
N (patients)	4838

		ASDAS-CII	
		No	Yes
Smoking	No	1344	1995
	Yes	569	930

OR	95%CI-Lo	95%CI-Hi
1.10	0.97	1.25

Model 2 ---------------------------- Model 1, adjusted for: age, gender, country, calendar year, disease duration and disease activity

N (countries)	12
N (patients)	4819

Reference country = Switzerland (CH)
Blank line = No data for this analysis

	OR	95%CI-Lo	95%CI-Hi
Smoking = Yes	0.95	0.82	1.09
Age	0.98	0.97	0.98
Male	1.93	1.68	2.21
BASDAI	1.03	1.02	1.03
DiseaseDuration_2	0.81	0.67	0.99
DiseaseDuration_3	0.88	0.74	1.05
DiseaseDuration_4	0.97	0.80	1.17
DiseaseDuration_5	0.96	0.71	1.29
CalendarYear	0.97	0.94	0.99
Czech Republic (CZ)	4.85	3.51	6.72
Denmark (DK)	0.74	0.57	0.97
Spain (ES)			
Iceland (IS)	1.04	0.36	3.25
Italy (IT)	1.49	0.81	2.79
Netherlands (NL)			
Norway (NO)	0.96	0.72	1.28
Portugal (PT)	1.55	1.13	2.13
Romania (RO)	9.59	5.05	19.66
Finland (SF)	0.74	0.36	1.48
Slovenia (SI)	0.87	0.52	1.48
Turkey (TR)	3.36	2.41	4.68
United Kingdom (UK)	1.13	0.71	1.80

Model 3a/3b ---------------------- Model 2 additionally adjusted, in turn, for Alcohol and BMI

Individual models. Result = OR (Smoking) from each model

		OR (smoking)	95%CI-Lo	95%CI-Hi	OR↑↓	N	N↓
(3a)	Alcohol	0.83	0.64	1.08	0.115	1265	3554 (74%)
(3b)	BMI	0.83	0.68	1.01	0.119	2864	1955 (41%)

Sensitivity analysis -------------- Model 2 additionally adjusted, in turn, for other variables shown

Individual models. Result = OR (Smoking) from each model

	OR (smoking)	95%CI-Lo	95%CI-Hi	OR↑↓	N	N↓
Yrs since symptom onset	0.99	0.84	1.16	0.041	3827	992 (21%)
HLA-B27 status	1.02	0.85	1.22	0.067	3239	1580 (33%)
ESR	0.92	0.74	1.16	0.024	2575	2244 (47%)
CRP	0.83	0.71	0.97	0.117	4732	87 (2%)
mNY criteria	0.94	0.73	1.20	0.011	1607	3212 (67%)
ASAS criteria	0.97	0.79	1.18	0.016	2684	2135 (44%)
IBD	1.00	0.82	1.22	0.054	2807	2012 (42%)
Uveitis	1.00	0.82	1.22	0.051	2814	2005 (42%)
Psoriasis	1.03	0.85	1.26	0.083	2798	2021 (42%)
Comorbidity cardiovascular	1.05	0.82	1.33	0.096	2267	2552 (53%)
Comorbidity kidney	1.06	0.82	1.36	0.108	2152	2667 (55%)
Comorbidity diabetes	1.05	0.83	1.33	0.099	2269	2550 (53%)
BASFI	0.97	0.83	1.14	0.025	3951	868 (18%)
BASMI (3pt)	0.91	0.70	1.19	0.039	1149	3670 (76%)
Patient global	0.92	0.80	1.06	0.028	4805	14 (0%)
Physician global	0.93	0.80	1.08	0.019	4282	537 (11%)
Pain (VAS)	0.92	0.79	1.06	0.033	4344	475 (10%)
Fatigue	0.95	0.82	1.09	0.000	4763	56 (1%)
HAQ	0.92	0.76	1.10	0.033	2990	1829 (38%)
csDMARD	0.87	0.68	1.10	0.081	1922	2897 (60%)
NSAID	0.97	0.77	1.23	0.023	2104	2715 (56%)

= Highest OR value
= Lowest OR value

Supplementary data: Association between SMOKING at baseline and ASDAS-MI at 12 months

Model 1 ---------------------------- Crude (unadjusted) association

Exposure	Smoking
Outcome	ASDAS-MI
Timepoint	12 months
N (countries)	12
N (patients)	4838

		ASDAS-MI No	ASDAS-MI Yes
Smoking	No	2178	1161
	Yes	952	547

OR	95%CI-Lo	95%CI-Hi
1.08	0.95	1.22

Model 2 ---------------------------- Model 1, adjusted for: age, gender, country, calendar year, disease duration and disease activity

N (countries)	12
N (patients)	4819

Reference country = Switzerland (CH)
Blank line = No data for this analysis

	OR	95%CI-Lo	95%CI-Hi
Smoking = Yes	0.95	0.82	1.10
Age	0.97	0.97	0.98
Male	1.75	1.52	2.03
BASDAI	1.04	1.04	1.05
DiseaseDuration_2	0.69	0.56	0.85
DiseaseDuration_3	0.89	0.74	1.07
DiseaseDuration_4	0.98	0.80	1.19
DiseaseDuration_5	0.85	0.59	1.20
CalendarYear	0.97	0.95	1.00
Czech Republic (CZ)	3.99	2.88	5.57
Denmark (DK)	0.88	0.65	1.22
Spain (ES)			
Iceland (IS)	0.82	0.23	2.56
Italy (IT)	0.54	0.20	1.24
Netherlands (NL)			
Norway (NO)	0.98	0.70	1.37
Portugal (PT)	1.51	1.07	2.15
Romania (RO)	11.60	6.72	20.49
Finland (SF)	1.64	0.73	3.50
Slovenia (SI)	0.95	0.52	1.70
Turkey (TR)	4.38	3.04	6.34
United Kingdom (UK)	0.96	0.56	1.63

Model 3a/3b ---------------------- Model 2 additionally adjusted, in turn, for Alcohol and BMI

Individual models. Result = OR (Smoking) from each model

		OR (smoking)	95%CI-Lo	95%CI-Hi	OR↑↓	N	N↓
(3a)	Alcohol	1.02	0.76	1.36	0.073	1265	3554 (74%)
(3b)	BMI	0.90	0.75	1.09	0.044	2864	1955 (41%)

Sensitivity analysis -------------- Model 2 additionally adjusted, in turn, for other variables shown

Individual models. Result = OR (Smoking) from each model

	OR (smoking)	95%CI-Lo	95%CI-Hi	OR↑↓	N	N↓
Yrs since symptom onset	1.02	0.87	1.20	0.074	3827	992 (21%)
HLA-B27 status	1.01	0.84	1.21	0.060	3239	1580 (33%)
ESR	0.93	0.74	1.16	0.018	2575	2244 (47%)
CRP	0.82	0.70	0.97	0.127	4732	87 (2%)
mNY criteria	1.05	0.81	1.35	0.098	1607	3212 (67%)
ASAS criteria	1.06	0.87	1.29	0.115	2684	2135 (44%)
IBD	1.00	0.82	1.21	0.051	2807	2012 (42%)
Uveitis	1.04	0.86	1.26	0.093	2814	2005 (42%)
Psoriasis	1.04	0.86	1.26	0.096	2798	2021 (42%)
Comorbidity cardiovascular	1.00	0.80	1.25	0.052	2267	2552 (53%)
Comorbidity kidney	0.96	0.77	1.22	0.017	2152	2667 (55%)
Comorbidity diabetes	0.96	0.77	1.20	0.014	2269	2550 (53%)
BASFI	0.98	0.84	1.15	0.035	3951	868 (18%)
BASMI (3pt)	0.94	0.69	1.26	0.012	1149	3670 (76%)
Patient global	0.91	0.79	1.06	0.035	4805	14 (0%)
Physician global	0.90	0.78	1.05	0.043	4282	537 (11%)
Pain (VAS)	0.90	0.77	1.05	0.051	4344	475 (10%)
Fatigue	0.95	0.82	1.10	0.003	4763	56 (1%)
HAQ	0.92	0.77	1.11	0.023	2990	1829 (38%)
csDMARD	0.98	0.76	1.25	0.031	1922	2897 (60%)
NSAID	0.99	0.80	1.23	0.044	2104	2715 (56%)

= Highest OR value (red)
= Lowest OR value (blue)

Supplementary data: Association between SMOKING at baseline and ASAS-20 at 12 months

Model 1 ---------------------------- Crude (unadjusted) association

Exposure	Smoking
Outcome	ASAS-20
Timepoint	12 months
N (countries)	12
N (patients)	5089

		ASAS-20 No	ASAS-20 Yes
Smoking	No	1750	1708
	Yes	780	851

OR	95%CI-Lo	95%CI-Hi
1.12	0.99	1.26

Model 2 ---------------------------- Model 1, adjusted for: age, gender, country, calendar year, disease duration and disease activity

N (countries)	8
N (patients)	4716

Reference country = Switzerland (CH)
Blank line = No data for this analysis

	OR	95%CI-Lo	95%CI-Hi
Smoking = Yes	0.87	0.75	1.00
Age	0.98	0.97	0.98
Male	1.49	1.29	1.71
BASDAI	1.02	1.02	1.03
DiseaseDuration_2	0.72	0.59	0.88
DiseaseDuration_3	0.88	0.74	1.06
DiseaseDuration_4	0.92	0.76	1.13
DiseaseDuration_5	0.68	0.46	1.01
CalendarYear	1.02	0.99	1.05
Czech Republic (CZ)	1.95	1.46	2.61
Denmark (DK)	0.57	0.44	0.73
Spain (ES)			
Iceland (IS)	0.63	0.25	1.63
Italy (IT)			
Netherlands (NL)			
Norway (NO)			
Portugal (PT)			
Romania (RO)			
Finland (SF)	0.45	0.28	0.73
Slovenia (SI)	0.29	0.19	0.44
Turkey (TR)	2.31	1.68	3.19
United Kingdom (UK)	0.94	0.62	1.42

Model 3a/3b ---------------------- Model 2 additionally adjusted, in turn, for Alcohol and BMI

Individual models. Result = OR (Smoking) from each model

		OR (smoking)	95%CI-Lo	95%CI-Hi	OR↑↓	N	N↓
(3a)	Alcohol	0.82	0.62	1.08	0.048	1219	3497 (74%)
(3b)	BMI	0.81	0.68	0.96	0.062	3134	1582 (34%)

Sensitivity analysis --------------- Model 2 additionally adjusted, in turn, for other variables shown

Individual models. Result = OR (Smoking) from each model

	OR (smoking)	95%CI-Lo	95%CI-Hi	OR↑↓	N	N↓
Yrs since symptom onset	0.87	0.74	1.03	0.003	3669	1047 (22%)
HLA-B27 status	0.86	0.71	1.04	0.008	2881	1835 (39%)
ESR	0.86	0.65	1.14	0.012	1952	2764 (59%)
CRP	0.80 (lowest, blue)	0.69	0.94	0.067	4249	467 (10%)
mNY criteria	0.86	0.67	1.10	0.008	1491	3225 (68%)
ASAS criteria	0.85	0.70	1.02	0.024	2642	2074 (44%)
IBD	0.83	0.69	1.00	0.043	2819	1897 (40%)
Uveitis	0.82	0.69	0.99	0.044	2821	1895 (40%)
Psoriasis	0.85	0.71	1.02	0.020	2812	1904 (40%)
Comorbidity cardiovascular	0.88	0.68	1.15	0.012	1880	2836 (60%)
Comorbidity kidney	0.88	0.66	1.17	0.008	1751	2965 (63%)
Comorbidity diabetes	0.86	0.66	1.12	0.012	1901	2815 (60%)
BASFI	0.87	0.75	1.01	0.005	4404	312 (7%)
BASMI (3pt)	0.81	0.62	1.06	0.058	1149	3567 (76%)
Patient global	0.84	0.73	0.98	0.025	4588	128 (3%)
Physician global	0.82	0.70	0.95	0.050	4279	437 (9%)
Pain (VAS)	0.81	0.70	0.94	0.055	4508	208 (4%)
Fatigue	0.87	0.75	1.00	0.000	4609	107 (2%)
HAQ	0.81	0.67	0.98	0.060	2924	1792 (38%)
csDMARD	0.94 (highest, red)	0.73	1.21	0.073	1788	2928 (62%)
NSAID	0.85	0.67	1.08	0.022	1927	2789 (59%)

(red) = Highest OR value
(blue) = Lowest OR value

Supplementary data: Association between SMOKING at baseline and ASAS-40 at 12 months

Model 1 ---------------------------- Crude (unadjusted) association

Exposure	Smoking
Outcome	ASAS-40
Timepoint	12 months
N (countries)	12
N (patients)	5706

		ASAS-40 No	ASAS-40 Yes
Smoking	No	2655	1255
	Yes	1181	615

OR	95%CI-Lo	95%CI-Hi
1.10	0.98	1.24

Model 2 ---------------------------- Model 1, adjusted for: age, gender, country, calendar year, disease duration and disease activity

N (countries)	8
N (patients)	5146

Reference country = Switzerland (CH)
Blank line = No data for this analysis

	OR	95%CI-Lo	95%CI-Hi
Smoking = Yes	0.83	0.72	0.95
Age	0.97	0.97	0.98
Male	1.48	1.28	1.70
BASDAI	1.03	1.02	1.03
DiseaseDuration_2	0.81	0.67	0.99
DiseaseDuration_3	0.81	0.68	0.97
DiseaseDuration_4	0.93	0.76	1.13
DiseaseDuration_5	0.68	0.44	1.02
CalendarYear	1.02	0.99	1.05
Czech Republic (CZ)	2.10	1.60	2.77
Denmark (DK)	0.62	0.48	0.79
Spain (ES)			
Iceland (IS)	0.89	0.35	2.20
Italy (IT)			
Netherlands (NL)			
Norway (NO)			
Portugal (PT)			
Romania (RO)			
Finland (SF)	0.42	0.24	0.69
Slovenia (SI)	0.16	0.09	0.25
Turkey (TR)	2.40	1.76	3.27
United Kingdom (UK)	0.92	0.61	1.38

Model 3a/3b ---------------------- Model 2 additionally adjusted, in turn, for Alcohol and BMI

Individual models. Result = OR (Smoking) from each model

		OR (smoking)	95%CI-Lo	95%CI-Hi	OR↑↓	N	N↓
(3a)	Alcohol	0.72	0.54	0.96	0.104	1311	3835 (75%)
(3b)	BMI	0.75	0.63	0.89	0.077	3327	1819 (35%)

Sensitivity analysis -------------- Model 2 additionally adjusted, in turn, for other variables shown

Individual models. Result = OR (Smoking) from each model

	OR (smoking)	95%CI-Lo	95%CI-Hi	OR↑↓	N	N↓
Yrs since symptom onset	0.85	0.72	0.99	0.020	3885	1261 (25%)
HLA-B27 status	0.79	0.66	0.95	0.037	3214	1932 (38%)
ESR	0.76	0.59	0.98	0.063	2324	2822 (55%)
CRP	0.75	0.64	0.87	0.080	4608	538 (10%)
mNY criteria	0.74	0.57	0.95	0.090	1638	3508 (68%)
ASAS criteria	0.79	0.66	0.95	0.032	2825	2321 (45%)
IBD	0.79	0.66	0.94	0.038	3029	2117 (41%)
Uveitis	0.78	0.65	0.93	0.050	3025	2121 (41%)
Psoriasis	0.78	0.65	0.94	0.042	3016	2130 (41%)
Comorbidity cardiovascular	0.80	0.62	1.02	0.028	2165	2981 (58%)
Comorbidity kidney	0.78	0.60	1.01	0.044	2034	3112 (60%)
Comorbidity diabetes	0.81	0.63	1.03	0.019	2182	2964 (58%)
BASFI	0.83	0.72	0.96	0.003	4652	494 (10%)
BASMI (3pt)	0.65	0.49	0.86	0.174	1199	3947 (77%)
Patient global	0.79	0.68	0.92	0.036	4912	234 (5%)
Physician global	0.79	0.68	0.91	0.039	4561	585 (11%)
Pain (VAS)	0.77	0.66	0.89	0.058	4804	342 (7%)
Fatigue	0.83	0.72	0.95	0.000	4993	153 (3%)
HAQ	0.82	0.69	0.98	0.004	3208	1938 (38%)
csDMARD	0.80	0.63	1.03	0.021	2006	3140 (61%)
NSAID	0.75	0.60	0.94	0.072	2064	3082 (60%)

(red) = Highest OR value
(blue) = Lowest OR value

Supplementary data: Association between SMOKING at baseline and ASAS-5of6 at 12 months

Model 1 ---------------------------- Crude (unadjusted) association

Exposure	Smoking
Outcome	ASAS-5of6
Timepoint	12 months
N (countries)	12
N (patients)	4184

		ASAS-5of6	
		No	Yes
Smoking	No	1777	1078
	Yes	803	526

OR	95%CI-Lo	95%CI-Hi
1.08	0.94	1.23

Model 2 ---------------------------- Model 1, adjusted for: age, gender, country, calendar year, disease duration and disease activity

N (countries)	11
N (patients)	3834

Reference country = Switzerland (CH)
Blank line = No data for this analysis

Rounding to 2dp. Actual value = non-zero.

	OR	95%CI-Lo	95%CI-Hi
Smoking = Yes	0.82	0.69	0.96
Age	0.97	0.97	0.98
Male	1.65	1.41	1.94
BASDAI	1.02	1.01	1.02
DiseaseDuration_2	0.75	0.60	0.95
DiseaseDuration_3	0.92	0.75	1.12
DiseaseDuration_4	0.91	0.72	1.14
DiseaseDuration_5	0.69	0.43	1.10
CalendarYear	1.03	1.00	1.07
Czech Republic (CZ)	5.35	3.90	7.38
Denmark (DK)	0.86	0.65	1.15
Spain (ES)			
Iceland (IS)	1.18	0.36	3.72
Italy (IT)	0.00	0.00	0.41
Netherlands (NL)			
Norway (NO)			
Portugal (PT)	0.11	0.05	0.21
Romania (RO)	1.61	0.78	3.38
Finland (SF)	0.58	0.30	1.07
Slovenia (SI)	0.46	0.30	0.68
Turkey (TR)	4.25	2.96	6.12
United Kingdom (UK)	0.90	0.55	1.47

Model 3a/3b ---------------------- Model 2 additionally adjusted, in turn, for Alcohol and BMI

Individual models. Result = OR (Smoking) from each model

		OR (smoking)	95%CI-Lo	95%CI-Hi	OR↑↓	N	N↓
(3a)	Alcohol	0.75	0.54	1.04	0.067	932	2902 (76%)
(3b)	BMI	0.73	0.60	0.89	0.085	2575	1259 (33%)

Sensitivity analysis -------------- Model 2 additionally adjusted, in turn, for other variables shown

Individual models. Result = OR (Smoking) from each model

	OR (smoking)	95%CI-Lo	95%CI-Hi	OR↑↓	N	N↓
Yrs since symptom onset	0.84	0.70	1.00	0.018	2903	931 (24%)
HLA-B27 status	0.84	0.68	1.04	0.027	2465	1369 (36%)
ESR	0.83	0.62	1.10	0.008	1868	1966 (51%)
CRP	0.72	0.60	0.86	0.101	3611	223 (6%)
mNY criteria	0.85	0.65	1.11	0.031	1333	2501 (65%)
ASAS criteria	0.85	0.68	1.04	0.027	2270	1564 (41%)
IBD	0.82	0.66	1.01	0.001	2404	1430 (37%)
Uveitis	0.85	0.69	1.04	0.031	2408	1426 (37%)
Psoriasis	0.86	0.70	1.06	0.042	2403	1431 (37%)
Comorbidity cardiovascular	0.83	0.63	1.09	0.009	1764	2070 (54%)
Comorbidity kidney	0.83	0.62	1.11	0.012	1650	2184 (57%)
Comorbidity diabetes	0.84	0.64	1.10	0.020	1777	2057 (54%)
BASFI	0.82	0.70	0.97	0.004	3507	327 (9%)
BASMI (3pt)	0.79	0.58	1.07	0.029	912	2922 (76%)
Patient global	0.80	0.67	0.95	0.018	3648	186 (5%)
Physician global	0.77	0.65	0.92	0.044	3395	439 (11%)
Pain (VAS)	0.75	0.64	0.89	0.064	3608	226 (6%)
Fatigue	0.82	0.69	0.97	0.003	3758	76 (2%)
HAQ	0.76	0.61	0.94	0.058	2393	1441 (38%)
csDMARD	0.77	0.59	1.02	0.044	1514	2320 (61%)
NSAID	0.76	0.59	0.99	0.053	1628	2206 (58%)

[red] = Highest OR value
[blue] = Lowest OR value

Supplementary data: Association between BMI at baseline and BASDAI-50 at 3 months

Model 1 ---------------------------- Crude (unadjusted) association

Exposure	BMI
Outcome	BASDAI-50
Timepoint	3 months
N (countries)	13
N (patients)	3729

		BASDAI-50	
		No	Yes
BMI	Normal	870	734
	Overweight	834	517
	Obese	542	232

OR	95%CI-Lo	95%CI-Hi
0.73	0.63	0.85
0.51	0.42	0.61

Model 2 ---------------------------- Model 1, adjusted for: age, gender, country, calendar year, disease duration and disease activity

N (countries) 13
N (patients) 3727

Reference country = Switzerland (CH)
Blank line = No data for this analysis

	OR	95%CI-Lo	95%CI-Hi
BMI = Overweight	0.75	0.64	0.88
BMI = Obese	0.55	0.46	0.68
Age	0.98	0.97	0.98
Male	1.60	1.38	1.84
BASDAI	1.00	1.00	1.01
DiseaseDuration_2	0.83	0.68	1.03
DiseaseDuration_3	1.13	0.94	1.35
DiseaseDuration_4	0.97	0.79	1.19
DiseaseDuration_5	1.70	1.10	2.62
CalendarYear	1.02	1.00	1.05
Czech Republic (CZ)	1.71	0.97	3.04
Denmark (DK)	0.63	0.46	0.87
Spain (ES)	0.45	0.11	1.48
Iceland (IS)	1.39	0.32	6.08
Italy (IT)	0.66	0.46	0.95
Netherlands (NL)	0.66	0.34	1.27
Norway (NO)			
Portugal (PT)	0.97	0.66	1.42
Romania (RO)	5.86	3.21	11.10
Finland (SF)	1.06	0.74	1.54
Slovenia (SI)	1.35	0.95	1.92
Turkey (TR)	1.88	1.31	2.71
United Kingdom (UK)	0.54	0.35	0.82

Model 3a/3b ---------------------- Model 2 additionally adjusted, in turn, for Smoking and Alcohol

Individual models. Result = OR (BMI) from each model

		OR (over-wt)	95%CI-Lo	95%CI-Hi	OR (ov)↑↓	OR (obese)	95%CI-Lo	95%CI-Hi	OR (ob)↑↓	N	N↓
(3a)	Smoking	0.72	0.61	0.86	0.028	0.54	0.43	0.66	0.020	3263	464 (12%)
(3b)	Alcohol	0.66	0.52	0.85	0.090	0.49	0.35	0.67	0.067	1527	2200 (59%)

Sensitivity analysis -------------- Model 2 additionally adjusted, in turn, for other variables shown

Individual models. Result = OR (BMI) from each model

	OR (over-wt)	95%CI-Lo	95%CI-Hi	OR (ov)↑↓	OR (obese)	95%CI-Lo	95%CI-Hi	OR (ob)↑↓	N	N↓
Yrs since symptom onset	0.70	0.57	0.86	0.049	0.51	0.40	0.66	0.041	2282	1445 (39%)
HLA-B27 status	0.89	0.72	1.10	0.137	0.70	0.54	0.90	0.141	2110	1617 (43%)
ESR	0.80	0.64	1.02	0.052	0.64	0.48	0.86	0.089	1834	1893 (51%)
CRP	0.74	0.62	0.89	0.008	0.50	0.40	0.63	0.051	2961	766 (21%)
mNY criteria	0.84	0.67	1.06	0.089	0.65	0.49	0.86	0.096	1859	1868 (50%)
ASAS criteria	0.78	0.63	0.96	0.023	0.62	0.48	0.80	0.065	2090	1637 (44%)
IBD	0.79	0.65	0.96	0.040	0.57	0.45	0.73	0.018	2496	1231 (33%)
Uveitis	0.85	0.70	1.03	0.095	0.62	0.49	0.79	0.068	2436	1291 (35%)
Psoriasis	0.82	0.67	0.99	0.065	0.57	0.45	0.72	0.014	2456	1271 (34%)
Comorbidity cardiovascular	0.83	0.65	1.05	0.074	0.57	0.43	0.77	0.020	1669	2058 (55%)
Comorbidity kidney	0.84	0.66	1.08	0.090	0.58	0.42	0.78	0.023	1560	2167 (58%)
Comorbidity diabetes	0.86	0.67	1.09	0.107	0.66	0.49	0.88	0.100	1689	2038 (55%)
BASFI	0.77	0.66	0.91	0.020	0.60	0.49	0.74	0.050	3527	200 (5%)
BASMI (3pt)	0.64	0.45	0.90	0.115	0.30	0.19	0.48	0.253	805	2922 (78%)
Patient global	0.72	0.60	0.85	0.034	0.56	0.45	0.70	0.009	3173	554 (15%)
Physician global	0.71	0.59	0.86	0.044	0.58	0.45	0.73	0.021	2602	1125 (30%)
Pain (VAS)	0.73	0.62	0.87	0.020	0.58	0.47	0.71	0.025	3210	517 (14%)
Fatigue	0.75	0.63	0.89	0.004	0.56	0.45	0.69	0.003	3280	447 (12%)
HAQ	0.85	0.68	1.05	0.094	0.61	0.47	0.80	0.058	1913	1814 (49%)
csDMARD	0.83	0.66	1.04	0.078	0.54	0.41	0.72	0.013	1903	1824 (49%)
NSAID	0.62	0.46	0.84	0.132	0.58	0.40	0.85	0.026	1031	2696 (72%)

= Highest OR value
= Lowest OR value

Supplementary data: Association between BMI at baseline and ASDAS-Inactive at 3 months

Model 1 ---------------------------- Crude (unadjusted) association

Exposure	BMI
Outcome	ASDAS-Inactive
Timepoint	3 months
N (countries)	12
N (patients)	3717

		ASDAS-Inactive	
		No	Yes
BMI	Normal	1114	471
	Overweight	1053	292
	Obese	702	85

OR	95%CI-Lo	95%CI-Hi
0.66	0.55	0.78
0.29	0.22	0.37

Model 2 ---------------------------- Model 1, adjusted for: age, gender, country, calendar year, disease duration and disease activity

N (countries)	12
N (patients)	2675

Reference country = Switzerland (CH)
Blank line = No data for this analysis

	OR	95%CI-Lo	95%CI-Hi
BMI = Overweight	0.66	0.53	0.82
BMI = Obese	0.38	0.28	0.52
Age	0.97	0.96	0.98
Male	1.70	1.38	2.10
BASDAI	0.97	0.97	0.98
DiseaseDuration_2	0.92	0.68	1.24
DiseaseDuration_3	1.24	0.96	1.60
DiseaseDuration_4	0.90	0.66	1.23
DiseaseDuration_5	1.74	0.96	3.08
CalendarYear	1.07	1.03	1.11
Czech Republic (CZ)	2.52	1.29	4.90
Denmark (DK)	0.75	0.49	1.15
Spain (ES)			
Iceland (IS)	2.86	0.36	18.38
Italy (IT)	0.69	0.35	1.37
Netherlands (NL)	0.39	0.10	1.15
Norway (NO)			
Portugal (PT)	1.42	0.87	2.33
Romania (RO)	1.86	0.98	3.53
Finland (SF)	1.67	1.00	2.81
Slovenia (SI)	0.78	0.38	1.52
Turkey (TR)	0.82	0.51	1.32
United Kingdom (UK)	0.59	0.32	1.08

Model 3a/3b ---------------------- Model 2 additionally adjusted, in turn, for Smoking and Alcohol

Individual models. Result = OR (BMI) from each model

		OR (over-wt)	95%CI-Lo	95%CI-Hi	OR (ov)↑↓	OR (obese)	95%CI-Lo	95%CI-Hi	OR (ob)↑↓	N	N↓
(3a)	Smoking	0.64	0.50	0.80	0.027	0.37	0.26	0.51	0.011	2469	206 (8%)
(3b)	Alcohol	0.59	0.43	0.82	0.070	0.30	0.17	0.49	0.081	1348	1327 (50%)

Sensitivity analysis -------------- Model 2 additionally adjusted, in turn, for other variables shown

Individual models. Result = OR (BMI) from each model

	OR (over-wt)	95%CI-Lo	95%CI-Hi	OR (ov)↑↓	OR (obese)	95%CI-Lo	95%CI-Hi	OR (ob)↑↓	N	N↓
Yrs since symptom onset	0.64	0.50	0.81	0.025	0.35	0.24	0.50	0.028	2068	607 (23%)
HLA-B27 status	0.74	0.55	0.98	0.074	0.38	0.25	0.58	0.002	1564	1111 (42%)
ESR	0.72	0.50	1.02	0.053	0.40	0.24	0.65	0.017	1047	1628 (61%)
CRP	0.64	0.51	0.81	0.024	0.36	0.26	0.50	0.018	2388	287 (11%)
mNY criteria	0.86	0.62	1.19	0.193	0.44	0.26	0.71	0.055	1345	1330 (50%)
ASAS criteria	0.70	0.53	0.94	0.042	0.42	0.27	0.64	0.044	1615	1060 (40%)
IBD	0.69	0.53	0.91	0.031	0.37	0.24	0.54	0.016	1831	844 (32%)
Uveitis	0.74	0.56	0.97	0.073	0.42	0.28	0.62	0.042	1801	874 (33%)
Psoriasis	0.73	0.56	0.96	0.070	0.42	0.28	0.62	0.041	1823	852 (32%)
Comorbidity cardiovascular	0.74	0.52	1.05	0.073	0.42	0.26	0.67	0.043	1098	1577 (59%)
Comorbidity kidney	0.74	0.51	1.06	0.076	0.42	0.25	0.68	0.034	1007	1668 (62%)
Comorbidity diabetes	0.76	0.53	1.08	0.097	0.42	0.26	0.67	0.040	1117	1558 (58%)
BASFI	0.67	0.53	0.84	0.004	0.43	0.31	0.59	0.048	2521	154 (6%)
BASMI (3pt)	0.67	0.43	1.01	0.002	0.28	0.14	0.54	0.097	733	1942 (73%)
Patient global	0.66	0.53	0.83	0.000	0.37	0.27	0.50	0.013	2604	71 (3%)
Physician global	0.65	0.51	0.84	0.008	0.40	0.28	0.57	0.022	2090	585 (22%)
Pain (VAS)	0.61	0.48	0.78	0.049	0.38	0.27	0.52	0.002	2364	311 (12%)
Fatigue	0.65	0.52	0.81	0.012	0.40	0.29	0.54	0.016	2578	97 (4%)
HAQ	0.60	0.44	0.81	0.066	0.42	0.27	0.63	0.034	1278	1397 (52%)
csDMARD	0.73	0.53	1.01	0.067	0.43	0.28	0.67	0.053	1280	1395 (52%)
NSAID	0.68	0.46	1.00	0.020	0.43	0.24	0.74	0.048	900	1775 (66%)

[red] = Highest OR value
[blue] = Lowest OR value

Supplementary data: Association between BMI at baseline and ASDAS-LDA at 3 months

Model 1 ---------------------------- Crude (unadjusted) association

Exposure	BMI
Outcome	ASDAS-LDA
Timepoint	3 months
N (countries)	12
N (patients)	3717

		ASDAS-LDA	
		No	Yes
BMI	Normal	622	963
	Overweight	684	661
	Obese	511	276

OR	95%CI-Lo	95%CI-Hi
0.62	0.54	0.72
0.35	0.29	0.42

Model 2 ---------------------------- Model 1, adjusted for: age, gender, country, calendar year, disease duration and disease activity

N (countries)	12
N (patients)	2675

Reference country = Switzerland (CH)
Blank line = No data for this analysis

	OR	95%CI-Lo	95%CI-Hi
BMI = Overweight	0.61	0.50	0.74
BMI = Obese	0.42	0.33	0.53
Age	0.98	0.97	0.98
Male	1.40	1.18	1.67
BASDAI	0.97	0.96	0.97
DiseaseDuration_2	1.07	0.83	1.37
DiseaseDuration_3	1.07	0.86	1.34
DiseaseDuration_4	0.97	0.75	1.25
DiseaseDuration_5	1.26	0.73	2.18
CalendarYear	1.03	1.00	1.07
Czech Republic (CZ)	1.17	0.63	2.21
Denmark (DK)	0.55	0.38	0.80
Spain (ES)			
Iceland (IS)	1.30	0.20	10.34
Italy (IT)	0.85	0.47	1.53
Netherlands (NL)	0.70	0.32	1.54
Norway (NO)			
Portugal (PT)	0.89	0.57	1.37
Romania (RO)	2.97	1.62	5.52
Finland (SF)	1.19	0.73	1.96
Slovenia (SI)	0.66	0.39	1.10
Turkey (TR)	0.82	0.53	1.26
United Kingdom (UK)	0.61	0.38	0.97

Model 3a/3b ---------------------- Model 2 additionally adjusted, in turn, for Smoking and Alcohol

Individual models. Result = OR (BMI) from each model

		OR (over-wt)	95%CI-Lo	95%CI-Hi	OR (ov)↑↓	OR (obese)	95%CI-Lo	95%CI-Hi	OR (ob)↑↓	N	N↓
(3a)	Smoking	0.59	0.48	0.72	0.019	0.40	0.31	0.51	0.025	2469	206 (8%)
(3b)	Alcohol	0.62	0.47	0.81	0.010	0.43	0.31	0.61	0.010	1348	1327 (50%)

Sensitivity analysis -------------- Model 2 additionally adjusted, in turn, for other variables shown

Individual models. Result = OR (BMI) from each model

	OR (over-wt)	95%CI-Lo	95%CI-Hi	OR (ov)↑↓	OR (obese)	95%CI-Lo	95%CI-Hi	OR (ob)↑↓	N	N↓
Yrs since symptom onset	0.59	0.48	0.74	0.015	0.38	0.29	0.50	0.037	2068	607 (23%)
HLA-B27 status	0.68	0.53	0.88	0.073	0.53	0.39	0.72	0.109	1564	1111 (42%)
ESR	0.58	0.42	0.80	0.026	0.50	0.34	0.74	0.081	1047	1628 (61%)
CRP	0.60	0.49	0.73	0.014	0.40	0.31	0.52	0.018	2388	287 (11%)
mNY criteria	0.76	0.58	1.00	0.153	0.56	0.40	0.79	0.143	1345	1330 (50%)
ASAS criteria	0.69	0.54	0.88	0.081	0.51	0.38	0.69	0.092	1615	1060 (40%)
IBD	0.65	0.51	0.82	0.039	0.48	0.36	0.64	0.055	1831	844 (32%)
Uveitis	0.72	0.57	0.91	0.110	0.53	0.40	0.71	0.109	1801	874 (33%)
Psoriasis	0.70	0.55	0.88	0.089	0.50	0.37	0.66	0.077	1823	852 (32%)
Comorbidity cardiovascular	0.65	0.48	0.89	0.040	0.51	0.35	0.74	0.089	1098	1577 (59%)
Comorbidity kidney	0.66	0.48	0.91	0.052	0.52	0.35	0.77	0.102	1007	1668 (62%)
Comorbidity diabetes	0.69	0.50	0.94	0.080	0.57	0.40	0.83	0.152	1117	1558 (58%)
BASFI	0.63	0.52	0.77	0.022	0.47	0.36	0.60	0.044	2521	154 (6%)
BASMI (3pt)	0.63	0.44	0.91	0.020	0.35	0.21	0.56	0.076	733	1942 (73%)
Patient global	0.60	0.49	0.73	0.007	0.41	0.34	0.52	0.009	2604	71 (3%)
Physician global	0.60	0.48	0.74	0.009	0.42	0.32	0.54	0.005	2090	585 (22%)
Pain (VAS)	0.60	0.49	0.74	0.008	0.41	0.32	0.53	0.010	2364	311 (12%)
Fatigue	0.61	0.50	0.74	0.003	0.43	0.34	0.55	0.013	2578	97 (4%)
HAQ	0.55	0.42	0.74	0.055	0.39	0.27	0.54	0.034	1278	1397 (52%)
csDMARD	0.61	0.45	0.81	0.001	0.42	0.30	0.58	0.005	1280	1395 (52%)
NSAID	0.58	0.42	0.81	0.027	0.43	0.28	0.65	0.006	900	1775 (66%)

= Highest OR value
= Lowest OR value

Supplementary data: Association between BMI at baseline and ASDAS-CII at 3 months

Model 1 ---------------------------- Crude (unadjusted) association

Exposure	BMI
Outcome	ASDAS-CII
Timepoint	3 months
N (countries)	12
N (patients)	2561

		ASDAS-CII No	ASDAS-CII Yes
BMI	Normal	521	590
	Overweight	469	440
	Obese	336	205

OR	95%CI-Lo	95%CI-Hi
0.83	0.69	0.99
0.54	0.44	0.66

Model 2 ---------------------------- Model 1, adjusted for: age, gender, country, calendar year, disease duration and disease activity

N (countries) 12
N (patients) 2446

Reference country = Switzerland (CH)
Blank line = No data for this analysis

	OR	95%CI-Lo	95%CI-Hi
BMI = Overweight	0.78	0.64	0.96
BMI = Obese	0.50	0.39	0.63
Age	0.99	0.98	1.00
Male	1.64	1.38	1.97
BASDAI	1.03	1.02	1.03
DiseaseDuration_2	0.70	0.54	0.91
DiseaseDuration_3	0.85	0.68	1.07
DiseaseDuration_4	0.86	0.66	1.11
DiseaseDuration_5	1.18	0.67	2.11
CalendarYear	1.00	0.97	1.04
Czech Republic (CZ)	2.86	1.43	6.04
Denmark (DK)	0.58	0.40	0.83
Spain (ES)			
Iceland (IS)	1.40	0.12	32.48
Italy (IT)	0.31	0.15	0.60
Netherlands (NL)	0.43	0.18	0.99
Norway (NO)			
Portugal (PT)	0.98	0.64	1.49
Romania (RO)	6.00	2.84	13.78
Finland (SF)	1.19	0.73	1.94
Slovenia (SI)	0.53	0.32	0.90
Turkey (TR)	1.63	1.07	2.47
United Kingdom (UK)	0.51	0.32	0.81

Model 3a/3b ---------------------- Model 2 additionally adjusted, in turn, for Smoking and Alcohol

Individual models. Result = OR (BMI) from each model

		OR (over-wt)	95%CI-Lo	95%CI-Hi	OR (ov)↑↓	OR (obese)	95%CI-Lo	95%CI-Hi	OR (ob)↑↓	N	N↓
(3a)	Smoking	0.75	0.61	0.92	0.032	0.47	0.37	0.60	0.024	2262	184 (8%)
(3b)	Alcohol	0.81	0.62	1.06	0.027	0.52	0.37	0.72	0.020	1216	1230 (50%)

Sensitivity analysis -------------- Model 2 additionally adjusted, in turn, for other variables shown

Individual models. Result = OR (BMI) from each model

	OR (over-wt)	95%CI-Lo	95%CI-Hi	OR (ov)↑↓	OR (obese)	95%CI-Lo	95%CI-Hi	OR (ob)↑↓	N	N↓
Yrs since symptom onset	0.74	0.59	0.92	0.050	0.47	0.36	0.62	0.024	1933	513 (21%)
HLA-B27 status	0.85	0.66	1.11	0.070	0.65	0.47	0.89	0.150	1427	1019 (42%)
ESR	0.84	0.59	1.20	0.058	0.66	0.44	1.00	0.165	1005	1441 (59%)
CRP	0.72	0.57	0.90	0.068	0.37	0.28	0.48	0.128	2346	100 (4%)
mNY criteria	0.91	0.69	1.21	0.126	0.65	0.47	0.92	0.159	1238	1208 (49%)
ASAS criteria	0.84	0.65	1.08	0.057	0.54	0.40	0.74	0.046	1495	951 (39%)
IBD	0.83	0.65	1.06	0.047	0.53	0.39	0.71	0.031	1672	774 (32%)
Uveitis	0.86	0.67	1.09	0.073	0.57	0.43	0.77	0.076	1648	798 (33%)
Psoriasis	0.91	0.72	1.16	0.127	0.54	0.40	0.72	0.041	1669	777 (32%)
Comorbidity cardiovascular	0.84	0.60	1.16	0.054	0.63	0.43	0.93	0.136	1003	1443 (59%)
Comorbidity kidney	0.79	0.56	1.11	0.002	0.62	0.41	0.92	0.120	918	1528 (62%)
Comorbidity diabetes	0.79	0.57	1.09	0.004	0.57	0.39	0.83	0.070	1019	1427 (58%)
BASFI	0.81	0.66	0.99	0.026	0.51	0.40	0.65	0.015	2302	144 (6%)
BASMI (3pt)	0.77	0.54	1.10	0.013	0.27	0.17	0.43	0.226	700	1746 (71%)
Patient global	0.77	0.63	0.94	0.012	0.49	0.38	0.63	0.004	2424	22 (1%)
Physician global	0.80	0.64	1.00	0.018	0.48	0.37	0.63	0.013	1953	493 (20%)
Pain (VAS)	0.72	0.58	0.89	0.062	0.44	0.34	0.57	0.052	2169	277 (11%)
Fatigue	0.77	0.63	0.94	0.013	0.50	0.39	0.64	0.002	2361	85 (3%)
HAQ	0.68	0.51	0.91	0.103	0.34	0.24	0.48	0.156	1181	1265 (52%)
csDMARD	0.76	0.56	1.03	0.023	0.49	0.35	0.70	0.003	1177	1269 (52%)
NSAID	0.88	0.63	1.24	0.100	0.67	0.44	1.01	0.173	843	1603 (66%)

(red) = Highest OR value
(blue) = Lowest OR value

Supplementary data: Association between BMI at baseline and ASDAS-MI at 3 months

Model 1 ---------------------------- Crude (unadjusted) association

Exposure	BMI
Outcome	ASDAS-MI
Timepoint	3 months
N (countries)	12
N (patients)	2561

		ASDAS-MI	
		No	Yes
BMI	Normal	754	357
	Overweight	677	232
	Obese	447	94

OR	95%CI-Lo	95%CI-Hi
0.72	0.60	0.88
0.44	0.34	0.57

Model 2 ---------------------------- Model 1, adjusted for: age, gender, country, calendar year, disease duration and disease activity

N (countries)	12
N (patients)	2446

Reference country = Switzerland (CH)
Blank line = No data for this analysis

	OR	95%CI-Lo	95%CI-Hi
BMI = Overweight	0.66	0.53	0.83
BMI = Obese	0.40	0.29	0.53
Age	0.97	0.96	0.98
Male	1.94	1.57	2.39
BASDAI	1.03	1.03	1.04
DiseaseDuration_2	0.73	0.54	0.99
DiseaseDuration_3	1.02	0.79	1.32
DiseaseDuration_4	0.97	0.71	1.31
DiseaseDuration_5	1.12	0.55	2.16
CalendarYear	1.01	0.97	1.05
Czech Republic (CZ)	3.19	1.65	6.25
Denmark (DK)	0.71	0.47	1.10
Spain (ES)			
Iceland (IS)	4.99	0.41	117.07
Italy (IT)	0.45	0.16	1.08
Netherlands (NL)	0.92	0.34	2.32
Norway (NO)			
Portugal (PT)	1.51	0.94	2.44
Romania (RO)	7.41	3.76	15.00
Finland (SF)	1.06	0.58	1.92
Slovenia (SI)	0.79	0.43	1.44
Turkey (TR)	1.80	1.11	2.96
United Kingdom (UK)	0.48	0.27	0.87

Model 3a/3b ---------------------- Model 2 additionally adjusted, in turn, for Smoking and Alcohol

Individual models. Result = OR (BMI) from each model

		OR (over-wt)	95%CI-Lo	95%CI-Hi	OR (ov) ↑↓	OR (obese)	95%CI-Lo	95%CI-Hi	OR (ob) ↑↓	N	N↓
(3a)	Smoking	0.65	0.52	0.82	0.009	0.40	0.29	0.54	0.003	2262	184 (8%)
(3b)	Alcohol	0.61	0.44	0.84	0.054	0.39	0.25	0.60	0.002	1216	1230 (50%)

Sensitivity analysis -------------- Model 2 additionally adjusted, in turn, for other variables shown

Individual models. Result = OR (BMI) from each model

	OR (over-wt)	95%CI-Lo	95%CI-Hi	OR (ov) ↑↓	OR (obese)	95%CI-Lo	95%CI-Hi	OR (ob) ↑↓	N	N↓
Yrs since symptom onset	0.65	0.51	0.83	0.013	0.41	0.30	0.57	0.015	1933	513 (21%)
HLA-B27 status	0.78	0.58	1.04	0.113	0.55	0.38	0.80	0.155	1427	1019 (42%)
ESR	0.61	0.41	0.90	0.050	0.40	0.24	0.65	0.004	1005	1441 (59%)
CRP	0.64	0.50	0.83	0.019	0.32	0.23	0.44	0.082	2346	100 (4%)
mNY criteria	0.75	0.54	1.03	0.084	0.55	0.37	0.83	0.156	1238	1208 (49%)
ASAS criteria	0.73	0.55	0.97	0.069	0.47	0.32	0.68	0.073	1495	951 (39%)
IBD	0.67	0.51	0.88	0.010	0.43	0.28	0.61	0.032	1672	774 (32%)
Uveitis	0.71	0.54	0.94	0.049	0.47	0.32	0.66	0.068	1648	798 (33%)
Psoriasis	0.73	0.56	0.96	0.071	0.45	0.31	0.64	0.051	1669	777 (32%)
Comorbidity cardiovascular	0.63	0.44	0.91	0.027	0.36	0.22	0.57	0.036	1003	1443 (59%)
Comorbidity kidney	0.64	0.43	0.93	0.027	0.36	0.22	0.58	0.035	918	1528 (62%)
Comorbidity diabetes	0.62	0.43	0.89	0.039	0.33	0.20	0.52	0.072	1019	1427 (58%)
BASFI	0.65	0.51	0.82	0.015	0.38	0.28	0.52	0.017	2302	144 (6%)
BASMI (3pt)	0.80	0.54	1.20	0.143	0.31	0.16	0.55	0.090	700	1746 (71%)
Patient global	0.65	0.51	0.81	0.015	0.39	0.29	0.53	0.006	2424	22 (1%)
Physician global	0.69	0.53	0.88	0.027	0.41	0.29	0.57	0.013	1953	493 (20%)
Pain (VAS)	0.63	0.49	0.80	0.033	0.36	0.26	0.49	0.037	2169	277 (11%)
Fatigue	0.66	0.53	0.83	0.002	0.39	0.29	0.53	0.006	2361	85 (3%)
HAQ	0.72	0.52	0.99	0.057	0.34	0.22	0.53	0.053	1181	1265 (52%)
csDMARD	0.66	0.47	0.93	0.001	0.44	0.28	0.66	0.041	1177	1269 (52%)
NSAID	0.65	0.44	0.94	0.015	0.46	0.28	0.75	0.061	843	1603 (66%)

= Highest OR value
= Lowest OR value

Supplementary data: Association between BMI at baseline and ASAS-20 at 3 months

Model 1 ---------------------------- Crude (unadjusted) association

Exposure	BMI
Outcome	ASAS-20
Timepoint	3 months
N (countries)	12
N (patients)	3313

		ASAS-20	
		No	Yes
BMI	Normal	845	546
	Overweight	795	405
	Obese	515	207

OR	95%CI-Lo	95%CI-Hi
0.79	0.67	0.93
0.62	0.51	0.75

Model 2 ---------------------------- Model 1, adjusted for: age, gender, country, calendar year, disease duration and disease activity

N (countries)	10
N (patients)	2838

Reference country = Switzerland (CH)
Blank line = No data for this analysis
Rounding to 2dp. Actual value = non-zero.

	OR	95%CI-Lo	95%CI-Hi
BMI = Overweight	0.77	0.64	0.92
BMI = Obese	0.58	0.46	0.73
Age	0.99	0.98	0.99
Male	1.12	0.95	1.33
BASDAI	1.02	1.02	1.02
DiseaseDuration_2	0.91	0.72	1.17
DiseaseDuration_3	0.99	0.80	1.22
DiseaseDuration_4	0.92	0.72	1.18
DiseaseDuration_5	1.01	0.55	1.87
CalendarYear	1.01	0.98	1.05
Czech Republic (CZ)	2.11	1.12	4.14
Denmark (DK)	0.63	0.46	0.86
Spain (ES)			
Iceland (IS)	1.24	0.20	9.76
Italy (IT)			
Netherlands (NL)	0.21	0.09	0.46
Norway (NO)			
Portugal (PT)	0.00	0.00	0.02
Romania (RO)			
Finland (SF)	0.60	0.40	0.89
Slovenia (SI)	0.30	0.19	0.46
Turkey (TR)	1.36	0.94	1.99
United Kingdom (UK)	0.60	0.40	0.90

Model 3a/3b ---------------------- Model 2 additionally adjusted, in turn, for Smoking and Alcohol

Individual models. Result = OR (BMI) from each model

		OR (over-wt)	95%CI-Lo	95%CI-Hi	OR (ov)↑↓	OR (obese)	95%CI-Lo	95%CI-Hi	OR (ob)↑↓	N	N↓
(3a)	Smoking	0.77	0.63	0.93	0.002	0.56	0.44	0.71	0.019	2546	292 (10%)
(3b)	Alcohol	0.81	0.62	1.05	0.039	0.60	0.43	0.82	0.018	1332	1506 (53%)

Sensitivity analysis -------------- Model 2 additionally adjusted, in turn, for other variables shown

Individual models. Result = OR (BMI) from each model

	OR (over-wt)	95%CI-Lo	95%CI-Hi	OR (ov)↑↓	OR (obese)	95%CI-Lo	95%CI-Hi	OR (ob)↑↓	N	N↓
Yrs since symptom onset	0.75	0.61	0.93	0.013	0.55	0.42	0.71	0.034	1996	842 (30%)
HLA-B27 status	0.84	0.65	1.09	0.079	0.73	0.54	0.99	0.155	1532	1306 (46%)
ESR	0.64	0.44	0.93	0.123	0.80	0.52	1.23	0.218	1041	1797 (63%)
CRP	0.76	0.62	0.93	0.008	0.51	0.40	0.65	0.071	2379	459 (16%)
mNY criteria	0.93	0.70	1.23	0.159	0.66	0.47	0.93	0.085	1266	1572 (55%)
ASAS criteria	0.84	0.65	1.07	0.069	0.64	0.48	0.85	0.058	1553	1285 (45%)
IBD	0.77	0.61	0.97	0.004	0.62	0.47	0.82	0.041	1854	984 (35%)
Uveitis	0.82	0.65	1.03	0.054	0.68	0.52	0.89	0.099	1809	1029 (36%)
Psoriasis	0.83	0.66	1.04	0.061	0.63	0.48	0.83	0.050	1844	994 (35%)
Comorbidity cardiovascular	0.80	0.58	1.12	0.039	0.87	0.60	1.26	0.292	1061	1777 (63%)
Comorbidity kidney	0.74	0.52	1.04	0.027	0.84	0.56	1.24	0.259	955	1883 (66%)
Comorbidity diabetes	0.81	0.59	1.12	0.047	0.85	0.59	1.23	0.274	1083	1755 (62%)
BASFI	0.78	0.65	0.94	0.013	0.59	0.47	0.74	0.013	2798	40 (1%)
BASMI (3pt)	0.78	0.54	1.11	0.010	0.44	0.28	0.68	0.140	716	2122 (75%)
Patient global	0.77	0.64	0.93	0.006	0.57	0.45	0.71	0.012	2691	147 (5%)
Physician global	0.76	0.62	0.93	0.005	0.53	0.41	0.68	0.052	2314	524 (18%)
Pain (VAS)	0.77	0.64	0.93	0.005	0.57	0.45	0.72	0.009	2685	153 (5%)
Fatigue	0.77	0.64	0.93	0.005	0.59	0.47	0.73	0.007	2650	188 (7%)
HAQ	0.87	0.67	1.14	0.109	0.61	0.44	0.84	0.030	1479	1359 (48%)
csDMARD	0.87	0.65	1.16	0.106	0.63	0.45	0.88	0.046	1371	1467 (52%)
NSAID	0.70	0.49	0.99	0.070	0.61	0.40	0.93	0.031	831	2007 (71%)

[red] = Highest OR value
[blue] = Lowest OR value

Supplementary data: Association between BMI at baseline and ASAS-40 at 3 months

Model 1 ---------------------------- Crude (unadjusted) association

Exposure	BMI
Outcome	ASAS-40
Timepoint	3 months
N (countries)	12
N (patients)	3768

		ASAS-40	
		No	Yes
BMI	Normal	1198	384
	Overweight	1110	251
	Obese	709	116

OR	95%CI-Lo	95%CI-Hi
0.71	0.59	0.84
0.51	0.41	0.64

Model 2 ---------------------------- Model 1, adjusted for: age, gender, country, calendar year, disease duration and disease activity

N (countries) 9
N (patients) 3122

Reference country = Switzerland (CH)
Blank line = No data for this analysis

	OR	95%CI-Lo	95%CI-Hi
BMI = Overweight	0.67	0.55	0.82
BMI = Obese	0.48	0.37	0.61
Age	0.98	0.97	0.99
Male	1.26	1.05	1.51
BASDAI	1.02	1.02	1.03
DiseaseDuration_2	0.91	0.70	1.18
DiseaseDuration_3	0.91	0.72	1.15
DiseaseDuration_4	0.82	0.62	1.07
DiseaseDuration_5	0.71	0.34	1.39
CalendarYear	1.03	0.99	1.06
Czech Republic (CZ)	1.39	0.77	2.53
Denmark (DK)	0.53	0.38	0.74
Spain (ES)			
Iceland (IS)	2.49	0.39	19.77
Italy (IT)			
Netherlands (NL)	0.33	0.13	0.73
Norway (NO)			
Portugal (PT)			
Romania (RO)			
Finland (SF)	0.47	0.30	0.73
Slovenia (SI)	0.21	0.13	0.34
Turkey (TR)	1.30	0.88	1.92
United Kingdom (UK)	0.52	0.34	0.81

Model 3a/3b ---------------------- Model 2 additionally adjusted, in turn, for Smoking and Alcohol

Individual models. Result = OR (BMI) from each model

		OR (over-wt)	95%CI-Lo	95%CI-Hi	OR (ov) ↑↓	OR (obese)	95%CI-Lo	95%CI-Hi	OR (ob) ↑↓	N	N↓
(3a)	Smoking	0.67	0.54	0.82	0.002	0.46	0.35	0.60	0.015	2758	364 (12%)
(3b)	Alcohol	0.66	0.49	0.88	0.015	0.53	0.36	0.75	0.049	1404	1718 (55%)

Sensitivity analysis -------------- Model 2 additionally adjusted, in turn, for other variables shown

Individual models. Result = OR (BMI) from each model

	OR (over-wt)	95%CI-Lo	95%CI-Hi	OR (ov) ↑↓	OR (obese)	95%CI-Lo	95%CI-Hi	OR (ob) ↑↓	N	N↓
Yrs since symptom onset	0.64	0.51	0.80	0.034	0.46	0.34	0.61	0.021	2090	1032 (33%)
HLA-B27 status	0.83	0.63	1.09	0.158	0.69	0.49	0.96	0.211	1710	1412 (45%)
ESR	0.51	0.34	0.76	0.162	0.64	0.40	1.01	0.160	1284	1838 (59%)
CRP	0.64	0.52	0.80	0.027	0.41	0.31	0.54	0.063	2566	556 (18%)
mNY criteria	0.86	0.63	1.17	0.192	0.70	0.48	1.02	0.225	1439	1683 (54%)
ASAS criteria	0.79	0.61	1.03	0.119	0.60	0.43	0.83	0.126	1710	1412 (45%)
IBD	0.71	0.56	0.91	0.043	0.55	0.40	0.75	0.075	2057	1065 (34%)
Uveitis	0.75	0.59	0.97	0.083	0.61	0.44	0.82	0.129	2006	1116 (36%)
Psoriasis	0.76	0.60	0.98	0.094	0.55	0.40	0.75	0.070	2041	1081 (35%)
Comorbidity cardiovascular	0.62	0.44	0.89	0.046	0.60	0.39	0.90	0.120	1254	1868 (60%)
Comorbidity kidney	0.60	0.41	0.88	0.068	0.65	0.41	1.00	0.168	1143	1979 (63%)
Comorbidity diabetes	0.63	0.44	0.90	0.036	0.66	0.43	0.99	0.179	1274	1848 (59%)
BASFI	0.67	0.55	0.83	0.003	0.48	0.37	0.62	0.003	3057	65 (2%)
BASMI (3pt)	0.74	0.51	1.09	0.071	0.25	0.14	0.43	0.229	753	2369 (76%)
Patient global	0.66	0.54	0.82	0.006	0.47	0.36	0.61	0.007	2872	250 (8%)
Physician global	0.67	0.54	0.83	0.003	0.43	0.32	0.57	0.047	2444	678 (22%)
Pain (VAS)	0.67	0.55	0.82	0.001	0.47	0.37	0.61	0.005	2870	252 (8%)
Fatigue	0.67	0.55	0.82	0.003	0.48	0.37	0.62	0.003	2858	264 (8%)
HAQ	0.75	0.57	0.98	0.077	0.42	0.34	0.59	0.060	1618	1504 (48%)
csDMARD	0.77	0.56	1.04	0.096	0.54	0.37	0.79	0.066	1541	1581 (51%)
NSAID	0.52	0.35	0.75	0.154	0.51	0.33	0.80	0.036	902	2220 (71%)

= Highest OR value
= Lowest OR value

Supplementary data: Association between BMI at baseline and ASAS-5of6 at 3 months

Model 1 ---------------------------- Crude (unadjusted) association

Exposure	BMI
Outcome	ASAS-5of6
Timepoint	3 months
N (countries)	12
N (patients)	2797

		ASAS-5of6	
		No	Yes
BMI	Normal	838	318
	Overweight	805	216
	Obese	529	91

OR	95%CI-Lo	95%CI-Hi
0.71	0.58	0.86
0.45	0.35	0.58

Model 2 ---------------------------- Model 1, adjusted for: age, gender, country, calendar year, disease duration and disease activity

N (countries) 11
N (patients) 2325

Reference country = Switzerland (CH)
Blank line = No data for this analysis

	OR	95%CI-Lo	95%CI-Hi
BMI = Overweight	0.70	0.56	0.88
BMI = Obese	0.45	0.33	0.59
Age	0.98	0.97	0.99
Male	1.46	1.19	1.80
BASDAI	1.01	1.01	1.02
DiseaseDuration_2	0.82	0.61	1.11
DiseaseDuration_3	1.05	0.81	1.36
DiseaseDuration_4	0.82	0.60	1.12
DiseaseDuration_5	0.88	0.39	1.89
CalendarYear	1.02	0.98	1.06
Czech Republic (CZ)	3.94	2.00	8.01
Denmark (DK)	0.72	0.48	1.08
Spain (ES)			
Iceland (IS)	2.97	0.46	23.56
Italy (IT)			
Netherlands (NL)	0.34	0.12	0.85
Norway (NO)			
Portugal (PT)	0.08	0.02	0.22
Romania (RO)	0.83	0.24	2.61
Finland (SF)	0.82	0.49	1.38
Slovenia (SI)	0.38	0.24	0.63
Turkey (TR)	2.20	1.39	3.51
United Kingdom (UK)	0.57	0.33	0.99

Model 3a/3b ---------------------- Model 2 additionally adjusted, in turn, for Smoking and Alcohol

Individual models. Result = OR (BMI) from each model

		OR (over-wt)	95%CI-Lo	95%CI-Hi	OR (ov)↑↓	OR (obese)	95%CI-Lo	95%CI-Hi	OR (ob)↑↓	N	N↓
(3a)	Smoking	0.69	0.55	0.87	0.010	0.42	0.31	0.56	0.028	2080	245 (11%)
(3b)	Alcohol	0.73	0.52	1.02	0.032	0.47	0.31	0.72	0.027	1033	1292 (56%)

Sensitivity analysis -------------- Model 2 additionally adjusted, in turn, for other variables shown

Individual models. Result = OR (BMI) from each model

	OR (over-wt)	95%CI-Lo	95%CI-Hi	OR (ov)↑↓	OR (obese)	95%CI-Lo	95%CI-Hi	OR (ob)↑↓	N	N↓
Yrs since symptom onset	0.68	0.52	0.88	0.024	0.40	0.29	0.56	0.044	1542	783 (34%)
HLA-B27 status	0.75	0.55	1.01	0.048	0.64	0.44	0.93	0.194	1322	1003 (43%)
ESR	0.79	0.52	1.20	0.088	0.57	0.33	0.94	0.119	1014	1311 (56%)
CRP	0.72	0.56	0.93	0.023	0.38	0.28	0.52	0.068	2028	297 (13%)
mNY criteria	0.98	0.70	1.34	0.284	0.64	0.42	0.96	0.191	1148	1177 (51%)
ASAS criteria	0.82	0.61	1.10	0.118	0.53	0.36	0.76	0.080	1361	964 (41%)
IBD	0.71	0.54	0.93	0.008	0.52	0.36	0.73	0.073	1580	745 (32%)
Uveitis	0.76	0.57	1.00	0.056	0.55	0.38	0.77	0.099	1545	780 (34%)
Psoriasis	0.77	0.58	1.01	0.069	0.51	0.36	0.72	0.064	1577	748 (32%)
Comorbidity cardiovascular	0.84	0.57	1.22	0.137	0.68	0.42	1.08	0.235	984	1341 (58%)
Comorbidity kidney	0.81	0.54	1.20	0.108	0.73	0.44	1.18	0.281	905	1420 (61%)
Comorbidity diabetes	0.84	0.58	1.22	0.139	0.68	0.43	1.07	0.231	1005	1320 (57%)
BASFI	0.72	0.57	0.90	0.017	0.46	0.34	0.61	0.012	2274	51 (2%)
BASMI (3pt)	0.80	0.52	1.23	0.102	0.29	0.16	0.52	0.153	571	1754 (75%)
Patient global	0.70	0.55	0.88	0.000	0.42	0.31	0.57	0.024	2112	213 (9%)
Physician global	0.69	0.54	0.89	0.007	0.41	0.29	0.56	0.041	1815	510 (22%)
Pain (VAS)	0.71	0.56	0.89	0.006	0.44	0.32	0.58	0.011	2175	150 (6%)
Fatigue	0.71	0.56	0.89	0.006	0.45	0.34	0.60	0.006	2171	154 (7%)
HAQ	0.75	0.56	1.02	0.054	0.38	0.25	0.47	0.070	1250	1075 (46%)
csDMARD	0.79	0.56	1.12	0.093	0.50	0.32	0.77	0.049	1081	1244 (54%)
NSAID	0.73	0.48	1.13	0.034	0.55	0.32	0.94	0.106	658	1667 (72%)

= Highest OR value
= Lowest OR value

Supplementary data: Association between BMI at baseline and BASDAI-50 at 12 months

Model 1 ---------------------------- Crude (unadjusted) association

Exposure	BMI
Outcome	BASDAI-50
Timepoint	12 months
N (countries)	13
N (patients)	5017

		BASDAI-50	
		No	Yes
BMI	Normal	870	1282
	Overweight	886	992
	Obese	556	431

OR	95%CI-Lo	95%CI-Hi
0.76	0.67	0.86
0.53	0.45	0.61

Model 2 ---------------------------- Model 1, adjusted for: age, gender, country, calendar year, disease duration and disease activity

N (countries)	13
N (patients)	5010

Reference country = Switzerland (CH)
Blank line = No data for this analysis

	OR	95%CI-Lo	95%CI-Hi
BMI = Overweight	0.76	0.66	0.87
BMI = Obese	0.53	0.45	0.63
Age	0.97	0.96	0.98
Male	1.53	1.35	1.74
BASDAI	1.01	1.01	1.01
DiseaseDuration_2	0.89	0.74	1.07
DiseaseDuration_3	0.99	0.84	1.16
DiseaseDuration_4	1.03	0.87	1.23
DiseaseDuration_5	0.94	0.61	1.45
CalendarYear	1.00	0.98	1.02
Czech Republic (CZ)	2.77	2.16	3.58
Denmark (DK)	0.68	0.53	0.87
Spain (ES)	0.81	0.57	1.15
Iceland (IS)	1.38	0.35	5.75
Italy (IT)	1.08	0.83	1.41
Netherlands (NL)	0.65	0.34	1.21
Norway (NO)			
Portugal (PT)	1.19	0.85	1.68
Romania (RO)	10.14	5.73	19.08
Finland (SF)	1.30	0.95	1.79
Slovenia (SI)	1.47	1.09	1.98
Turkey (TR)	2.93	2.11	4.09
United Kingdom (UK)	0.90	0.60	1.36

Model 3a/3b ---------------------- Model 2 additionally adjusted, in turn, for Smoking and Alcohol

Individual models. Result = OR (BMI) from each model

		OR (over-wt)	95%CI-Lo	95%CI-Hi	OR (ov)↑↓	OR (obese)	95%CI-Lo	95%CI-Hi	OR (ob)↑↓	N	N↓
(3a)	Smoking	0.75	0.65	0.87	0.010	0.51	0.43	0.61	0.024	4409	601 (12%)
(3b)	Alcohol	0.63	0.48	0.81	0.135	0.42	0.30	0.59	0.113	1267	3743 (75%)

Sensitivity analysis -------------- Model 2 additionally adjusted, in turn, for other variables shown

Individual models. Result = OR (BMI) from each model

	OR (over-wt)	95%CI-Lo	95%CI-Hi	OR (ov)↑↓	OR (obese)	95%CI-Lo	95%CI-Hi	OR (ob)↑↓	N	N↓
Yrs since symptom onset	0.75	0.63	0.90	0.006	0.55	0.44	0.68	0.013	3198	1812 (36%)
HLA-B27 status	0.76	0.64	0.90	0.001	0.54	0.44	0.66	0.006	3407	1603 (32%)
ESR	0.77	0.64	0.92	0.009	0.53	0.43	0.66	0.004	3240	1770 (35%)
CRP	0.71	0.61	0.84	0.046	0.48	0.39	0.58	0.059	3877	1133 (23%)
mNY criteria	0.80	0.64	1.00	0.039	0.46	0.35	0.61	0.076	1821	3189 (64%)
ASAS criteria	0.76	0.64	0.92	0.004	0.52	0.41	0.64	0.019	2964	2046 (41%)
IBD	0.75	0.63	0.89	0.010	0.53	0.43	0.65	0.005	3480	1530 (31%)
Uveitis	0.76	0.65	0.90	0.000	0.54	0.44	0.66	0.004	3663	1347 (27%)
Psoriasis	0.77	0.65	0.91	0.005	0.54	0.44	0.67	0.010	3416	1594 (32%)
Comorbidity cardiovascular	0.85	0.70	1.03	0.090	0.60	0.48	0.75	0.066	2935	2075 (41%)
Comorbidity kidney	0.80	0.66	0.97	0.040	0.58	0.46	0.73	0.047	2822	2188 (44%)
Comorbidity diabetes	0.79	0.66	0.96	0.032	0.62	0.49	0.77	0.081	2940	2070 (41%)
BASFI	0.74	0.64	0.86	0.017	0.56	0.47	0.67	0.028	4512	498 (10%)
BASMI (3pt)	0.65	0.46	0.92	0.111	0.33	0.20	0.52	0.207	714	4296 (86%)
Patient global	0.70	0.60	0.81	0.062	0.51	0.42	0.62	0.025	4024	986 (20%)
Physician global	0.71	0.60	0.83	0.055	0.52	0.42	0.63	0.018	3512	1498 (30%)
Pain (VAS)	0.72	0.62	0.84	0.042	0.50	0.42	0.61	0.033	4018	992 (20%)
Fatigue	0.73	0.63	0.86	0.026	0.52	0.43	0.63	0.013	4163	847 (17%)
HAQ	0.89	0.73	1.08	0.128	0.64	0.50	0.80	0.101	2716	2294 (46%)
csDMARD	0.78	0.64	0.94	0.015	0.52	0.41	0.65	0.018	2757	2253 (45%)
NSAID	0.77	0.62	0.96	0.012	0.61	0.47	0.79	0.075	2170	2840 (57%)

= Highest OR value
= Lowest OR value

Supplementary data: Association between BMI at baseline and ASDAS-Inactive at 12 months

Model 1 ---------------------------- Crude (unadjusted) association

Exposure	BMI
Outcome	ASDAS-Inactive
Timepoint	12 months
N (countries)	12
N (patients)	4875

		ASDAS-Inactive	
		No	Yes
BMI	Normal	1311	772
	Overweight	1287	500
	Obese	826	179

OR	95%CI-Lo	95%CI-Hi
0.66	0.58	0.76
0.37	0.31	0.44

Model 2 ---------------------------- Model 1, adjusted for: age, gender, country, calendar year, disease duration and disease activity

N (countries)	11
N (patients)	3398

Reference country = Switzerland (CH)
Blank line = No data for this analysis

	OR	95%CI-Lo	95%CI-Hi
BMI = Overweight	0.73	0.61	0.87
BMI = Obese	0.43	0.34	0.55
Age	0.96	0.95	0.97
Male	1.59	1.33	1.89
BASDAI	0.98	0.98	0.98
DiseaseDuration_2	1.01	0.79	1.29
DiseaseDuration_3	1.18	0.95	1.45
DiseaseDuration_4	1.20	0.95	1.52
DiseaseDuration_5	0.77	0.43	1.34
CalendarYear	1.05	1.01	1.08
Czech Republic (CZ)	1.64	1.20	2.27
Denmark (DK)	0.89	0.65	1.23
Spain (ES)			
Iceland (IS)			
Italy (IT)	1.92	1.14	3.20
Netherlands (NL)	0.62	0.19	1.65
Norway (NO)			
Portugal (PT)	1.17	0.76	1.78
Romania (RO)	2.39	1.46	3.90
Finland (SF)	1.45	0.95	2.23
Slovenia (SI)	1.09	0.57	1.97
Turkey (TR)	1.52	1.04	2.24
United Kingdom (UK)	1.12	0.65	1.88

Model 3a/3b ---------------------- Model 2 additionally adjusted, in turn, for Smoking and Alcohol

Individual models. Result = OR (BMI) from each model

		OR (over-wt)	95%CI-Lo	95%CI-Hi	OR (ov)↑↓	OR (obese)	95%CI-Lo	95%CI-Hi	OR (ob)↑↓	N	N↓
(3a)	Smoking	0.71	0.59	0.86	0.015	0.42	0.33	0.54	0.011	3149	249 (7%)
(3b)	Alcohol	0.73	0.52	1.02	0.005	0.41	0.24	0.69	0.021	1098	2300 (68%)

Sensitivity analysis -------------- Model 2 additionally adjusted, in turn, for other variables shown

Individual models. Result = OR (BMI) from each model

	OR (over-wt)	95%CI-Lo	95%CI-Hi	OR (ov)↑↓	OR (obese)	95%CI-Lo	95%CI-Hi	OR (ob)↑↓	N	N↓
Yrs since symptom onset	0.69	0.56	0.83	0.042	0.43	0.33	0.56	0.007	2776	622 (18%)
HLA-B27 status	0.77	0.63	0.96	0.047	0.49	0.37	0.64	0.052	2434	964 (28%)
ESR	0.75	0.59	0.95	0.023	0.49	0.36	0.66	0.052	2012	1386 (41%)
CRP	0.74	0.61	0.89	0.008	0.43	0.34	0.56	0.001	3083	315 (9%)
mNY criteria	0.77	0.57	1.03	0.041	0.35	0.22	0.54	0.087	1326	2072 (61%)
ASAS criteria	0.77	0.62	0.96	0.044	0.49	0.36	0.65	0.051	2332	1066 (31%)
IBD	0.80	0.65	0.98	0.070	0.49	0.37	0.64	0.053	2629	769 (23%)
Uveitis	0.78	0.63	0.95	0.049	0.47	0.36	0.61	0.034	2615	783 (23%)
Psoriasis	0.77	0.62	0.94	0.038	0.48	0.36	0.63	0.046	2610	788 (23%)
Comorbidity cardiovascular	0.74	0.58	0.93	0.009	0.51	0.38	0.69	0.076	1988	1410 (41%)
Comorbidity kidney	0.75	0.59	0.96	0.027	0.48	0.35	0.66	0.050	1867	1531 (45%)
Comorbidity diabetes	0.72	0.57	0.91	0.006	0.47	0.34	0.63	0.033	1994	1404 (41%)
BASFI	0.71	0.59	0.86	0.018	0.51	0.39	0.65	0.070	3193	205 (6%)
BASMI (3pt)	0.63	0.40	0.97	0.103	0.40	0.20	0.77	0.032	636	2762 (81%)
Patient global	0.74	0.62	0.89	0.015	0.46	0.36	0.59	0.027	3252	146 (4%)
Physician global	0.76	0.63	0.93	0.036	0.49	0.37	0.63	0.050	2800	598 (18%)
Pain (VAS)	0.76	0.62	0.92	0.035	0.46	0.36	0.59	0.024	3077	321 (9%)
Fatigue	0.71	0.59	0.85	0.019	0.43	0.34	0.55	0.002	3280	118 (3%)
HAQ	0.81	0.64	1.02	0.078	0.54	0.40	0.72	0.101	1919	1479 (44%)
csDMARD	0.74	0.57	0.96	0.013	0.45	0.32	0.62	0.014	1726	1672 (49%)
NSAID	0.65	0.51	0.84	0.073	0.55	0.40	0.76	0.118	1761	1637 (48%)

= Highest OR value
= Lowest OR value

Supplementary data: Association between BMI at baseline and ASDAS-LDA at 12 months

Model 1 ---------------------------- Crude (unadjusted) association

Exposure	BMI
Outcome	ASDAS-LDA
Timepoint	12 months
N (countries)	12
N (patients)	4875

		ASDAS-LDA	
		No	Yes
BMI	Normal	628	1455
	Overweight	659	1128
	Obese	524	481

OR	95%CI-Lo	95%CI-Hi
0.74	0.65	0.84
0.40	0.34	0.46

Model 2 ---------------------------- Model 1, adjusted for: age, gender, country, calendar year, disease duration and disease activity

N (countries)	12
N (patients)	3398

Reference country = Switzerland (CH)
Blank line = No data for this analysis

	OR	95%CI-Lo	95%CI-Hi
BMI = Overweight	0.76	0.64	0.91
BMI = Obese	0.40	0.32	0.49
Age	0.97	0.97	0.98
Male	1.35	1.15	1.59
BASDAI	0.97	0.97	0.98
DiseaseDuration_2	0.85	0.67	1.08
DiseaseDuration_3	0.92	0.75	1.14
DiseaseDuration_4	1.02	0.81	1.27
DiseaseDuration_5	0.76	0.45	1.28
CalendarYear	1.02	0.99	1.05
Czech Republic (CZ)	2.35	1.73	3.18
Denmark (DK)	0.71	0.53	0.94
Spain (ES)			
Iceland (IS)	0.55	0.11	2.43
Italy (IT)	1.01	0.63	1.64
Netherlands (NL)	0.62	0.27	1.37
Norway (NO)			
Portugal (PT)	1.13	0.78	1.66
Romania (RO)	7.16	4.07	13.06
Finland (SF)	1.40	0.91	2.18
Slovenia (SI)	1.06	0.65	1.74
Turkey (TR)	1.30	0.89	1.89
United Kingdom (UK)	0.97	0.61	1.53

Model 3a/3b ---------------------- Model 2 additionally adjusted, in turn, for Smoking and Alcohol

Individual models. Result = OR (BMI) from each model

		OR (over-wt)	95%CI-Lo	95%CI-Hi	OR (ov)↑↓	OR (obese)	95%CI-Lo	95%CI-Hi	OR (ob)↑↓	N	N↓
(3a)	Smoking	0.76	0.63	0.92	0.001	0.39	0.32	0.49	0.005	3149	249 (7%)
(3b)	Alcohol	0.67	0.50	0.90	0.095	0.40	0.27	0.59	0.003	1098	2300 (68%)

Sensitivity analysis -------------- Model 2 additionally adjusted, in turn, for other variables shown

Individual models. Result = OR (BMI) from each model

	OR (over-wt)	95%CI-Lo	95%CI-Hi	OR (ov)↑↓	OR (obese)	95%CI-Lo	95%CI-Hi	OR (ob)↑↓	N	N↓
Yrs since symptom onset	0.83	0.68	1.00	0.064	0.44	0.35	0.55	0.043	2776	622 (18%)
HLA-B27 status	0.79	0.64	0.98	0.027	0.38	0.30	0.49	0.014	2434	964 (28%)
ESR	0.78	0.61	0.99	0.014	0.40	0.30	0.52	0.001	2012	1386 (41%)
CRP	0.80	0.67	0.97	0.041	0.41	0.33	0.51	0.013	3083	315 (9%)
mNY criteria	0.87	0.66	1.14	0.106	0.43	0.30	0.59	0.029	1326	2072 (61%)
ASAS criteria	0.80	0.64	0.98	0.032	0.41	0.32	0.53	0.015	2332	1066 (31%)
IBD	0.78	0.64	0.95	0.016	0.41	0.32	0.52	0.011	2629	769 (23%)
Uveitis	0.79	0.65	0.97	0.029	0.41	0.33	0.52	0.017	2615	783 (23%)
Psoriasis	0.79	0.65	0.97	0.029	0.42	0.33	0.53	0.019	2610	788 (23%)
Comorbidity cardiovascular	0.87	0.68	1.11	0.105	0.46	0.35	0.60	0.061	1988	1410 (41%)
Comorbidity kidney	0.88	0.68	1.13	0.114	0.44	0.33	0.59	0.047	1867	1531 (45%)
Comorbidity diabetes	0.83	0.65	1.06	0.068	0.47	0.36	0.62	0.077	1994	1404 (41%)
BASFI	0.76	0.64	0.91	0.001	0.43	0.35	0.54	0.037	3193	205 (6%)
BASMI (3pt)	0.78	0.53	1.16	0.020	0.35	0.21	0.58	0.049	636	2762 (81%)
Patient global	0.76	0.63	0.91	0.005	0.41	0.33	0.50	0.009	3252	146 (4%)
Physician global	0.77	0.63	0.93	0.006	0.39	0.31	0.49	0.009	2800	598 (18%)
Pain (VAS)	0.75	0.63	0.91	0.010	0.39	0.32	0.49	0.003	3077	321 (9%)
Fatigue	0.77	0.65	0.93	0.010	0.41	0.33	0.51	0.015	3280	118 (3%)
HAQ	0.80	0.63	1.01	0.033	0.42	0.32	0.56	0.027	1919	1479 (44%)
csDMARD	0.80	0.62	1.04	0.041	0.39	0.29	0.52	0.003	1726	1672 (49%)
NSAID	0.80	0.62	1.02	0.035	0.48	0.36	0.63	0.080	1761	1637 (48%)

= Highest OR value
= Lowest OR value

Supplementary data: Association between BMI at baseline and ASDAS-CII at 12 months

Model 1 ---------------------------- Crude (unadjusted) association

Exposure	BMI
Outcome	ASDAS-CII
Timepoint	12 months
N (countries)	12
N (patients)	3160

		ASDAS-CII No	ASDAS-CII Yes
BMI	Normal	466	900
	Overweight	406	749
	Obese	278	361

OR	95%CI-Lo	95%CI-Hi
0.96	0.81	1.13
0.67	0.55	0.82

Model 2 ---------------------------- Model 1, adjusted for: age, gender, country, calendar year, disease duration and disease activity

N (countries) 12
N (patients) 3063

Reference country = Switzerland (CH)
Blank line = No data for this analysis

	OR	95%CI-Lo	95%CI-Hi
BMI = Overweight	0.92	0.75	1.12
BMI = Obese	0.52	0.41	0.66
Age	0.97	0.97	0.98
Male	1.48	1.23	1.77
BASDAI	1.03	1.03	1.04
DiseaseDuration_2	0.74	0.57	0.96
DiseaseDuration_3	0.93	0.74	1.17
DiseaseDuration_4	1.09	0.85	1.41
DiseaseDuration_5	0.72	0.40	1.30
CalendarYear	0.96	0.93	1.00
Czech Republic (CZ)	5.33	3.79	7.52
Denmark (DK)	0.74	0.55	1.00
Spain (ES)			
Iceland (IS)	0.55	0.09	3.39
Italy (IT)	1.45	0.79	2.64
Netherlands (NL)	0.23	0.09	0.56
Norway (NO)			
Portugal (PT)	1.56	1.05	2.33
Romania (RO)	10.07	5.16	21.13
Finland (SF)	0.97	0.61	1.54
Slovenia (SI)	0.94	0.55	1.62
Turkey (TR)	2.35	1.60	3.47
United Kingdom (UK)	1.08	0.66	1.78

Model 3a/3b ---------------------- Model 2 additionally adjusted, in turn, for Smoking and Alcohol

Individual models. Result = OR (BMI) from each model

		OR (over-wt)	95%CI-Lo	95%CI-Hi	OR (ov) ↑↓	OR (obese)	95%CI-Lo	95%CI-Hi	OR (ob) ↑↓	N	N↓
(3a)	Smoking	0.89	0.73	1.10	0.023	0.52	0.40	0.66	0.007	2864	199 (6%)
(3b)	Alcohol	0.83	0.61	1.12	0.090	0.49	0.33	0.71	0.036	976	2087 (68%)

Sensitivity analysis -------------- Model 2 additionally adjusted, in turn, for other variables shown

Individual models. Result = OR (BMI) from each model

	OR (over-wt)	95%CI-Lo	95%CI-Hi	OR (ov) ↑↓	OR (obese)	95%CI-Lo	95%CI-Hi	OR (ob) ↑↓	N	N↓
Yrs since symptom onset	0.91	0.73	1.13	0.011	0.51	0.40	0.67	0.008	2593	470 (15%)
HLA-B27 status	1.02	0.79	1.30	0.101	0.57	0.43	0.76	0.046	2220	843 (28%)
ESR	1.08	0.80	1.46	0.166	0.54	0.39	0.76	0.019	1872	1191 (39%)
CRP	0.85	0.68	1.05	0.069	0.40	0.31	0.52	0.123	2971	92 (3%)
mNY criteria	1.04	0.77	1.40	0.122	0.68	0.48	0.98	0.162	1207	1856 (61%)
ASAS criteria	0.96	0.75	1.22	0.041	0.56	0.44	0.75	0.040	2155	908 (30%)
IBD	0.98	0.78	1.24	0.067	0.56	0.42	0.73	0.035	2392	671 (22%)
Uveitis	1.01	0.80	1.28	0.092	0.58	0.44	0.76	0.056	2387	676 (22%)
Psoriasis	1.04	0.82	1.32	0.124	0.58	0.44	0.77	0.063	2379	684 (22%)
Comorbidity cardiovascular	1.24	0.92	1.67	0.326	0.66	0.48	0.92	0.139	1819	1244 (41%)
Comorbidity kidney	1.27	0.93	1.74	0.357	0.65	0.46	0.92	0.132	1705	1358 (44%)
Comorbidity diabetes	1.10	0.82	1.48	0.187	0.63	0.45	0.88	0.108	1824	1239 (40%)
BASFI	0.90	0.73	1.10	0.020	0.55	0.43	0.69	0.024	2869	194 (6%)
BASMI (3pt)	1.02	0.69	1.50	0.099	0.39	0.24	0.64	0.129	595	2468 (81%)
Patient global	0.91	0.74	1.11	0.007	0.52	0.41	0.65	0.007	3044	19 (1%)
Physician global	0.92	0.74	1.14	0.009	0.51	0.39	0.65	0.017	2605	458 (15%)
Pain (VAS)	0.86	0.70	1.06	0.058	0.47	0.37	0.61	0.049	2840	223 (7%)
Fatigue	0.92	0.75	1.12	0.004	0.52	0.41	0.66	0.003	2995	68 (2%)
HAQ	1.02	0.77	1.35	0.102	0.54	0.39	0.74	0.018	1746	1317 (43%)
csDMARD	0.96	0.72	1.29	0.048	0.51	0.36	0.71	0.014	1535	1528 (50%)
NSAID	1.00	0.74	1.33	0.079	0.62	0.44	0.87	0.095	1655	1408 (46%)

= Highest OR value
= Lowest OR value

Supplementary data: Association between BMI at baseline and ASDAS-MI at 12 months

Model 1 ---------------------------- Crude (unadjusted) association

Exposure	BMI
Outcome	ASDAS-MI
Timepoint	12 months
N (countries)	12
N (patients)	3160

		ASDAS-MI	
		No	Yes
BMI	Normal	778	588
	Overweight	700	455
	Obese	433	206

OR	95%CI-Lo	95%CI-Hi
0.86	0.73	1.01
0.63	0.52	0.77

Model 2 ---------------------------- Model 1, adjusted for: age, gender, country, calendar year, disease duration and disease activity

N (countries) 12
N (patients) 3063

Reference country = Switzerland (CH)
Blank line = No data for this analysis

	OR	95%CI-Lo	95%CI-Hi
BMI = Overweight	0.77	0.63	0.94
BMI = Obese	0.48	0.37	0.61
Age	0.97	0.96	0.98
Male	1.50	1.24	1.80
BASDAI	1.05	1.04	1.05
DiseaseDuration_2	0.75	0.57	0.98
DiseaseDuration_3	1.00	0.80	1.25
DiseaseDuration_4	1.07	0.83	1.38
DiseaseDuration_5	0.71	0.37	1.35
CalendarYear	0.99	0.95	1.02
Czech Republic (CZ)	4.32	3.06	6.15
Denmark (DK)	0.87	0.61	1.23
Spain (ES)			
Iceland (IS)	0.29	0.01	2.16
Italy (IT)	0.58	0.20	1.41
Netherlands (NL)	0.53	0.16	1.47
Norway (NO)			
Portugal (PT)	1.67	1.10	2.55
Romania (RO)	11.47	6.44	20.92
Finland (SF)	1.92	1.10	3.28
Slovenia (SI)	1.01	0.55	1.81
Turkey (TR)	3.40	2.21	5.24
United Kingdom (UK)	0.88	0.48	1.57

Model 3a/3b ---------------------- Model 2 additionally adjusted, in turn, for Smoking and Alcohol

Individual models. Result = OR (BMI) from each model

		OR (over-wt)	95%CI-Lo	95%CI-Hi	OR (ov)↑↓	OR (obese)	95%CI-Lo	95%CI-Hi	OR (ob)↑↓	N	N↓
(3a)	Smoking	0.77	0.63	0.94	0.005	0.47	0.37	0.60	0.007	2864	199 (6%)
(3b)	Alcohol	0.65	0.46	0.91	0.126	0.51	0.32	0.79	0.030	976	2087 (68%)

Sensitivity analysis -------------- Model 2 additionally adjusted, in turn, for other variables shown

Individual models. Result = OR (BMI) from each model

	OR (over-wt)	95%CI-Lo	95%CI-Hi	OR (ov)↑↓	OR (obese)	95%CI-Lo	95%CI-Hi	OR (ob)↑↓	N	N↓
Yrs since symptom onset	0.81	0.66	1.00	0.038	0.52	0.40	0.67	0.042	2593	470 (15%)
HLA-B27 status	0.84	0.67	1.06	0.071	0.49	0.37	0.64	0.009	2220	843 (28%)
ESR	0.77	0.58	1.01	0.003	0.45	0.32	0.61	0.030	1872	1191 (39%)
CRP	0.72	0.58	0.90	0.050	0.40	0.30	0.52	0.080	2971	92 (3%)
mNY criteria	0.81	0.59	1.11	0.037	0.57	0.38	0.85	0.098	1207	1856 (61%)
ASAS criteria	0.79	0.62	0.99	0.014	0.49	0.37	0.64	0.008	2155	908 (30%)
IBD	0.82	0.66	1.03	0.050	0.50	0.38	0.65	0.020	2392	671 (22%)
Uveitis	0.80	0.64	1.00	0.025	0.49	0.37	0.64	0.013	2387	676 (22%)
Psoriasis	0.83	0.67	1.04	0.062	0.52	0.39	0.67	0.038	2379	684 (22%)
Comorbidity cardiovascular	0.80	0.62	1.05	0.032	0.51	0.38	0.70	0.037	1819	1244 (41%)
Comorbidity kidney	0.78	0.59	1.02	0.004	0.51	0.37	0.70	0.034	1705	1358 (44%)
Comorbidity diabetes	0.74	0.57	0.96	0.031	0.50	0.37	0.68	0.023	1824	1239 (40%)
BASFI	0.75	0.62	0.92	0.019	0.48	0.37	0.60	0.001	2869	194 (6%)
BASMI (3pt)	0.94	0.61	1.44	0.166	0.39	0.21	0.70	0.091	595	2468 (81%)
Patient global	0.76	0.63	0.93	0.008	0.47	0.37	0.60	0.007	3044	19 (1%)
Physician global	0.80	0.65	0.99	0.030	0.46	0.36	0.60	0.015	2605	458 (15%)
Pain (VAS)	0.78	0.63	0.95	0.004	0.46	0.36	0.60	0.012	2840	223 (7%)
Fatigue	0.77	0.63	0.94	0.003	0.48	0.38	0.61	0.003	2995	68 (2%)
HAQ	0.75	0.58	0.97	0.020	0.44	0.32	0.60	0.035	1746	1317 (43%)
csDMARD	0.80	0.60	1.08	0.030	0.42	0.30	0.60	0.052	1535	1528 (50%)
NSAID	0.66	0.51	0.86	0.112	0.45	0.33	0.61	0.029	1655	1408 (46%)

(red) = Highest OR value
(blue) = Lowest OR value

Supplementary data: Association between BMI at baseline and ASAS-20 at 12 months

Model 1 ---------------------------- Crude (unadjusted) association

Exposure	BMI
Outcome	ASAS-20
Timepoint	12 months
N (countries)	11
N (patients)	3829

		ASAS-20	
		No	Yes
BMI	Normal	782	858
	Overweight	704	700
	Obese	433	352

OR	95%CI-Lo	95%CI-Hi
0.91	0.79	1.05
0.74	0.62	0.88

Model 2 ---------------------------- Model 1, adjusted for: age, gender, country, calendar year, disease duration and disease activity

N (countries)	11
N (patients)	3432

Note		OR	95%CI-Lo	95%CI-Hi
	BMI = Overweight	0.86	0.71	1.02
	BMI = Obese	0.59	0.48	0.74
	Age	0.98	0.97	0.99
	Male	1.36	1.15	1.60
	BASDAI	1.03	1.03	1.03
	DiseaseDuration_2	0.73	0.57	0.92
	DiseaseDuration_3	0.92	0.75	1.14
	DiseaseDuration_4	1.05	0.83	1.32
	DiseaseDuration_5	0.51	0.29	0.89
	CalendarYear	1.00	0.97	1.03
Reference country = Switzerland (CH)	Czech Republic (CZ)	2.07	1.53	2.79
Blank line = No data for this analysis	Denmark (DK)	0.57	0.43	0.74
	Spain (ES)			
	Iceland (IS)	0.56	0.12	2.59
Rounding to 2dp. Actual value = non-zero.	Italy (IT)	0.00	0.00	0.00
	Netherlands (NL)	0.13	0.05	0.31
	Norway (NO)			
Rounding to 2dp. Actual value = non-zero.	Portugal (PT)	0.00	0.00	0.44
	Romania (RO)			
	Finland (SF)	0.50	0.34	0.72
	Slovenia (SI)	0.30	0.19	0.46
	Turkey (TR)	1.83	1.28	2.63
	United Kingdom (UK)	0.98	0.64	1.52

Model 3a/3b ---------------------- Model 2 additionally adjusted, in turn, for Smoking and Alcohol

Individual models. Result = OR (BMI) from each model

		OR (over-wt)	95%CI-Lo	95%CI-Hi	OR (ov)↑↓	OR (obese)	95%CI-Lo	95%CI-Hi	OR (ob)↑↓	N	N↓
(3a)	Smoking	0.84	0.70	1.01	0.016	0.57	0.46	0.71	0.024	3134	298 (9%)
(3b)	Alcohol	0.72	0.54	0.97	0.131	0.44	0.30	0.64	0.151	1087	2345 (68%)

Sensitivity analysis -------------- Model 2 additionally adjusted, in turn, for other variables shown

Individual models. Result = OR (BMI) from each model

	OR (over-wt)	95%CI-Lo	95%CI-Hi	OR (ov)↑↓	OR (obese)	95%CI-Lo	95%CI-Hi	OR (ob)↑↓	N	N↓
Yrs since symptom onset	0.84	0.69	1.03	0.011	0.59	0.46	0.75	0.005	2644	788 (23%)
HLA-B27 status	0.87	0.70	1.10	0.020	0.60	0.46	0.78	0.005	2301	1131 (33%)
ESR	0.88	0.67	1.16	0.027	0.67	0.49	0.92	0.079	1886	1546 (45%)
CRP	0.84	0.69	1.02	0.015	0.56	0.44	0.70	0.035	3005	427 (12%)
mNY criteria	0.76	0.57	1.02	0.094	0.47	0.33	0.66	0.126	1189	2243 (65%)
ASAS criteria	0.83	0.66	1.04	0.022	0.59	0.45	0.77	0.002	2186	1246 (36%)
IBD	0.89	0.72	1.10	0.033	0.62	0.48	0.80	0.028	2540	892 (26%)
Uveitis	0.88	0.72	1.09	0.029	0.64	0.50	0.82	0.046	2525	907 (26%)
Psoriasis	0.90	0.73	1.11	0.046	0.65	0.51	0.83	0.059	2519	913 (27%)
Comorbidity cardiovascular	1.07	0.82	1.40	0.217	0.80	0.59	1.08	0.204	1834	1598 (47%)
Comorbidity kidney	1.12	0.85	1.48	0.265	0.87	0.64	1.20	0.281	1705	1727 (50%)
Comorbidity diabetes	0.98	0.76	1.28	0.129	0.80	0.59	1.09	0.206	1854	1578 (46%)
BASFI	0.86	0.72	1.03	0.005	0.60	0.48	0.74	0.004	3392	40 (1%)
BASMI (3pt)	0.79	0.53	1.16	0.069	0.28	0.17	0.46	0.310	629	2803 (82%)
Patient global	0.86	0.71	1.03	0.002	0.57	0.46	0.71	0.021	3282	150 (4%)
Physician global	0.83	0.69	1.01	0.021	0.57	0.45	0.72	0.023	2993	439 (13%)
Pain (VAS)	0.87	0.72	1.05	0.013	0.58	0.46	0.72	0.017	3276	156 (5%)
Fatigue	0.86	0.72	1.03	0.003	0.59	0.48	0.74	0.003	3248	184 (5%)
HAQ	1.05	0.83	1.35	0.200	0.76	0.57	1.02	0.168	1986	1446 (42%)
csDMARD	0.89	0.68	1.17	0.039	0.56	0.41	0.77	0.028	1699	1733 (50%)
NSAID	0.92	0.70	1.22	0.068	0.67	0.49	0.92	0.080	1672	1760 (51%)

[red] = Highest OR value
[blue] = Lowest OR value

Supplementary data: Association between BMI at baseline and ASAS-40 at 12 months

Model 1 ---------------------------- Crude (unadjusted) association

Exposure	BMI
Outcome	ASAS-40
Timepoint	12 months
N (countries)	11
N (patients)	4288

		ASAS-40	
		No	Yes
BMI	Normal	1177	646
	Overweight	1040	531
	Obese	651	243

OR	95%CI-Lo	95%CI-Hi
0.93	0.81	1.07
0.68	0.57	0.81

Model 2 ---------------------------- Model 1, adjusted for: age, gender, country, calendar year, disease duration and disease activity

N (countries) 11
N (patients) 3712

		OR	95%CI-Lo	95%CI-Hi
	BMI = Overweight	0.94	0.79	1.12
	BMI = Obese	0.61	0.49	0.75
	Age	0.97	0.97	0.98
	Male	1.27	1.08	1.50
	BASDAI	1.03	1.02	1.03
	DiseaseDuration_2	0.89	0.71	1.13
	DiseaseDuration_3	0.88	0.72	1.08
	DiseaseDuration_4	1.04	0.83	1.31
	DiseaseDuration_5	0.55	0.30	0.99
	CalendarYear	1.02	0.99	1.05
Reference country = Switzerland (CH)	Czech Republic (CZ)	2.18	1.66	2.89
Blank line = No data for this analysis	Denmark (DK)	0.62	0.48	0.82
	Spain (ES)			
	Iceland (IS)	0.61	0.12	2.53
Rounding to 2dp. Actual value = non-zero.	Italy (IT)	0.00	0.00	0.00
	Netherlands (NL)	0.15	0.04	0.40
	Norway (NO)			
Rounding to 2dp. Actual value = non-zero.	Portugal (PT)	0.00	0.00	0.01
	Romania (RO)			
	Finland (SF)	0.52	0.35	0.77
	Slovenia (SI)	0.17	0.10	0.27
	Turkey (TR)	1.93	1.36	2.75
	United Kingdom (UK)	1.01	0.65	1.54

Model 3a/3b ---------------------- Model 2 additionally adjusted, in turn, for Smoking and Alcohol

Individual models. Result = OR (BMI) from each model

		OR (over-wt)	95%CI-Lo	95%CI-Hi	OR (ov)↑↓	OR (obese)	95%CI-Lo	95%CI-Hi	OR (ob)↑↓	N	N↓
(3a)	Smoking	0.95	0.80	1.14	0.012	0.59	0.47	0.73	0.018	3327	385 (10%)
(3b)	Alcohol	0.85	0.63	1.15	0.091	0.50	0.33	0.74	0.108	1127	2585 (70%)

Sensitivity analysis -------------- Model 2 additionally adjusted, in turn, for other variables shown

Individual models. Result = OR (BMI) from each model

	OR (over-wt)	95%CI-Lo	95%CI-Hi	OR (ov)↑↓	OR (obese)	95%CI-Lo	95%CI-Hi	OR (ob)↑↓	N	N↓
Yrs since symptom onset	0.97	0.80	1.18	0.034	0.61	0.48	0.77	0.004	2734	978 (26%)
HLA-B27 status	1.06	0.85	1.31	0.116	0.65	0.51	0.84	0.047	2457	1255 (34%)
ESR	1.08	0.84	1.40	0.140	0.69	0.51	0.99	0.079	2136	1576 (42%)
CRP	0.95	0.79	1.14	0.007	0.58	0.47	0.73	0.022	3175	537 (14%)
mNY criteria	1.07	0.80	1.44	0.130	0.56	0.38	0.81	0.047	1307	2405 (65%)
ASAS criteria	1.06	0.85	1.31	0.115	0.65	0.50	0.83	0.041	2317	1395 (38%)
IBD	1.04	0.85	1.27	0.096	0.68	0.53	0.87	0.074	2729	983 (26%)
Uveitis	1.01	0.83	1.24	0.071	0.67	0.53	0.86	0.068	2704	1008 (27%)
Psoriasis	1.07	0.87	1.31	0.127	0.70	0.55	0.89	0.095	2697	1015 (27%)
Comorbidity cardiovascular	1.24	0.96	1.59	0.297	0.81	0.61	1.08	0.204	2010	1702 (46%)
Comorbidity kidney	1.23	0.95	1.59	0.290	0.84	0.62	1.13	0.231	1874	1838 (50%)
Comorbidity diabetes	1.11	0.87	1.42	0.169	0.79	0.59	1.05	0.183	2023	1689 (46%)
BASFI	0.94	0.79	1.12	0.002	0.61	0.49	0.76	0.003	3653	59 (2%)
BASMI (3pt)	0.83	0.56	1.24	0.108	0.38	0.22	0.65	0.225	649	3063 (83%)
Patient global	0.94	0.78	1.13	0.001	0.58	0.46	0.72	0.027	3435	277 (7%)
Physician global	0.93	0.77	1.12	0.013	0.55	0.44	0.69	0.055	3137	575 (15%)
Pain (VAS)	0.95	0.79	1.14	0.010	0.58	0.47	0.73	0.022	3453	259 (7%)
Fatigue	0.94	0.79	1.12	0.001	0.61	0.49	0.75	0.002	3433	279 (8%)
HAQ	1.15	0.92	1.45	0.214	0.72	0.55	0.95	0.112	2136	1576 (42%)
csDMARD	0.96	0.73	1.24	0.015	0.64	0.47	0.87	0.035	1892	1820 (49%)
NSAID	1.03	0.80	1.33	0.091	0.68	0.51	0.91	0.073	1740	1972 (53%)

[red] = Highest OR value
[blue] = Lowest OR value

Supplementary data: Association between BMI at baseline and ASAS-5of6 at 12 months

Model 1 ---------------------------- Crude (unadjusted) association

Exposure	BMI
Outcome	ASAS-5of6
Timepoint	12 months
N (countries)	12
N (patients)	3208

		ASAS-5of6	
		No	Yes
BMI	Normal	775	563
	Overweight	727	472
	Obese	452	219

OR	95%CI-Lo	95%CI-Hi
0.89	0.76	1.05
0.67	0.55	0.81

Model 2 ---------------------------- Model 1, adjusted for: age, gender, country, calendar year, disease duration and disease activity

N (countries)	11
N (patients)	2819

Reference country = Switzerland (CH)
Blank line = No data for this analysis

	OR	95%CI-Lo	95%CI-Hi
BMI = Overweight	0.88	0.72	1.08
BMI = Obese	0.56	0.44	0.72
Age	0.97	0.96	0.98
Male	1.48	1.23	1.79
BASDAI	1.02	1.01	1.02
DiseaseDuration_2	0.71	0.54	0.94
DiseaseDuration_3	0.97	0.77	1.23
DiseaseDuration_4	1.00	0.77	1.29
DiseaseDuration_5	0.49	0.24	0.96
CalendarYear	1.01	0.98	1.05
Czech Republic (CZ)	6.16	4.45	8.57
Denmark (DK)	0.91	0.67	1.23
Spain (ES)			
Iceland (IS)	1.17	0.14	7.57
Italy (IT)			
Netherlands (NL)	0.27	0.09	0.68
Norway (NO)			
Portugal (PT)	0.07	0.02	0.20
Romania (RO)	1.93	0.92	4.09
Finland (SF)	0.95	0.61	1.47
Slovenia (SI)	0.51	0.34	0.76
Turkey (TR)	3.32	2.21	5.01
United Kingdom (UK)	0.94	0.55	1.60

Model 3a/3b ---------------------- Model 2 additionally adjusted, in turn, for Smoking and Alcohol

Individual models. Result = OR (BMI) from each model

		OR (over-wt)	95%CI-Lo	95%CI-Hi	OR (ov) ↑↓	OR (obese)	95%CI-Lo	95%CI-Hi	OR (ob) ↑↓	N	N↓
(3a)	Smoking	0.88	0.72	1.09	0.000	0.55	0.42	0.70	0.014	2575	244 (9%)
(3b)	Alcohol	0.68	0.48	0.98	0.198	0.47	0.29	0.74	0.090	795	2024 (72%)

Sensitivity analysis -------------- Model 2 additionally adjusted, in turn, for other variables shown

Individual models. Result = OR (BMI) from each model

	OR (over-wt)	95%CI-Lo	95%CI-Hi	OR (ov) ↑↓	OR (obese)	95%CI-Lo	95%CI-Hi	OR (ob) ↑↓	N	N↓
Yrs since symptom onset	0.84	0.67	1.05	0.045	0.53	0.40	0.70	0.030	2114	705 (25%)
HLA-B27 status	0.94	0.73	1.20	0.057	0.57	0.43	0.77	0.013	1971	848 (30%)
ESR	0.99	0.74	1.33	0.107	0.53	0.38	0.75	0.025	1751	1068 (38%)
CRP	0.85	0.68	1.06	0.031	0.50	0.38	0.65	0.063	2591	228 (8%)
mNY criteria	0.92	0.67	1.26	0.037	0.56	0.38	0.83	0.004	1089	1730 (61%)
ASAS criteria	0.93	0.72	1.19	0.044	0.57	0.42	0.76	0.005	1910	909 (32%)
IBD	0.93	0.74	1.17	0.047	0.58	0.43	0.76	0.015	2189	630 (22%)
Uveitis	0.93	0.74	1.17	0.046	0.58	0.44	0.77	0.023	2176	643 (23%)
Psoriasis	0.98	0.78	1.24	0.101	0.62	0.47	0.82	0.058	2175	644 (23%)
Comorbidity cardiovascular	1.17	0.88	1.55	0.286	0.72	0.51	1.00	0.157	1683	1136 (40%)
Comorbidity kidney	1.24	0.92	1.66	0.356	0.75	0.53	1.05	0.188	1571	1248 (44%)
Comorbidity diabetes	1.06	0.80	1.41	0.178	0.67	0.48	0.94	0.114	1693	1126 (40%)
BASFI	0.89	0.72	1.09	0.006	0.57	0.44	0.73	0.010	2750	69 (2%)
BASMI (3pt)	0.77	0.49	1.21	0.113	0.37	0.20	0.65	0.195	484	2335 (83%)
Patient global	0.88	0.71	1.08	0.006	0.54	0.41	0.70	0.022	2624	195 (7%)
Physician global	0.86	0.69	1.08	0.018	0.53	0.41	0.70	0.028	2391	428 (15%)
Pain (VAS)	0.89	0.73	1.10	0.012	0.54	0.42	0.70	0.021	2675	144 (5%)
Fatigue	0.89	0.72	1.09	0.004	0.56	0.44	0.72	0.004	2687	132 (5%)
HAQ	1.14	0.87	1.50	0.260	0.73	0.53	1.00	0.166	1660	1159 (41%)
csDMARD	0.93	0.69	1.24	0.044	0.49	0.35	0.70	0.065	1417	1402 (50%)
NSAID	0.88	0.65	1.18	0.007	0.55	0.39	0.79	0.006	1404	1415 (50%)

= Highest OR value
= Lowest OR value

Supplementary data: Association between ALCOHOL at baseline and BASDAI-50 at 3 months

Model 1 ---------------------------- Crude (unadjusted) association

Exposure	Alcohol
Outcome	BASDAI-50
Timepoint	3 months
N (countries)	7
N (patients)	1926

		BASDAI-50	
		No	Yes
Alcohol	No	708	342
	Yes	560	316

OR	95%CI-Lo	95%CI-Hi
1.17	0.97	1.41

Model 2 ---------------------------- Model 1, adjusted for: age, gender, country, calendar year, disease duration and disease activity

N (countries)	7
N (patients)	1926

Reference country = Switzerland (CH)
Blank line = No data for this analysis

	OR	95%CI-Lo	95%CI-Hi
Alcohol = Yes	1.47	1.18	1.85
Age	0.97	0.96	0.98
Male	1.51	1.23	1.85
BASDAI	1.00	1.00	1.01
DiseaseDuration_2	0.79	0.59	1.06
DiseaseDuration_3	1.09	0.84	1.41
DiseaseDuration_4	0.99	0.74	1.33
DiseaseDuration_5	1.41	0.82	2.40
CalendarYear	1.02	0.98	1.06
Czech Republic (CZ)			
Denmark (DK)	2.34	0.34	46.36
Spain (ES)			
Iceland (IS)	4.78	0.33	144.00
Italy (IT)	3.14	0.40	65.30
Netherlands (NL)			
Norway (NO)			
Portugal (PT)	3.82	0.55	75.64
Romania (RO)			
Finland (SF)			
Slovenia (SI)			
Turkey (TR)	6.58	0.54	175.78
United Kingdom (UK)	1.93	0.27	38.53

Model 3a/3b ---------------------- Model 2 additionally adjusted, in turn, for Smoking and BMI

Individual models. Result = OR (Alcohol) from each model

		OR (alcohol)	95%CI-Lo	95%CI-Hi	OR↑↓	N	N↓
(3a)	Smoking	1.47	1.17	1.84	0.006	1920	6 (0%)
(3b)	BMI	1.56	1.22	2.00	0.082	1527	399 (21%)

Sensitivity analysis -------------- Model 2 additionally adjusted, in turn, for other variables shown

Individual models. Result = OR (Alcohol) from each model

	OR (alcohol)	95%CI-Lo	95%CI-Hi	OR↑↓	N	N↓
Yrs since symptom onset	1.69	1.32	2.16	0.212	1662	264 (14%)
HLA-B27 status	1.57	1.17	2.11	0.095	1223	703 (37%)
ESR	0.96	0.58	1.59	0.518	660	1266 (66%)
CRP	1.57	1.23	2.02	0.098	1668	258 (13%)
mNY criteria	1.43	1.05	1.94	0.045	1115	811 (42%)
ASAS criteria	1.64	1.24	2.18	0.168	1319	607 (32%)
IBD	1.67	1.26	2.22	0.194	1319	607 (32%)
Uveitis	1.62	1.23	2.16	0.149	1317	609 (32%)
Psoriasis	1.70	1.29	2.25	0.225	1346	580 (30%)
Comorbidity cardiovascular	1.52	0.99	2.35	0.041	725	1201 (62%)
Comorbidity kidney	1.71	1.09	2.71	0.231	687	1239 (64%)
Comorbidity diabetes	1.72	1.11	2.67	0.240	724	1202 (62%)
BASFI	1.42	1.13	1.79	0.058	1858	68 (4%)
BASMI (3pt)	1.19	0.83	1.72	0.281	772	1154 (60%)
Patient global	1.48	1.18	1.86	0.001	1896	30 (2%)
Physician global	1.45	1.12	1.88	0.027	1439	487 (25%)
Pain (VAS)	1.67	1.31	2.15	0.200	1370	556 (29%)
Fatigue	1.53	1.21	1.92	0.050	1885	41 (2%)
HAQ	1.26	0.86	1.84	0.219	550	1376 (71%)
csDMARD	1.55	1.06	2.30	0.080	743	1183 (61%)
NSAID	1.51	1.05	2.16	0.031	984	942 (49%)

= Highest OR value (red)
= Lowest OR value (blue)

Supplementary data: Association between ALCOHOL at baseline and ASDAS-Inactive at 3 months

Model 1 ---------------------------- Crude (unadjusted) association

Exposure	Alcohol
Outcome	ASDAS-Inactive
Timepoint	3 months
N (countries)	7
N (patients)	2179

		ASDAS-Inactive	
		No	Yes
Alcohol	No	963	194
	Yes	805	217

OR	95%CI-Lo	95%CI-Hi
1.34	1.08	1.66

Model 2 ---------------------------- Model 1, adjusted for: age, gender, country, calendar year, disease duration and disease activity

N (countries)	7
N (patients)	1713

Reference country = Switzerland (CH)
Blank line = No data for this analysis

	OR	95%CI-Lo	95%CI-Hi
Alcohol = Yes	1.46	1.08	1.97
Age	0.96	0.95	0.97
Male	1.75	1.33	2.31
BASDAI	0.97	0.97	0.98
DiseaseDuration_2	0.82	0.55	1.20
DiseaseDuration_3	1.15	0.82	1.62
DiseaseDuration_4	0.98	0.66	1.46
DiseaseDuration_5	1.23	0.60	2.42
CalendarYear	1.02	0.97	1.08
Czech Republic (CZ)			
Denmark (DK)	0.92	0.13	18.55
Spain (ES)			
Iceland (IS)	0.91	0.03	31.12
Italy (IT)	0.56	0.05	13.07
Netherlands (NL)			
Norway (NO)			
Portugal (PT)	1.48	0.21	29.81
Romania (RO)			
Finland (SF)			
Slovenia (SI)			
Turkey (TR)	0.64	0.04	18.39
United Kingdom (UK)	0.62	0.08	12.61

Model 3a/3b ---------------------- Model 2 additionally adjusted, in turn, for Smoking and BMI

Individual models. Result = OR (Alcohol) from each model

		OR (alcohol)	95%CI-Lo	95%CI-Hi	OR↑↓	N	N↓
(3a)	Smoking	1.45	1.07	1.96	0.009	1707	6 (0%)
(3b)	BMI	1.44	1.04	2.01	0.011	1348	365 (21%)

Sensitivity analysis -------------- Model 2 additionally adjusted, in turn, for other variables shown

Individual models. Result = OR (Alcohol) from each model

	OR (alcohol)	95%CI-Lo	95%CI-Hi	OR↑↓	N	N↓
Yrs since symptom onset	1.61	1.17	2.22	0.153	1493	220 (13%)
HLA-B27 status	1.69	1.15	2.50	0.232	1094	619 (36%)
ESR	0.99	0.51	1.88	0.466	601	1112 (65%)
CRP	1.35	0.98	1.87	0.101	1528	185 (11%)
mNY criteria	1.54	1.01	2.35	0.082	1001	712 (42%)
ASAS criteria	1.69	1.16	2.47	0.237	1189	524 (31%)
IBD	1.67	1.15	2.46	0.218	1187	526 (31%)
Uveitis	1.65	1.14	2.42	0.198	1189	524 (31%)
Psoriasis	1.70	1.17	2.49	0.247	1208	505 (29%)
Comorbidity cardiovascular	1.66	0.93	3.01	0.209	660	1053 (61%)
Comorbidity kidney	1.62	0.89	2.97	0.165	620	1093 (64%)
Comorbidity diabetes	1.55	0.86	2.81	0.091	659	1054 (62%)
BASFI	1.37	1.01	1.87	0.082	1662	51 (3%)
BASMI (3pt)	1.03	0.65	1.66	0.422	708	1005 (59%)
Patient global	1.40	1.04	1.89	0.057	1692	21 (1%)
Physician global	1.34	0.96	1.89	0.111	1292	421 (25%)
Pain (VAS)	1.61	1.15	2.27	0.156	1207	506 (30%)
Fatigue	1.44	1.07	1.96	0.012	1687	26 (2%)
HAQ	1.08	0.66	1.77	0.380	485	1228 (72%)
csDMARD	2.45	1.38	4.44	0.994	662	1051 (61%)
NSAID	1.52	0.95	2.43	0.063	878	835 (49%)

= Highest OR value
= Lowest OR value

Supplementary data: Association between ALCOHOL at baseline and ASDAS-LDA at 3 months

Model 1 ---------------------------- Crude (unadjusted) association

Exposure	Alcohol
Outcome	ASDAS-LDA
Timepoint	3 months
N (countries)	7
N (patients)	2179

		ASDAS-LDA	
		No	Yes
Alcohol	No	704	453
	Yes	535	487

OR	95%CI-Lo	95%CI-Hi
1.41	1.19	1.68

Model 2 ---------------------------- Model 1, adjusted for: age, gender, country, calendar year, disease duration and disease activity

N (countries)	7
N (patients)	1713

Reference country = Switzerland (CH)
Blank line = No data for this analysis

	OR	95%CI-Lo	95%CI-Hi
Alcohol = Yes	1.56	1.23	2.00
Age	0.97	0.96	0.98
Male	1.48	1.20	1.84
BASDAI	0.97	0.96	0.97
DiseaseDuration_2	0.84	0.62	1.16
DiseaseDuration_3	1.03	0.77	1.37
DiseaseDuration_4	1.05	0.77	1.43
DiseaseDuration_5	0.94	0.51	1.71
CalendarYear	1.02	0.98	1.06
Czech Republic (CZ)			
Denmark (DK)	1.08	0.16	8.81
Spain (ES)			
Iceland (IS)	0.91	0.06	16.16
Italy (IT)	2.08	0.26	19.86
Netherlands (NL)			
Norway (NO)			
Portugal (PT)	1.75	0.26	14.39
Romania (RO)			
Finland (SF)			
Slovenia (SI)			
Turkey (TR)	0.77	0.06	10.89
United Kingdom (UK)	0.98	0.14	8.16

Model 3a/3b ---------------------- Model 2 additionally adjusted, in turn, for Smoking and BMI

Individual models. Result = OR (Alcohol) from each model

		OR (alcohol)	95%CI-Lo	95%CI-Hi	OR↑↓	N	N↓
(3a)	Smoking	1.54	1.21	1.97	0.023	1707	6 (0%)
(3b)	BMI	1.62	1.24	2.13	0.058	1348	365 (21%)

Sensitivity analysis -------------- Model 2 additionally adjusted, in turn, for other variables shown

Individual models. Result = OR (Alcohol) from each model

	OR (alcohol)	95%CI-Lo	95%CI-Hi	OR↑↓	N	N↓
Yrs since symptom onset	1.79	1.37	2.33	0.221	1493	220 (13%)
HLA-B27 status	1.83	1.33	2.52	0.262	1094	619 (36%)
ESR	1.35	0.79	2.33	0.216	601	1112 (65%)
CRP	1.70	1.31	2.21	0.139	1528	185 (11%)
mNY criteria	1.68	1.21	2.34	0.113	1001	712 (42%)
ASAS criteria	1.84	1.36	2.50	0.279	1189	524 (31%)
IBD	1.85	1.37	2.53	0.290	1187	526 (31%)
Uveitis	1.87	1.38	2.56	0.310	1189	524 (31%)
Psoriasis	1.88	1.39	2.56	0.318	1208	505 (29%)
Comorbidity cardiovascular	1.89	1.18	3.08	0.329	660	1053 (61%)
Comorbidity kidney	2.04	1.24	3.40	0.471	620	1093 (64%)
Comorbidity diabetes	1.97	1.22	3.21	0.402	659	1054 (62%)
BASFI	1.52	1.18	1.95	0.047	1662	51 (3%)
BASMI (3pt)	1.46	1.00	2.13	0.104	708	1005 (59%)
Patient global	1.54	1.21	1.97	0.021	1692	21 (1%)
Physician global	1.45	1.10	1.91	0.114	1292	421 (25%)
Pain (VAS)	1.63	1.25	2.13	0.065	1207	506 (30%)
Fatigue	1.57	1.23	2.02	0.008	1687	26 (2%)
HAQ	1.07	0.70	1.63	0.500	485	1228 (72%)
csDMARD	1.60	1.05	2.45	0.032	662	1051 (61%)
NSAID	1.72	1.16	2.56	0.152	878	835 (49%)

(red) = Highest OR value
(blue) = Lowest OR value

Supplementary data: Association between ALCOHOL at baseline and ASDAS-CII at 3 months

Model 1 ---------------------------- Crude (unadjusted) association

Exposure	Alcohol
Outcome	ASDAS-CII
Timepoint	3 months
N (countries)	7
N (patients)	1556

		ASDAS-CII No	ASDAS-CII Yes
Alcohol	No	495	387
	Yes	376	298

OR	95%CI-Lo	95%CI-Hi
1.01	0.83	1.24

Model 2 ---------------------------- Model 1, adjusted for: age, gender, country, calendar year, disease duration and disease activity

N (countries)	7
N (patients)	1555

Reference country = Switzerland (CH)
Blank line = No data for this analysis

	OR	95%CI-Lo	95%CI-Hi
Alcohol = Yes	1.34	1.05	1.71
Age	0.99	0.98	1.00
Male	1.54	1.24	1.91
BASDAI	1.02	1.01	1.02
DiseaseDuration_2	0.63	0.46	0.86
DiseaseDuration_3	0.72	0.54	0.95
DiseaseDuration_4	0.78	0.57	1.05
DiseaseDuration_5	0.74	0.39	1.38
CalendarYear	1.02	0.98	1.06
Czech Republic (CZ)			
Denmark (DK)	0.54	0.07	3.41
Spain (ES)			
Iceland (IS)	0.43	0.01	16.60
Italy (IT)	0.34	0.04	2.61
Netherlands (NL)			
Norway (NO)			
Portugal (PT)	0.92	0.12	5.78
Romania (RO)			
Finland (SF)			
Slovenia (SI)			
Turkey (TR)	0.47	0.03	5.27
United Kingdom (UK)	0.41	0.05	2.65

Model 3a/3b ---------------------- Model 2 additionally adjusted, in turn, for Smoking and BMI

Individual models. Result = OR (Alcohol) from each model

		OR (alcohol)	95%CI-Lo	95%CI-Hi	OR↑↓	N	N↓
(3a)	Smoking	1.33	1.05	1.70	0.005	1549	6 (0%)
(3b)	BMI	1.29	0.99	1.69	0.044	1216	339 (22%)

Sensitivity analysis -------------- Model 2 additionally adjusted, in turn, for other variables shown

Individual models. Result = OR (Alcohol) from each model

	OR (alcohol)	95%CI-Lo	95%CI-Hi	OR↑↓	N	N↓
Yrs since symptom onset	1.40	1.08	1.81	0.058	1362	193 (12%)
HLA-B27 status	1.23	0.90	1.68	0.112	1003	552 (35%)
ESR	0.95	0.54	1.68	0.386	593	962 (62%)
CRP	1.52	1.16	1.99	0.178	1511	44 (3%)
mNY criteria	1.19	0.86	1.65	0.149	918	637 (41%)
ASAS criteria	1.36	1.01	1.84	0.023	1088	467 (30%)
IBD	1.37	1.01	1.85	0.030	1086	469 (30%)
Uveitis	1.28	0.95	1.73	0.061	1092	463 (30%)
Psoriasis	1.38	1.03	1.86	0.041	1108	447 (29%)
Comorbidity cardiovascular	1.24	0.79	1.96	0.097	621	934 (60%)
Comorbidity kidney	1.30	0.81	2.10	0.039	583	972 (63%)
Comorbidity diabetes	1.30	0.83	2.07	0.034	617	938 (60%)
BASFI	1.37	1.07	1.76	0.034	1513	42 (3%)
BASMI (3pt)	1.30	0.90	1.88	0.042	676	879 (57%)
Patient global	1.35	1.05	1.73	0.008	1552	3 (0%)
Physician global	1.43	1.08	1.90	0.090	1179	376 (24%)
Pain (VAS)	1.60	1.23	2.10	0.265	1079	476 (31%)
Fatigue	1.33	1.04	1.70	0.009	1531	24 (2%)
HAQ	1.39	0.91	2.14	0.056	441	1114 (72%)
csDMARD	1.44	0.96	2.18	0.102	613	942 (61%)
NSAID	1.38	0.94	2.04	0.045	814	741 (48%)

(red) = Highest OR value
(blue) = Lowest OR value

Supplementary data: Association between ALCOHOL at baseline and ASDAS-MI at 3 months

Model 1 ---------------------------- Crude (unadjusted) association

Exposure	Alcohol
Outcome	ASDAS-MI
Timepoint	3 months
N (countries)	7
N (patients)	1556

		ASDAS-MI No	ASDAS-MI Yes
Alcohol	No	661	221
	Yes	516	158

OR	95%CI-Lo	95%CI-Hi
0.92	0.72	1.16

Model 2 ---------------------------- Model 1, adjusted for: age, gender, country, calendar year, disease duration and disease activity

N (countries)	6
N (patients)	1550

Reference country = Switzerland (CH)
Blank line = No data for this analysis

	OR	95%CI-Lo	95%CI-Hi
Alcohol = Yes	1.38	1.04	1.85
Age	0.97	0.96	0.98
Male	1.68	1.30	2.17
BASDAI	1.02	1.02	1.03
DiseaseDuration_2	0.61	0.42	0.89
DiseaseDuration_3	1.03	0.75	1.42
DiseaseDuration_4	0.94	0.65	1.35
DiseaseDuration_5	0.89	0.42	1.79
CalendarYear	1.01	0.96	1.06
Czech Republic (CZ)			
Denmark (DK)			
Spain (ES)			
Iceland (IS)	1.64	0.06	44.20
Italy (IT)	1.22	0.38	3.29
Netherlands (NL)			
Norway (NO)			
Portugal (PT)	2.10	1.56	2.85
Romania (RO)			
Finland (SF)			
Slovenia (SI)			
Turkey (TR)	1.11	0.06	7.03
United Kingdom (UK)	0.72	0.47	1.08

Model 3a/3b ---------------------- Model 2 additionally adjusted, in turn, for Smoking and BMI

Individual models. Result = OR (Alcohol) from each model

		OR (alcohol)	95%CI-Lo	95%CI-Hi	OR↑↓	N	N↓
(3a)	Smoking	1.36	1.02	1.82	0.020	1544	6 (0%)
(3b)	BMI	1.49	1.08	2.08	0.111	1211	339 (22%)

Sensitivity analysis -------------- Model 2 additionally adjusted, in turn, for other variables shown

Individual models. Result = OR (Alcohol) from each model

	OR (alcohol)	95%CI-Lo	95%CI-Hi	OR↑↓	N	N↓
Yrs since symptom onset	1.44	1.06	1.95	0.053	1357	193 (12%)
HLA-B27 status	1.39	0.96	2.02	0.005	998	552 (36%)
ESR	1.19	0.62	2.28	0.191	588	962 (62%)
CRP	1.59	1.15	2.20	0.203	1506	44 (3%)
mNY criteria	1.25	0.84	1.85	0.135	913	637 (41%)
ASAS criteria	1.40	0.99	2.00	0.020	1084	466 (30%)
IBD	1.36	0.95	1.95	0.023	1082	468 (30%)
Uveitis	1.33	0.93	1.91	0.055	1088	462 (30%)
Psoriasis	1.44	1.01	2.05	0.053	1105	445 (29%)
Comorbidity cardiovascular	1.33	0.79	2.26	0.057	618	932 (60%)
Comorbidity kidney	1.55	0.90	2.71	0.165	580	970 (63%)
Comorbidity diabetes	1.55	0.91	2.66	0.166	612	938 (61%)
BASFI	1.37	1.02	1.84	0.014	1508	42 (3%)
BASMI (3pt)	1.29	0.84	2.00	0.092	676	874 (56%)
Patient global	1.38	1.03	1.85	0.006	1547	3 (0%)
Physician global	1.45	1.03	2.03	0.064	1174	376 (24%)
Pain (VAS)	1.55	1.12	2.15	0.166	1074	476 (31%)
Fatigue	1.32	0.98	1.77	0.067	1526	24 (2%)
HAQ	1.58	0.95	2.65	0.193	441	1109 (72%)
csDMARD	1.66	1.02	2.72	0.275	608	942 (61%)
NSAID	1.49	0.95	2.34	0.105	811	739 (48%)

= Highest OR value
= Lowest OR value

Supplementary data: Association between ALCOHOL at baseline and ASAS-20 at 3 months

Model 1 ---------------------------- Crude (unadjusted) association

Exposure	Alcohol
Outcome	ASAS-20
Timepoint	3 months
N (countries)	7
N (patients)	1718

		ASAS-20	
		No	Yes
Alcohol	No	611	213
	Yes	509	385

OR	95%CI-Lo	95%CI-Hi
2.17	1.77	2.66

Model 2 ---------------------------- Model 1, adjusted for: age, gender, country, calendar year, disease duration and disease activity

N (countries)	5
N (patients)	1542

Reference country = Switzerland (CH)
Blank line = No data for this analysis

	OR	95%CI-Lo	95%CI-Hi
Alcohol = Yes	1.92	1.52	2.44
Age	0.98	0.97	0.99
Male	0.97	0.77	1.22
BASDAI	1.01	1.01	1.02
DiseaseDuration_2	0.67	0.48	0.93
DiseaseDuration_3	0.94	0.70	1.27
DiseaseDuration_4	0.85	0.61	1.19
DiseaseDuration_5	0.60	0.27	1.29
CalendarYear	1.03	0.98	1.09
Czech Republic (CZ)			
Denmark (DK)	0.54	0.07	3.39
Spain (ES)			
Iceland (IS)	0.47	0.03	7.35
Italy (IT)			
Netherlands (NL)			
Norway (NO)			
Portugal (PT)			
Romania (RO)			
Finland (SF)			
Slovenia (SI)			
Turkey (TR)	0.77	0.06	8.26
United Kingdom (UK)	0.49	0.06	3.11

Model 3a/3b ---------------------- Model 2 additionally adjusted, in turn, for Smoking and BMI

Individual models. Result = OR (Alcohol) from each model

		OR (alcohol)	95%CI-Lo	95%CI-Hi	OR↑↓	N	N↓
(3a)	Smoking	1.90	1.50	2.42	0.015	1539	3 (0%)
(3b)	BMI	1.96	1.53	2.52	0.041	1332	210 (14%)

Sensitivity analysis -------------- Model 2 additionally adjusted, in turn, for other variables shown

Individual models. Result = OR (Alcohol) from each model

	OR (alcohol)	95%CI-Lo	95%CI-Hi	OR↑↓	N	N↓
Yrs since symptom onset	2.04	1.58	2.64	0.121	1326	216 (14%)
HLA-B27 status	1.82	1.32	2.51	0.103	896	646 (42%)
ESR	1.12	1.00	1.25	0.800	322	1220 (79%)
CRP	1.96	1.51	2.55	0.044	1335	207 (13%)
mNY criteria	1.86	1.32	2.64	0.059	799	743 (48%)
ASAS criteria	1.83	1.36	2.47	0.088	1009	533 (35%)
IBD	1.94	1.44	2.63	0.021	993	549 (36%)
Uveitis	1.79	1.33	2.43	0.127	993	549 (36%)
Psoriasis	1.90	1.41	2.57	0.017	1023	519 (34%)
Comorbidity cardiovascular	2.06	1.22	3.52	0.136	454	1088 (71%)
Comorbidity kidney	2.62	1.00	6.88	0.704	411	1131 (73%)
Comorbidity diabetes	2.36	1.00	5.57	0.441	452	1090 (71%)
BASFI	1.88	1.48	2.39	0.037	1518	24 (2%)
BASMI (3pt)	1.93	1.33	2.81	0.009	623	919 (60%)
Patient global	1.88	1.48	2.39	0.043	1536	6 (0%)
Physician global	1.80	1.38	2.36	0.115	1209	333 (22%)
Pain (VAS)	1.91	1.51	2.44	0.006	1337	205 (13%)
Fatigue	1.90	1.50	2.42	0.015	1527	15 (1%)
HAQ	1.77	1.20	2.63	0.150	523	1019 (66%)
csDMARD	2.40	1.58	3.71	0.485	558	984 (64%)
NSAID	2.44	1.59	3.78	0.521	642	900 (58%)

= Highest OR value (red)
= Lowest OR value (blue)

Supplementary data: Association between ALCOHOL at baseline and ASAS-40 at 3 months

Model 1 ---------------------------- Crude (unadjusted) association

Exposure	Alcohol
Outcome	ASAS-40
Timepoint	3 months
N (countries)	7
N (patients)	1935

		ASAS-40	
		No	Yes
Alcohol	No	811	136
	Yes	746	242

OR	95%CI-Lo	95%CI-Hi
1.93	1.54	2.45

Model 2 ---------------------------- Model 1, adjusted for: age, gender, country, calendar year, disease duration and disease activity

N (countries)	5
N (patients)	1670

Reference country = Switzerland (CH)
Blank line = No data for this analysis

	OR	95%CI-Lo	95%CI-Hi
Alcohol = Yes	1.68	1.30	2.19
Age	0.97	0.96	0.98
Male	1.16	0.90	1.50
BASDAI	1.01	1.01	1.02
DiseaseDuration_2	0.78	0.55	1.12
DiseaseDuration_3	0.97	0.70	1.33
DiseaseDuration_4	0.82	0.56	1.20
DiseaseDuration_5	0.48	0.17	1.18
CalendarYear	1.05	0.99	1.11
Czech Republic (CZ)			
Denmark (DK)	1.65	0.23	33.20
Spain (ES)			
Iceland (IS)	3.33	0.18	108.94
Italy (IT)			
Netherlands (NL)			
Norway (NO)			
Portugal (PT)			
Romania (RO)			
Finland (SF)			
Slovenia (SI)			
Turkey (TR)	1.02	0.03	32.44
United Kingdom (UK)	1.64	0.22	33.39

Model 3a/3b ---------------------- Model 2 additionally adjusted, in turn, for Smoking and BMI

Individual models. Result = OR (Alcohol) from each model

		OR (alcohol)	95%CI-Lo	95%CI-Hi	OR↑↓	N	N↓
(3a)	Smoking	1.67	1.28	2.17	0.017	1666	4 (0%)
(3b)	BMI	1.72	1.30	2.28	0.034	1404	266 (16%)

Sensitivity analysis -------------- Model 2 additionally adjusted, in turn, for other variables shown

Individual models. Result = OR (Alcohol) from each model

	OR (alcohol)	95%CI-Lo	95%CI-Hi	OR↑↓	N	N↓
Yrs since symptom onset	1.84	1.39	2.45	0.159	1440	230 (14%)
HLA-B27 status	1.85	1.30	2.66	0.167	1004	666 (40%)
ESR	1.31	0.49	3.70	0.374	429	1241 (74%)
CRP	1.74	1.31	2.32	0.057	1450	220 (13%)
mNY criteria	1.89	1.29	2.80	0.208	904	766 (46%)
ASAS criteria	1.83	1.31	2.55	0.142	1111	559 (33%)
IBD	1.84	1.32	2.56	0.152	1099	571 (34%)
Uveitis	1.77	1.27	2.48	0.087	1100	570 (34%)
Psoriasis	1.85	1.33	2.59	0.162	1129	541 (32%)
Comorbidity cardiovascular	1.93	1.08	3.54	0.247	542	1128 (68%)
Comorbidity kidney	2.60	1.37	5.20	0.922	502	1168 (70%)
Comorbidity diabetes	1.96	1.00	3.83	0.273	540	1130 (68%)
BASFI	1.64	1.26	2.14	0.043	1635	35 (2%)
BASMI (3pt)	1.50	1.00	2.26	0.186	676	994 (60%)
Patient global	1.64	1.26	2.15	0.041	1663	7 (0%)
Physician global	1.58	1.18	2.12	0.106	1289	381 (23%)
Pain (VAS)	1.67	1.28	2.18	0.017	1357	313 (19%)
Fatigue	1.67	1.29	2.18	0.010	1648	22 (1%)
HAQ	1.31	0.86	2.01	0.374	537	1133 (68%)
csDMARD	1.73	1.08	2.81	0.049	621	1049 (63%)
NSAID	2.65	1.63	4.40	0.962	749	921 (55%)

= Highest OR value
= Lowest OR value

Supplementary data: Association between ALCOHOL at baseline and ASAS-5of6 at 3 months

Model 1 ---------------------------- Crude (unadjusted) association

Exposure	Alcohol
Outcome	ASAS-5of6
Timepoint	3 months
N (countries)	7
N (patients)	1415

		ASAS-5of6 No	ASAS-5of6 Yes
Alcohol	No	630	121
	Yes	495	169

OR	95%CI-Lo	95%CI-Hi
1.78	1.37	2.31

Model 2 ---------------------------- Model 1, adjusted for: age, gender, country, calendar year, disease duration and disease activity

N (countries)	6
N (patients)	1237

Reference country = Switzerland (CH)
Blank line = No data for this analysis

	OR	95%CI-Lo	95%CI-Hi
Alcohol = Yes	1.57	1.17	2.12
Age	0.97	0.96	0.98
Male	1.17	0.87	1.56
BASDAI	1.01	1.00	1.01
DiseaseDuration_2	0.59	0.38	0.89
DiseaseDuration_3	0.98	0.68	1.42
DiseaseDuration_4	0.91	0.60	1.39
DiseaseDuration_5	0.52	0.16	1.35
CalendarYear	1.08	1.01	1.15
Czech Republic (CZ)			
Denmark (DK)	0.89	0.08	19.87
Spain (ES)			
Iceland (IS)	1.67	0.07	59.68
Italy (IT)			
Netherlands (NL)			
Norway (NO)			
Portugal (PT)	0.12	0.01	2.73
Romania (RO)			
Finland (SF)			
Slovenia (SI)			
Turkey (TR)	0.93	0.02	39.99
United Kingdom (UK)	0.60	0.05	13.61

Model 3a/3b ---------------------- Model 2 additionally adjusted, in turn, for Smoking and BMI

Individual models. Result = OR (Alcohol) from each model

		OR (alcohol)	95%CI-Lo	95%CI-Hi	OR↑↓	N	N↓
(3a)	Smoking	1.54	1.14	2.08	0.031	1234	3 (0%)
(3b)	BMI	1.65	1.20	2.27	0.076	1033	204 (16%)

Sensitivity analysis -------------- Model 2 additionally adjusted, in turn, for other variables shown

Individual models. Result = OR (Alcohol) from each model

	OR (alcohol)	95%CI-Lo	95%CI-Hi	OR↑↓	N	N↓
Yrs since symptom onset	1.75	1.28	2.41	0.182	1071	166 (13%)
HLA-B27 status	1.54	1.04	2.32	0.024	726	511 (41%)
ESR	1.33	0.41	4.38	0.236	324	913 (74%)
CRP	1.70	1.23	2.36	0.133	1123	114 (9%)
mNY criteria	1.52	0.98	2.37	0.046	651	586 (47%)
ASAS criteria	1.59	1.09	2.32	0.016	803	434 (35%)
IBD	1.67	1.14	2.45	0.101	802	435 (35%)
Uveitis	1.51	1.04	2.22	0.056	798	439 (35%)
Psoriasis	1.64	1.12	2.40	0.068	824	413 (33%)
Comorbidity cardiovascular	1.60	0.82	3.19	0.027	392	845 (68%)
Comorbidity kidney	2.28	1.06	5.07	0.706	357	880 (71%)
Comorbidity diabetes	1.71	0.85	3.52	0.142	393	844 (68%)
BASFI	1.54	1.14	2.08	0.031	1215	22 (2%)
BASMI (3pt)	1.43	0.92	2.24	0.135	537	700 (57%)
Patient global	1.55	1.15	2.10	0.018	1234	3 (0%)
Physician global	1.44	1.04	2.00	0.133	975	262 (21%)
Pain (VAS)	1.60	1.18	2.17	0.027	1000	237 (19%)
Fatigue	1.52	1.13	2.06	0.047	1225	12 (1%)
HAQ	1.31	0.81	2.11	0.263	389	848 (69%)
csDMARD	2.07	1.21	3.60	0.498	459	778 (63%)
NSAID	1.90	1.11	3.29	0.331	549	688 (56%)

= Highest OR value
= Lowest OR value

Supplementary data: Association between ALCOHOL at baseline and BASDAI-50 at 12 months

Model 1 ---------------------------- Crude (unadjusted) association

Exposure	Alcohol
Outcome	BASDAI-50
Timepoint	12 months
N (countries)	7
N (patients)	1614

		BASDAI-50	
		No	Yes
Alcohol	No	491	367
	Yes	415	341

OR	95%CI-Lo	95%CI-Hi
1.10	0.90	1.34

Model 2 ---------------------------- Model 1, adjusted for: age, gender, country, calendar year, disease duration and disease activity

N (countries)	7
N (patients)	1614

Reference country = Switzerland (CH)
Blank line = No data for this analysis

	OR	95%CI-Lo	95%CI-Hi
Alcohol = Yes	1.47	1.16	1.88
Age	0.97	0.96	0.98
Male	1.75	1.41	2.17
BASDAI	1.01	1.00	1.01
DiseaseDuration_2	0.75	0.55	1.03
DiseaseDuration_3	0.86	0.65	1.13
DiseaseDuration_4	0.95	0.70	1.29
DiseaseDuration_5	1.14	0.61	2.11
CalendarYear	0.98	0.94	1.02
Czech Republic (CZ)			
Denmark (DK)	1.12	0.24	5.78
Spain (ES)			
Iceland (IS)	2.20	0.30	17.86
Italy (IT)	2.23	0.44	12.63
Netherlands (NL)			
Norway (NO)			
Portugal (PT)	2.28	0.49	11.94
Romania (RO)			
Finland (SF)			
Slovenia (SI)			
Turkey (TR)	21.99	2.11	550.69
United Kingdom (UK)	1.16	0.24	6.17

Model 3a/3b ---------------------- Model 2 additionally adjusted, in turn, for Smoking and BMI

Individual models. Result = OR (Alcohol) from each model

		OR (alcohol)	95%CI-Lo	95%CI-Hi	OR↑↓	N	N↓
(3a)	Smoking	1.46	1.15	1.87	0.006	1610	4 (0%)
(3b)	BMI	1.50	1.15	1.96	0.031	1267	347 (21%)

Sensitivity analysis -------------- Model 2 additionally adjusted, in turn, for other variables shown

Individual models. Result = OR (Alcohol) from each model

	OR (alcohol)	95%CI-Lo	95%CI-Hi	OR↑↓	N	N↓
Yrs since symptom onset	1.55	1.20	2.02	0.081	1392	222 (14%)
HLA-B27 status	1.64	1.21	2.24	0.172	1051	563 (35%)
ESR	1.13	0.67	1.90	0.343	570	1044 (65%)
CRP	1.51	1.16	1.97	0.036	1406	208 (13%)
mNY criteria	1.54	1.11	2.16	0.071	926	688 (43%)
ASAS criteria	1.64	1.21	2.21	0.164	1101	513 (32%)
IBD	1.72	1.27	2.34	0.252	1101	513 (32%)
Uveitis	1.62	1.20	2.20	0.147	1103	511 (32%)
Psoriasis	1.73	1.28	2.34	0.254	1125	489 (30%)
Comorbidity cardiovascular	1.22	0.75	1.99	0.251	579	1035 (64%)
Comorbidity kidney	1.43	0.86	2.41	0.040	541	1073 (66%)
Comorbidity diabetes	1.30	0.80	2.13	0.171	568	1046 (65%)
BASFI	1.38	1.08	1.77	0.090	1555	59 (4%)
BASMI (3pt)	1.44	1.00	2.09	0.029	720	894 (55%)
Patient global	1.54	1.20	1.97	0.064	1576	38 (2%)
Physician global	1.61	1.22	2.12	0.136	1261	353 (22%)
Pain (VAS)	1.68	1.29	2.21	0.212	1107	507 (31%)
Fatigue	1.52	1.18	1.94	0.044	1567	47 (3%)
HAQ	1.32	0.85	2.06	0.149	444	1170 (72%)
csDMARD	1.53	1.00	2.38	0.063	603	1011 (63%)
NSAID	1.54	1.03	2.32	0.070	781	833 (52%)

= Highest OR value
= Lowest OR value

Supplementary data: Association between ALCOHOL at baseline and ASDAS-Inactive at 12 months

Model 1 ---------------------------- Crude (unadjusted) association

Exposure	Alcohol
Outcome	ASDAS-Inactive
Timepoint	12 months
N (countries)	7
N (patients)	1996

		ASDAS-Inactive	
		No	Yes
Alcohol	No	841	213
	Yes	693	249

OR	95%CI-Lo	95%CI-Hi
1.42	1.15	1.75

Model 2 ---------------------------- Model 1, adjusted for: age, gender, country, calendar year, disease duration and disease activity

N (countries)	6
N (patients)	1410

Reference country = Switzerland (CH)
Blank line = No data for this analysis

	OR	95%CI-Lo	95%CI-Hi
Alcohol = Yes	1.60	1.17	2.18
Age	0.96	0.94	0.97
Male	1.74	1.31	2.33
BASDAI	0.97	0.97	0.98
DiseaseDuration_2	1.06	0.71	1.57
DiseaseDuration_3	1.13	0.79	1.62
DiseaseDuration_4	1.15	0.76	1.73
DiseaseDuration_5	0.58	0.21	1.39
CalendarYear	0.99	0.93	1.04
Czech Republic (CZ)			
Denmark (DK)	2.14	0.34	41.55
Spain (ES)			
Iceland (IS)			
Italy (IT)	6.15	0.77	131.84
Netherlands (NL)			
Norway (NO)			
Portugal (PT)	3.35	0.53	65.44
Romania (RO)			
Finland (SF)			
Slovenia (SI)			
Turkey (TR)	3.11	0.29	76.53
United Kingdom (UK)	2.65	0.40	52.49

Model 3a/3b ---------------------- Model 2 additionally adjusted, in turn, for Smoking and BMI

Individual models. Result = OR (Alcohol) from each model

		OR (alcohol)	95%CI-Lo	95%CI-Hi	OR↑↓	N	N↓
(3a)	Smoking	1.59	1.69	2.18	0.002	1406	4 (0%)
(3b)	BMI	1.76	1.25	2.50	0.165	1098	312 (22%)

Sensitivity analysis -------------- Model 2 additionally adjusted, in turn, for other variables shown

Individual models. Result = OR (Alcohol) from each model

	OR (alcohol)	95%CI-Lo	95%CI-Hi	OR↑↓	N	N↓
Yrs since symptom onset	1.65	1.19	2.30	0.057	1242	168 (12%)
HLA-B27 status	1.46	0.99	2.16	0.132	911	499 (35%)
ESR	1.06	0.56	1.98	0.536	501	909 (64%)
CRP	1.58	1.14	2.20	0.018	1267	143 (10%)
mNY criteria	1.65	1.08	2.55	0.056	807	603 (43%)
ASAS criteria	1.54	1.06	2.27	0.051	974	436 (31%)
IBD	1.44	0.98	2.13	0.154	973	437 (31%)
Uveitis	1.46	0.99	2.15	0.132	979	431 (31%)
Psoriasis	1.43	0.97	2.11	0.164	999	411 (29%)
Comorbidity cardiovascular	1.10	0.60	2.02	0.496	519	891 (63%)
Comorbidity kidney	0.99	0.53	1.80	0.611	483	927 (66%)
Comorbidity diabetes	0.99	0.53	1.81	0.607	507	903 (64%)
BASFI	1.46	1.06	2.02	0.132	1373	37 (3%)
BASMI (3pt)	1.35	0.84	2.19	0.242	651	759 (54%)
Patient global	1.62	1.19	2.23	0.029	1384	26 (2%)
Physician global	1.77	1.24	2.53	0.171	1109	301 (21%)
Pain (VAS)	2.06	1.44	2.97	0.460	958	452 (32%)
Fatigue	1.65	1.21	2.27	0.057	1389	21 (1%)
HAQ	1.35	0.75	2.45	0.247	369	1041 (74%)
csDMARD	1.79	1.05	3.09	0.190	531	879 (62%)
NSAID	1.14	0.70	1.84	0.459	700	710 (50%)

(red) = Highest OR value
(blue) = Lowest OR value

Supplementary data: Association between ALCOHOL at baseline and ASDAS-LDA at 12 months

Model 1 ---------------------------- Crude (unadjusted) association

Exposure	Alcohol
Outcome	ASDAS-LDA
Timepoint	12 months
N (countries)	7
N (patients)	1996

		ASDAS-LDA No	ASDAS-LDA Yes
Alcohol	No	563	491
	Yes	404	538

OR	95%CI-Lo	95%CI-Hi
1.53	1.28	1.82

Model 2 ---------------------------- Model 1, adjusted for: age, gender, country, calendar year, disease duration and disease activity

N (countries) 7
N (patients) 1410

Reference country = Switzerland (CH)
Blank line = No data for this analysis

	OR	95%CI-Lo	95%CI-Hi
Alcohol = Yes	1.54	1.18	2.02
Age	0.97	0.96	0.98
Male	1.43	1.13	1.81
BASDAI	0.97	0.96	0.97
DiseaseDuration_2	0.80	0.57	1.14
DiseaseDuration_3	0.89	0.65	1.21
DiseaseDuration_4	0.97	0.69	1.37
DiseaseDuration_5	1.56	0.76	3.34
CalendarYear	0.97	0.93	1.02
Czech Republic (CZ)			
Denmark (DK)	0.91	0.17	4.58
Spain (ES)			
Iceland (IS)	0.52	0.05	4.53
Italy (IT)	1.08	0.16	6.97
Netherlands (NL)			
Norway (NO)			
Portugal (PT)	1.80	0.32	9.16
Romania (RO)			
Finland (SF)			
Slovenia (SI)			
Turkey (TR)	3.12	0.30	39.62
United Kingdom (UK)	1.18	0.21	6.13

Model 3a/3b ---------------------- Model 2 additionally adjusted, in turn, for Smoking and BMI

Individual models. Result = OR (Alcohol) from each model

		OR (alcohol)	95%CI-Lo	95%CI-Hi	OR↑↓	N	N↓
(3a)	Smoking	1.53	1.17	2.01	0.014	1406	4 (0%)
(3b)	BMI	1.62	1.21	2.19	0.080	1098	312 (22%)

Sensitivity analysis -------------- Model 2 additionally adjusted, in turn, for other variables shown

Individual models. Result = OR (Alcohol) from each model

	OR (alcohol)	95%CI-Lo	95%CI-Hi	OR↑↓	N	N↓
Yrs since symptom onset	1.71	1.28	2.28	0.163	1242	168 (12%)
HLA-B27 status	1.65	1.17	2.35	0.109	911	499 (35%)
ESR	1.11	0.63	1.96	0.438	501	909 (64%)
CRP	1.61	1.21	2.15	0.067	1267	143 (10%)
mNY criteria	1.51	1.05	2.19	0.034	807	603 (43%)
ASAS criteria	1.61	1.16	2.25	0.071	974	436 (31%)
IBD	1.57	1.12	2.20	0.025	973	437 (31%)
Uveitis	1.56	1.11	2.18	0.012	979	431 (31%)
Psoriasis	1.57	1.13	2.19	0.023	999	411 (29%)
Comorbidity cardiovascular	1.54	0.89	2.67	0.008	519	891 (63%)
Comorbidity kidney	1.62	0.92	2.87	0.073	483	927 (66%)
Comorbidity diabetes	1.58	0.92	2.76	0.040	507	903 (64%)
BASFI	1.43	1.09	1.89	0.114	1373	37 (3%)
BASMI (3pt)	1.24	0.83	1.87	0.302	651	759 (54%)
Patient global	1.55	1.18	2.03	0.002	1384	26 (2%)
Physician global	1.47	1.09	1.98	0.077	1109	301 (21%)
Pain (VAS)	1.78	1.32	2.41	0.236	958	452 (32%)
Fatigue	1.61	1.22	2.11	0.064	1389	21 (1%)
HAQ	1.13	0.69	1.86	0.415	369	1041 (74%)
csDMARD	1.80	1.11	2.94	0.258	531	879 (62%)
NSAID	1.31	0.85	2.04	0.235	700	710 (50%)

= Highest OR value
= Lowest OR value

Supplementary data: Association between ALCOHOL at baseline and ASDAS-CII at 12 months

Model 1 ---------------------------- Crude (unadjusted) association

Exposure	Alcohol
Outcome	ASDAS-CII
Timepoint	12 months
N (countries)	7
N (patients)	1269

		ASDAS-CII	
		No	Yes
Alcohol	No	318	384
	Yes	279	288

OR	95%CI-Lo	95%CI-Hi
0.85	0.68	1.07

Model 2 ---------------------------- Model 1, adjusted for: age, gender, country, calendar year, disease duration and disease activity

N (countries)	7
N (patients)	1269

Reference country = Switzerland (CH)
Blank line = No data for this analysis

	OR	95%CI-Lo	95%CI-Hi
Alcohol = Yes	1.24	0.94	1.64
Age	0.98	0.97	0.99
Male	1.81	1.42	2.32
BASDAI	1.02	1.02	1.03
DiseaseDuration_2	0.76	0.53	1.09
DiseaseDuration_3	0.87	0.63	1.19
DiseaseDuration_4	0.90	0.63	1.28
DiseaseDuration_5	1.02	0.48	2.20
CalendarYear	1.01	0.96	1.06
Czech Republic (CZ)			
Denmark (DK)	0.75	0.14	3.71
Spain (ES)			
Iceland (IS)	0.48	0.04	4.91
Italy (IT)	1.61	0.23	10.74
Netherlands (NL)			
Norway (NO)			
Portugal (PT)	1.69	0.30	8.45
Romania (RO)			
Finland (SF)			
Slovenia (SI)			
Turkey (TR)	2.14	0.26	17.28
United Kingdom (UK)	1.07	0.19	5.51

Model 3a/3b ---------------------- Model 2 additionally adjusted, in turn, for Smoking and BMI

Individual models. Result = OR (Alcohol) from each model

		OR (alcohol)	95%CI-Lo	95%CI-Hi	OR↑↓	N	N↓
(3a)	Smoking	1.24	0.94	1.64	0.001	1265	4 (0%)
(3b)	BMI	1.31	0.97	1.77	0.066	976	293 (23%)

Sensitivity analysis --------------- Model 2 additionally adjusted, in turn, for other variables shown

Individual models. Result = OR (Alcohol) from each model

	OR (alcohol)	95%CI-Lo	95%CI-Hi	OR↑↓	N	N↓
Yrs since symptom onset	1.37	1.02	1.84	0.125	1129	140 (11%)
HLA-B27 status	1.31	0.92	1.87	0.072	829	440 (35%)
ESR	1.28	0.70	2.39	0.040	488	781 (62%)
CRP	1.39	1.03	1.89	0.153	1241	28 (2%)
mNY criteria	1.39	0.94	2.06	0.145	734	535 (42%)
ASAS criteria	1.37	0.97	1.92	0.124	883	386 (30%)
IBD	1.36	0.96	1.92	0.117	881	388 (31%)
Uveitis	1.28	0.91	1.81	0.040	890	379 (30%)
Psoriasis	1.31	0.93	1.83	0.064	907	362 (29%)
Comorbidity cardiovascular	1.14	0.67	1.97	0.100	486	783 (62%)
Comorbidity kidney	1.22	0.68	2.20	0.024	451	818 (64%)
Comorbidity diabetes	1.06	0.61	1.86	0.180	474	795 (63%)
BASFI	1.20	0.91	1.60	0.037	1238	31 (2%)
BASMI (3pt)	1.20	0.81	1.80	0.038	616	653 (51%)
Patient global	1.27	0.96	1.68	0.027	1267	2 (0%)
Physician global	1.28	0.94	1.75	0.037	1012	257 (20%)
Pain (VAS)	1.41	1.04	1.93	0.171	846	423 (33%)
Fatigue	1.24	0.94	1.65	0.003	1252	17 (1%)
HAQ	0.97	0.59	1.60	0.267	336	933 (74%)
csDMARD	1.45	0.89	2.37	0.204	482	787 (62%)
NSAID	1.04	0.66	1.66	0.198	642	627 (49%)

= Highest OR value
= Lowest OR value

Supplementary data: Association between ALCOHOL at baseline and ASDAS-MI at 12 months

Model 1 ---------------------------- Crude (unadjusted) association

Exposure	Alcohol
Outcome	ASDAS-MI
Timepoint	12 months
N (countries)	7
N (patients)	1269

		ASDAS-MI	
		No	Yes
Alcohol	No	500	202
	Yes	421	146

OR	95%CI-Lo	95%CI-Hi
0.86	0.67	1.10

Model 2 ---------------------------- Model 1, adjusted for: age, gender, country, calendar year, disease duration and disease activity

N (countries)	7
N (patients)	1269

Reference country = Switzerland (CH)
Blank line = No data for this analysis

	OR	95%CI-Lo	95%CI-Hi
Alcohol = Yes	1.28	0.94	1.75
Age	0.97	0.96	0.98
Male	1.46	1.11	1.92
BASDAI	1.03	1.02	1.04
DiseaseDuration_2	0.65	0.43	0.97
DiseaseDuration_3	0.98	0.70	1.38
DiseaseDuration_4	0.90	0.61	1.34
DiseaseDuration_5	0.85	0.34	1.94
CalendarYear	0.99	0.93	1.04
Czech Republic (CZ)			
Denmark (DK)	1.06	0.21	7.83
Spain (ES)			
Iceland (IS)	0.31	0.01	4.82
Italy (IT)	0.22	0.01	3.04
Netherlands (NL)			
Norway (NO)			
Portugal (PT)	1.85	0.36	13.72
Romania (RO)			
Finland (SF)			
Slovenia (SI)			
Turkey (TR)	2.83	0.30	30.40
United Kingdom (UK)	1.10	0.20	8.39

Model 3a/3b ---------------------- Model 2 additionally adjusted, in turn, for Smoking and BMI

Individual models. Result = OR (Alcohol) from each model

		OR (alcohol)	95%CI-Lo	95%CI-Hi	OR↑↓	N	N↓
(3a)	Smoking	1.28	0.94	1.75	0.001	1265	4 (0%)
(3b)	BMI	1.34	0.95	1.91	0.063	976	293 (23%)

Sensitivity analysis -------------- Model 2 additionally adjusted, in turn, for other variables shown

Individual models. Result = OR (Alcohol) from each model

	OR (alcohol)	95%CI-Lo	95%CI-Hi	OR↑↓	N	N↓
Yrs since symptom onset	1.41	1.02	1.96	0.131	1129	140 (11%)
HLA-B27 status	1.36	0.92	2.03	0.085	829	440 (35%)
ESR	1.26	0.64	2.46	0.023	488	781 (62%)
CRP	1.56	1.10	2.23	0.280	1241	28 (2%)
mNY criteria	1.12	0.74	1.72	0.155	734	535 (42%)
ASAS criteria	1.31	0.90	1.91	0.028	883	386 (30%)
IBD	1.31	0.89	1.92	0.029	881	388 (31%)
Uveitis	1.20	0.82	1.76	0.080	890	379 (30%)
Psoriasis	1.26	0.86	1.85	0.021	907	362 (29%)
Comorbidity cardiovascular	0.85	0.47	1.53	0.429	486	783 (62%)
Comorbidity kidney	1.06	0.57	1.98	0.220	451	818 (64%)
Comorbidity diabetes	0.97	0.53	1.76	0.310	474	795 (63%)
BASFI	1.28	0.93	1.76	0.001	1238	31 (2%)
BASMI (3pt)	1.19	0.76	1.87	0.087	616	653 (51%)
Patient global	1.29	0.94	1.77	0.007	1267	2 (0%)
Physician global	1.51	1.06	2.17	0.235	1012	257 (20%)
Pain (VAS)	1.38	0.97	1.98	0.103	846	423 (33%)
Fatigue	1.25	0.91	1.72	0.030	1252	17 (1%)
HAQ	1.29	0.73	2.33	0.014	336	933 (74%)
csDMARD	1.27	0.74	2.20	0.013	482	787 (62%)
NSAID	1.06	0.65	1.73	0.221	642	627 (49%)

(red) = Highest OR value
(blue) = Lowest OR value

Supplementary data: Association between ALCOHOL at baseline and ASAS-20 at 12 months

Model 1 ---------------------------- Crude (unadjusted) association

Exposure	Alcohol
Outcome	ASAS-20
Timepoint	12 months
N (countries)	7
N (patients)	1388

		ASAS-20	
		No	Yes
Alcohol	No	412	198
	Yes	409	369

OR	95%CI-Lo	95%CI-Hi
1.88	1.51	2.34

Model 2 ---------------------------- Model 1, adjusted for: age, gender, country, calendar year, disease duration and disease activity

N (countries)	5
N (patients)	1221

Reference country = Switzerland (CH)
Blank line = No data for this analysis

	OR	95%CI-Lo	95%CI-Hi
Alcohol = Yes	1.72	1.31	2.26
Age	0.98	0.97	0.99
Male	1.27	0.98	1.66
BASDAI	1.02	1.02	1.03
DiseaseDuration_2	0.61	0.42	0.88
DiseaseDuration_3	0.77	0.55	1.08
DiseaseDuration_4	0.93	0.64	1.37
DiseaseDuration_5	0.71	0.29	1.75
CalendarYear	0.97	0.92	1.03
Czech Republic (CZ)			
Denmark (DK)	0.81	0.16	3.44
Spain (ES)			
Iceland (IS)	0.54	0.06	4.22
Italy (IT)			
Netherlands (NL)			
Norway (NO)			
Portugal (PT)			
Romania (RO)			
Finland (SF)			
Slovenia (SI)			
Turkey (TR)	15.80	1.45	384.85
United Kingdom (UK)	1.22	0.23	5.43

Model 3a/3b ---------------------- Model 2 additionally adjusted, in turn, for Smoking and BMI

Individual models. Result = OR (Alcohol) from each model

		OR (alcohol)	95%CI-Lo	95%CI-Hi	OR↑↓	N	N↓
(3a)	Smoking	1.70	1.30	2.23	0.021	1219	2 (0%)
(3b)	BMI	1.72	1.30	2.30	0.003	1087	134 (11%)

Sensitivity analysis -------------- Model 2 additionally adjusted, in turn, for other variables shown

Individual models. Result = OR (Alcohol) from each model

	OR (alcohol)	95%CI-Lo	95%CI-Hi	OR↑↓	N	N↓
Yrs since symptom onset	1.81	1.35	2.44	0.091	1056	165 (14%)
HLA-B27 status	1.63	1.13	2.36	0.090	707	514 (42%)
ESR	4.27	1.13	17.82	2.555	214	1007 (82%)
CRP	1.63	1.22	2.19	0.088	1072	149 (12%)
mNY criteria	1.47	0.99	2.20	0.246	615	606 (50%)
ASAS criteria	1.58	1.12	2.23	0.139	790	431 (35%)
IBD	1.77	1.25	2.52	0.054	776	445 (36%)
Uveitis	1.44	1.02	2.05	0.277	779	442 (36%)
Psoriasis	1.54	1.10	2.17	0.176	802	419 (34%)
Comorbidity cardiovascular	1.58	1.00	2.49	0.141	313	908 (74%)
Comorbidity kidney	2.31	1.00	5.33	0.589	275	946 (77%)
Comorbidity diabetes	1.82	1.00	3.31	0.101	304	917 (75%)
BASFI	1.65	1.26	2.18	0.065	1201	20 (2%)
BASMI (3pt)	1.79	1.20	2.69	0.067	544	677 (55%)
Patient global	1.75	1.33	2.31	0.032	1214	7 (1%)
Physician global	1.77	1.31	2.39	0.048	1011	210 (17%)
Pain (VAS)	1.73	1.32	2.29	0.015	1073	148 (12%)
Fatigue	1.71	1.31	2.25	0.008	1213	8 (1%)
HAQ	1.37	0.88	2.14	0.352	412	809 (66%)
csDMARD	1.41	0.85	2.34	0.309	418	803 (66%)
NSAID	2.21	1.27	3.91	0.495	438	783 (64%)

(red) = Highest OR value
(blue) = Lowest OR value

Supplementary data: Association between ALCOHOL at baseline and ASAS-40 at 12 months

Model 1 ---------------------------- Crude (unadjusted) association

Exposure	Alcohol
Outcome	ASAS-40
Timepoint	12 months
N (countries)	7
N (patients)	1561

		ASAS-40 No	ASAS-40 Yes
Alcohol	No	583	130
	Yes	590	258

OR	95%CI-Lo	95%CI-Hi
1.96	1.55	2.50

Model 2 ---------------------------- Model 1, adjusted for: age, gender, country, calendar year, disease duration and disease activity

N (countries)	7
N (patients)	1313

Reference country = Switzerland (CH)

Blank line = No data for this analysis

		OR	95%CI-Lo	95%CI-Hi
	Alcohol = Yes	1.74	1.31	2.31
	Age	0.98	0.96	0.99
	Male	1.36	1.03	1.79
	BASDAI	1.02	1.01	1.03
	DiseaseDuration_2	0.68	0.46	0.99
	DiseaseDuration_3	0.65	0.46	0.91
	DiseaseDuration_4	0.76	0.52	1.13
	DiseaseDuration_5	0.59	0.21	1.49
	CalendarYear	1.02	0.96	1.08
	Czech Republic (CZ)			
	Denmark (DK)	0.93	0.21	4.74
	Spain (ES)			
	Iceland (IS)	0.66	0.08	5.41
Rounding to 2dp. Actual value = non-zero.	Italy (IT)	0.00	0.00	0.00
	Netherlands (NL)			
	Norway (NO)			
Rounding to 2dp. Actual value = non-zero.	Portugal (PT)	0.00	0.00	0.00
	Romania (RO)			
	Finland (SF)			
	Slovenia (SI)			
	Turkey (TR)	5.29	0.74	43.22
	United Kingdom (UK)	1.17	0.26	6.17

Model 3a/3b ---------------------- Model 2 additionally adjusted, in turn, for Smoking and BMI

Individual models. Result = OR (Alcohol) from each model

		OR (alcohol)	95%CI-Lo	95%CI-Hi	OR↑↓	N	N↓
(3a)	Smoking	1.71	1.29	2.28	0.032	1311	2 (0%)
(3b)	BMI	1.66	1.23	2.24	0.080	1127	186 (14%)

Sensitivity analysis -------------- Model 2 additionally adjusted, in turn, for other variables shown

Individual models. Result = OR (Alcohol) from each model

	OR (alcohol)	95%CI-Lo	95%CI-Hi	OR↑↓	N	N↓
Yrs since symptom onset	1.84	1.36	2.50	0.099	1140	173 (13%)
HLA-B27 status	1.84	1.33	2.88	0.099	784	529 (40%)
ESR	21.71	3.52	439.30	19.967	295	1018 (78%)
CRP	1.68	1.24	2.29	0.060	1155	158 (12%)
mNY criteria	1.76	1.16	2.69	0.017	680	633 (48%)
ASAS criteria	1.83	1.28	2.64	0.096	864	449 (34%)
IBD	1.98	1.37	2.87	0.238	849	464 (35%)
Uveitis	1.78	1.23	2.57	0.037	852	461 (35%)
Psoriasis	1.87	1.30	2.70	0.130	875	438 (33%)
Comorbidity cardiovascular	2.73	1.00	7.46	0.993	373	940 (72%)
Comorbidity kidney	7.64	1.00	58.31	5.898	332	981 (75%)
Comorbidity diabetes	3.42	1.00	11.67	1.679	361	952 (73%)
BASFI	1.66	1.24	2.21	0.083	1284	29 (2%)
BASMI (3pt)	1.34	0.88	2.05	0.400	587	726 (55%)
Patient global	1.75	1.31	2.34	0.011	1304	9 (1%)
Physician global	1.58	1.16	2.15	0.162	1077	236 (18%)
Pain (VAS)	1.77	1.33	2.38	0.035	1085	228 (17%)
Fatigue	1.73	1.30	2.30	0.012	1300	13 (1%)
HAQ	1.21	0.76	1.92	0.533	419	894 (68%)
csDMARD	2.23	1.31	3.89	0.493	465	848 (65%)
NSAID	4.35	2.34	8.44	2.615	518	795 (61%)

= Highest OR value

= Lowest OR value

Supplementary data: Association between ALCOHOL at baseline and ASAS-5of6 at 12 months

Model 1 ---------------------------- Crude (unadjusted) association

Exposure	Alcohol
Outcome	ASAS-5of6
Timepoint	12 months
N (countries)	7
N (patients)	1091

		ASAS-5of6 No	ASAS-5of6 Yes
Alcohol	No	425	116
	Yes	386	164

OR	95%CI-Lo	95%CI-Hi
1.56	1.18	2.05

Model 2 ---------------------------- Model 1, adjusted for: age, gender, country, calendar year, disease duration and disease activity

N (countries) 6
N (patients) 934

Reference country = Switzerland (CH)
Blank line = No data for this analysis

	OR	95%CI-Lo	95%CI-Hi
Alcohol = Yes	1.40	1.02	1.94
Age	0.97	0.96	0.99
Male	1.40	1.02	1.92
BASDAI	1.01	1.00	1.02
DiseaseDuration_2	0.54	0.34	0.84
DiseaseDuration_3	0.77	0.52	1.14
DiseaseDuration_4	0.79	0.50	1.24
DiseaseDuration_5	0.61	0.19	1.72
CalendarYear	1.02	0.95	1.10
Czech Republic (CZ)			
Denmark (DK)	0.81	0.17	4.32
Spain (ES)			
Iceland (IS)	0.82	0.07	9.21
Italy (IT)			
Netherlands (NL)			
Norway (NO)			
Portugal (PT)	0.12	0.02	0.73
Romania (RO)			
Finland (SF)			
Slovenia (SI)			
Turkey (TR)	5.82	0.64	69.21
United Kingdom (UK)	0.79	0.16	4.35

Model 3a/3b ---------------------- Model 2 additionally adjusted, in turn, for Smoking and BMI

Individual models. Result = OR (Alcohol) from each model

		OR (alcohol)	95%CI-Lo	95%CI-Hi	OR↑↓	N	N↓
(3a)	Smoking	1.37	0.99	1.90	0.032	932	2 (0%)
(3b)	BMI	1.38	0.98	1.95	0.025	795	139 (15%)

Sensitivity analysis -------------- Model 2 additionally adjusted, in turn, for other variables shown

Individual models. Result = OR (Alcohol) from each model

	OR (alcohol)	95%CI-Lo	95%CI-Hi	OR↑↓	N	N↓
Yrs since symptom onset	1.53	1.08	2.18	0.131	813	121 (13%)
HLA-B27 status	1.52	0.97	2.39	0.116	539	395 (42%)
ESR	2.52	0.69	9.20	1.120	216	718 (77%)
CRP	1.47	1.03	2.11	0.071	868	66 (7%)
mNY criteria	1.42	0.87	2.34	0.021	479	455 (49%)
ASAS criteria	1.50	0.99	2.29	0.100	604	330 (35%)
IBD	1.64	1.08	2.53	0.242	598	336 (36%)
Uveitis	1.30	0.85	1.99	0.105	601	333 (36%)
Psoriasis	1.35	0.90	2.05	0.050	626	308 (33%)
Comorbidity cardiovascular	1.23	0.54	2.83	0.169	270	664 (71%)
Comorbidity kidney	2.38	0.93	6.26	0.981	238	696 (75%)
Comorbidity diabetes	1.98	0.83	4.81	0.577	261	673 (72%)
BASFI	1.37	0.99	1.90	0.037	917	17 (2%)
BASMI (3pt)	1.26	0.79	2.00	0.148	468	466 (50%)
Patient global	1.48	1.06	2.06	0.073	929	5 (1%)
Physician global	1.39	0.98	1.98	0.013	772	162 (17%)
Pain (VAS)	1.43	1.03	2.01	0.031	755	179 (19%)
Fatigue	1.37	0.99	1.90	0.030	929	5 (1%)
HAQ	1.06	0.62	1.83	0.344	298	636 (68%)
csDMARD	1.67	0.93	3.05	0.269	344	590 (63%)
NSAID	1.62	0.86	3.06	0.214	382	552 (59%)

= Highest OR value (red)
= Lowest OR value (blue)

Supplementary data: CRediT statement

Author contributions, as defined by CRediT (Contributor Roles Taxonomy; https://www.elsevier.com/authors/policies-and-guidelines/credit-author-statement)

	Conceptualisation	Methodology	Software	Validation	Formal analysis	Investigation	Resources	Data curation	Writing (Original draft)	Writing (Review and editing)	Visualisation	Supervision	Project administration	Funding acquisition	Author
Gareth T Jones	✓	✓		✓	✓	✓			✓	✓	✓		✓	✓	✓
Ovidiu Rotariu		✓	✓		✓			✓	✓	✓					✓
Ross MacDonald			✓		✓					✓					✓
Brigitte Michelsen	✓	✓				✓	✓			✓			✓		✓
Bente Glintborg	✓	✓				✓	✓			✓			✓		✓
Irene van der Horst-Bruinsma						✓	✓			✓		✓			✓
Bjorn Gudbjornsson						✓	✓			✓		✓			✓
Arni Jon Geirsson						✓	✓			✓		✓			✓
Heikki Relas						✓	✓			✓		✓			✓
Pia Isomäki						✓	✓			✓		✓			✓
Jakub Závada						✓	✓			✓		✓			✓
Karel Pavelka						✓	✓			✓		✓			✓
Ziga Rotar						✓	✓			✓		✓			✓
Matija Tomsic						✓	✓			✓		✓			✓
Michael J Nissen						✓	✓			✓		✓			✓
Adrian Ciurea						✓	✓			✓		✓			✓
Catalin Codreanu						✓	✓			✓		✓			✓
Johan K Wallman						✓	✓			✓		✓			✓
Eirik Klami Kristianslund						✓	✓			✓		✓			✓
Simon Horskjær Rasmussen			✓			✓		✓		✓		✓			✓
Lykke Midtbøll Ørnbjerg	✓		✓			✓	✓	✓		✓		✓			✓
Maria José Santos						✓	✓			✓		✓			✓
Mikkel Østergaard	✓					✓	✓			✓		✓		✓	✓
Merete L Hetland	✓					✓	✓			✓		✓		✓	✓
Gary J Macfarlane	✓	✓				✓				✓			✓	✓	✓
Linda E Dean		✓	✓	✓	✓										

Term	Definition
Conceptualisation	Ideas; formulation or evolution of overarching research goals and aims
Methodology	Development or design of methodology; creation of models
Software	Programming, software development; designing computer programs; implementation of the computer code and supporting algorithms; testing of existing code components
Validation	Verification, whether as a part of the activity or separate, of the overall replication/ reproducibility of results/experiments and other research outputs
Formal analysis	Application of statistical, mathematical, computational, or other formal techniques to analyse or synthesize study data
Investigation	Conducting a research and investigation process, specifically performing the experiments, or data/evidence collection
Resources	Provision of study materials, reagents, materials, patients, laboratory samples, animals, instrumentation, computing resources, or other analysis tools
Data curation	Management activities to annotate (produce metadata), scrub data and maintain research data (including software code, where it is necessary for interpreting the data itself) for initial use and later reuse
Writing (Original draft)	Preparation, creation and/or presentation of the published work, specifically writing the initial draft (including substantive translation)
Writing (Review and editing)	Preparation, creation and/or presentation of the published work by those from the original research group, specifically critical review, commentary or revision – including pre- or post-publication stages
Visualisation	Preparation, creation and/or presentation of the published work, specifically visualization/ data presentation
Supervision	Oversight and leadership responsibility for the research activity planning and execution, including mentorship external to the core team
Project administration	Management and coordination responsibility for the research activity planning and execution
Funding acquisition	Acquisition of the financial support for the project leading to this publication
